# A deep learning framework assisted echocardiography with diagnosis, lesion localization, phenogrouping heterogeneous disease, and anomaly detection

**DOI:** 10.1038/s41598-022-27211-w

**Published:** 2023-01-02

**Authors:** Bohan Liu, Hao Chang, Dong Yang, Feifei Yang, Qiushuang Wang, Yujiao Deng, Lijun Li, Wenqing Lv, Bo Zhang, Liheng Yu, Daniel Burkhoff, Kunlun He

**Affiliations:** 1 Medical Big Data Research Center, Chinese PLA General Hospital, 28 Fuxing Road, Beijing, 100853 China; 2 Beijing Key Laboratory of Precision Medicine for Chronic Heart Failure, Chinese PLA General Hospital, Beijing, China; 3 Department of Genetics, Howard Hughes Medical Institute, Yale University School of Medicine, New Haven, CT 06510 USA; 4 Key Laboratory of Growth Regulation and Translation Research of Zhejiang Province, School of Life Sciences, Westlake University, Hangzhou, 310024 China; 5 Department of Cardiology, The Fourth Medical Center of Chinese PLA General Hospital, Beijing, 100037 China; 6 Department of Ultrasound Diagnosis, The First Medical Center of Chinese PLA General Hospital, Beijing, 100853 China; 7 Department of Cardiology, The First Medical Center of Chinese PLA General Hospital, Beijing, 100853 China; 8 Department of Cardiology, Beijing Tongren Hospital, Beijing, 100176 China; 9 Department of Ultrasound, Chinese PLA 923 Hospital, Nanning, 530021 China; 10 Division of Cardiology, Columbia University, New York City, NY 10027 USA

**Keywords:** Cardiovascular diseases, Ultrasonography

## Abstract

Echocardiography is the first-line diagnostic technique for heart diseases. Although artificial intelligence techniques have made great improvements in the analysis of echocardiography, the major limitations remain to be the built neural networks are normally adapted to a few diseases and specific equipment. Here, we present an end-to-end deep learning framework named AIEchoDx that differentiates four common cardiovascular diseases (Atrial Septal Defect, Dilated Cardiomyopathy, Hypertrophic Cardiomyopathy, prior Myocardial Infarction) from normal subjects with performance comparable to that of consensus of three senior cardiologists in AUCs (99.50% vs 99.26%, 98.75% vs 92.75%, 99.57% vs 97.21%, 98.52% vs 84.20%, and 98.70% vs 89.41%), respectively. Meanwhile, AIEchoDx accurately recognizes critical lesion regions of interest along with each disease by visualizing the decision-making process. Furthermore, our analysis indicates that heterogeneous diseases, like dilated cardiomyopathy, could be classified into two phenogroups with distinct clinical characteristics. Finally, AIEchoDx performs efficiently as an anomaly detection tool when applying handheld device-produced videos. Together, AIEchoDx provides a potential diagnostic assistant tool in either cart-based echocardiography equipment or handheld echocardiography device for primary and point-of-care medical personnel with high diagnostic performance, and the application of lesion region identification and heterogeneous disease phenogrouping, which may broaden the application of artificial intelligence in echocardiography.

## Introduction

Cardiovascular diseases (CVDs) are increasing threats to global health and have become one of the leading causes of deaths^1^. Early diagnosis and intervention in most CVD conditions have been shown to improve outcomes. While echocardiography is an effective and relatively low-cost noninvasive technique, heart disease diagnosis requires specialists with extensive clinical training and experience, limiting its applications in primary and point-of-care. The recent development of handheld devices^2,3^ could tremendously broaden the application of echocardiography in clinics and even for heart disease identification at home if artificial intelligence (AI)-assistance diagnostic tools comparable to trained cardiologists could be developed.

Recently, AI algorithms have been introduced as a promising solution for the interpretation of static medical images to diagnose various diseases including retinal diseases, central nervous system tumors, acute ischemic stroke, breast cancer, and skin cancer among other conditions^4–8^. Specific advances have also been made in AI analysis of echocardiographic images for cart-based equipment and handheld device separately, including the automated ability to identify the views of echocardiography and automated segmentation of the heart for quantification of parameters such as volumes and ejection fraction^7,9,10^. However, advances in artificial neural network algorithms are still required to achieve diagnostic accuracy at the level of experienced cardiologists and be adapted not only for cart-based equipment but also for the handheld device when analyzing dynamic echocardiographic videos.

In the present study, we collected well-annotated echocardiographic videos and the corresponding medical records for the four common heart diseases and the normal controls: Atrial Septal Defect (ASD), Dilated Cardiomyopathy (DCM), Hypertrophic Cardiomyopathy (HCM), prior Myocardial Infarction (prior MI) and normal subjects (Normal). We retrained the pre-trained Inception-V3 network to extract feature vectors from echocardiographic single-frame images and generated a feature matrix from consecutive frame images to represent a video clip before applying a multiple-layer 1-dimensional convolutional neural network (CNN) model to differentiate and classify patients into the different categories. The two steps in setting up the neural network architecture provide a solution for analyzing echocardiographic dynamic video. We described this new deep learning framework as AI Echocardiogram Diagnosis Network (AIEchoDx). By decoding the features extracted from the CNN, the critical anatomic regions of different cardiac diseases could be identified automatically when combined with class activating mapping^10^. Furthermore, by integrating echocardiographic raw images and electronic medical record data, we identified two phenogroups of DCM patients with significant differences in clinical characteristics. Finally, this deep learning framework provided an accurate interpretation of dynamic echocardiographic videos comparable to senior cardiologists and performed efficiently for heart disease classification on echocardiographic videos from cart-based equipment and handheld device, providing a potential AI-assistant diagnostic tool for the broad application fitting in different types of echocardiographic machines.

## Results

### Deep-learning architecture for the automatic analysis of echocardiographic videos

**Figure 1:**
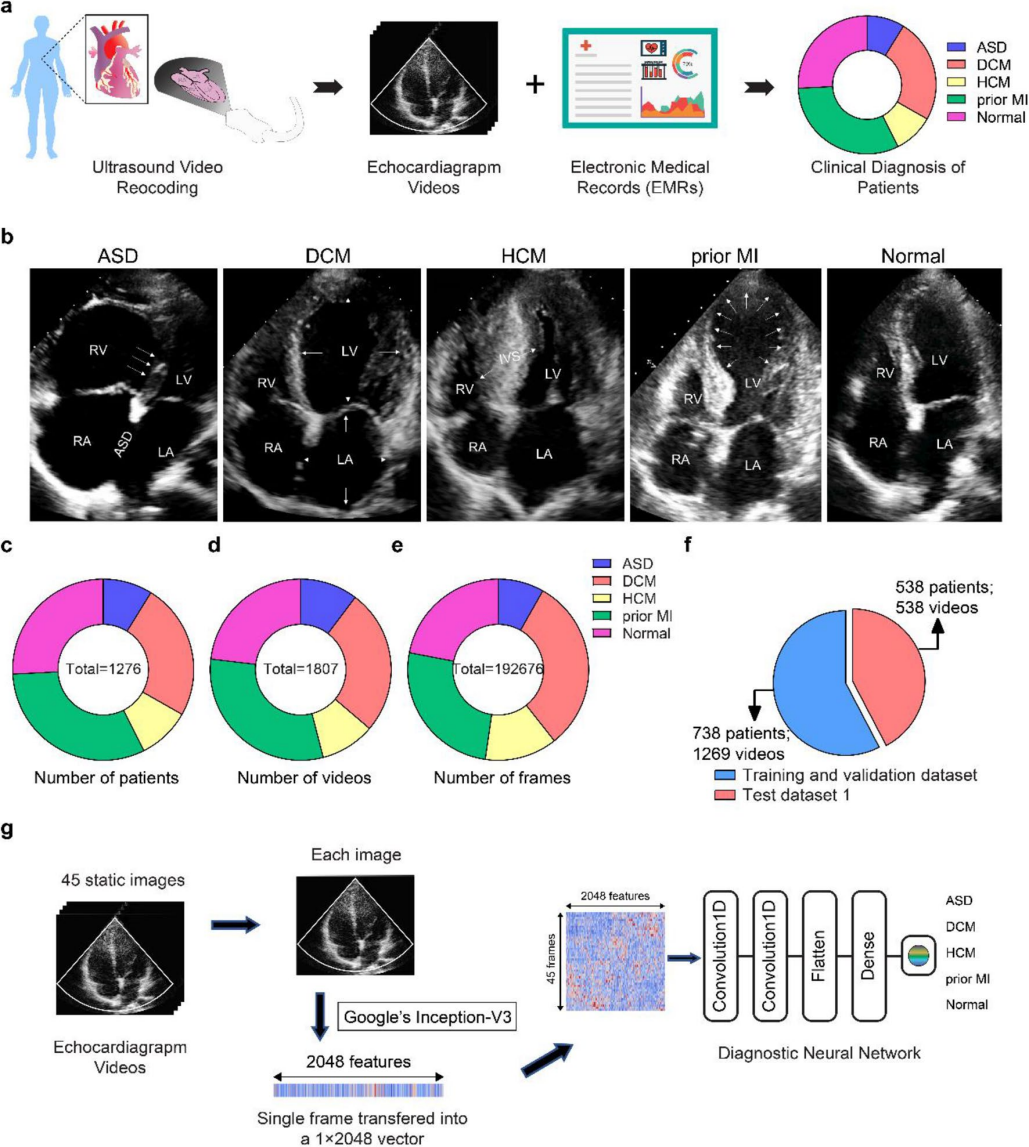
Overview of the study design of AIEchoDx. (**a**) The echocardiogram videos and corresponding electronic medical records were collected for five diagnostic classes, including atrial septal defect (ASD), dilated cardiomyopathy (DCM), hypertrophy cardiomyopathy (HCM), prior myocardial infarction (prior MI), and normal subjects (Normal). (**b**) Examples of apical 4-chamber view (A4c) images of ASD, DCM, HCM, prior MI, and Normal (LV, left ventricle; LA, left atrium; RV, right ventricle; RA, right atrium). The white arrows displayed the morphology changes and disease hallmarks for clinical diagnosis. (**c**–**e**) The distributions of patients (**c**), videos (**d**), and single A4c slice per class (**e**) in the echocardiography dataset to train the deep neural network. (**f**) The distributions of the training, validation dataset, and test dataset 1. (**g**) The architecture of the AIEchoDx framework for analyzing echocardiogram videos.

**Figure 2:**
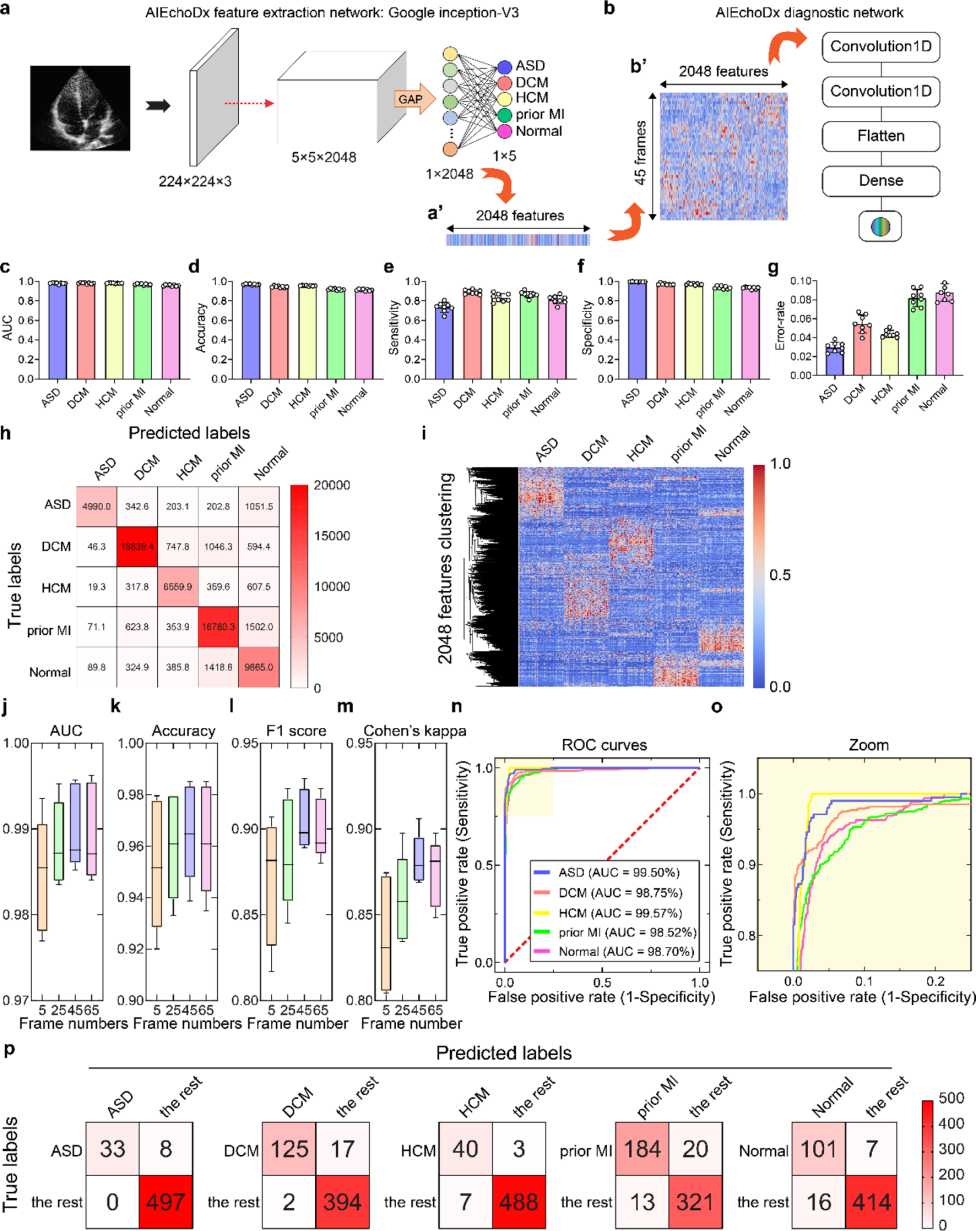
Performance of AIEchoDx. (**a**) Structure of feature extraction network; (**a’**) represents a heatmap of the features of one image extracted from the last hidden layer. (**b**) Structure of the diagnostic network; (**b’**) represents a heatmap of the feature matrix of a 45-frame echo video as an input of the diagnostic network. (**c**–**g**) The AUC (**c**), accuracy (**d**), sensitivity (**e**), specificity (**f**), and error-rate (**g**) values of the eightfold cross-validation results of the feature extraction network. (**h**) Confusion table displays the eightfold cross-validation results of the feature extraction network classified five categories from the test dataset 1. (**i**) The clustered heatmap illustrated 2048 features were extracted from the last hidden layer of the feature extraction network. 50 single frames from each category were selected from 50 patients for plotting the heatmap. (**j**–**m**) The frame number versus the values of matrix accuracy (**j**), F1 score (**k**), Cohen’s kappa (**l**), and AUC (**m**) of cardiac diseases and normal subjects from the diagnostic network. The diagnostic network reading a 45-frame video could achieve the best performance of the prediction in test dataset 1. (**n**,**o**) Receiver operating characteristic (ROC) curves of the 45-frame diagnostic model from test dataset 1 (The AUC values of ASD, DCM, HCM, prior MI, and Normal were 99.50%, 98.75%, 99.57%, 98.52%, and 98.70%, respectively). (**p**) The confusion matrices represent the prediction results of the five classifiers for test dataset 1.

An overview of the study is provided in Fig. 1 and detailed in “Materials and methods”. The AIEchoDx framework was developed as a deep-learning framework for the automatic interpretation of dynamic echocardiographic videos. First, dynamic apical 4-chamber (A4c) echocardiographic videos from ASD, DCM, and prior MI patients and Normal controls were collected (Fig. 1b–d, “Materials and methods”). Each video was split into single frames (Fig. 1e), which was first analyzed by a CNN model (Inception-V3) whose weights were originally optimized with ImageNet^11^. The Inception-V3 model was then fully re-trained to the 5 clinical classes (ASD, DCM, HCM, prior MI, and Normal). After the re-training, each static image was analyzed by the network to obtain a vector of 2048 features extracted from the last hidden layer of the model (Figs. 1g, 2a, and Fig. S2c). Thus, the Inception-V3 network could be considered a “Feature extraction network”.

Then multiple 2048 feature vectors of consecutive frame images were combined to generate a feature matrix to represent a video clip. This feature matrix was then passed through a second, custom-built diagnostic network to arrive at a diagnosis based on information from the entire video clip (Figs. 1g, 2b). The diagnostic network is a four-layer neural network consisting of two 1-dimensional convolutional layers for time-lapse detection, one fully connected internal layer, and one fully connected layer with a sigmoid function (detailed in “Materials and methods”, Figs. 1g, 2b; Fig. S5). We trained 5 independent diagnostic networks to recognize each cardiac condition for binary classification (i.e., the ASD diagnostic network for classifying ASD/non-ASD patients).

### Re-training of inception-V3 for echocardiographic image classification and feature extraction

We obtained 1,807 dynamic A4c echocardiographic videos from the First Medical Center of PLA General Hospital (186, 469, 176, 558, and 418 videos from 113, 310, 121, 406, and 326 patients with ASD, DCM, prior MI, and Normal, respectively, “Materials and methods”, Fig. 1b–d). All the videos were from patients who confirmed the diagnoses of ASD, DCM, HCM, and prior MI through relevant imaging and/or invasive evaluations from December 1, 2013, to June 30, 2019 (angiograms and/or hemodynamic measurements, “Materials and methods”). Electronic medical records and the final clinical characteristics of the patients were provided in Supplementary Table S1a,b.

Each video was split into single frames to form a database of 192,676 single-frame images (Fig. 1e). 58% of the echocardiographic database was randomly selected to train the neural network as the training and validation dataset (124,532 frames from 738 patients). The remaining 42% of the database was used to test the performances of AIEchoDx compared to human experts (68,144 frames from 538 patients as the test dataset) (Fig. 1f and Fig. S2a,b).

To use the Inception-V3 model for analyzing echocardiographic images, we re-trained the model using the single images from the five clinical categories (ASD, DCM, HCM, prior MI, and Normal) with ImageNet as the starting weights^33^, and eightfold cross-validation was done on the cohort (Fig. 2a and Fig. S3a,b). The resulting Inception-V3 echocardiographic image network was then tested using test dataset 1. The cross-validation results showed a strong classification ability (Fig. 2c–g). A comparison of predicted diagnosis versus true clinical diagnosis results is summarized in Fig. 2h with its associated standard deviation matrix presented in Fig. S3c.

In summary, the Inception-V3 echocardiographic image network has an overall good prediction power with error rates for the five clinical categories ranging from 2.97 ± 0.53% to 8.76 ± 0.91% (Fig. 2g; Fig. S3h and Table S3a). The network correctly assigned patients with conditions with sensitivity (true positive) values ranging from 73.49 ± 4.89% to 89.07 ± 2.29% and patients without conditions with specificity (1-false positive rate) values ranging from 93.32 ± 1.10% and 99.63 ± 0.21% (Fig. 2e,f; Fig. S3f–g and Table S3a). In detail, the area under the curves (AUCs) of receiver operating characteristic (ROC) to diagnose ASD, DCM, HCM, prior MI, and Normal were 98.08 ± 0.66%, 98.13 ± 0.65%, 98.22 ± 0.32%, 97.06 ± 0.73%, and 95.89 ± 0.81%, respectively (Fig. 2c; Fig. S3d and Table S3a), while the accuracy and the ROC curves of the five categories are displayed in Fig. 2d and Fig. S3e,i–r. These results indicate that the Inception-V3 echocardiographic image network can successfully extract features representing echocardiographic images.

To explore this further, we examined one of the best-performing models achieved by the resulting Inception-V3 echocardiographic image network. The output of this network’s last hidden layer from each echocardiogram image (2048 features vector) was represented as a column (Fig. 2a’) and the data from 50 randomly selected patients of the five clinical categories were displayed in a clustered heat map (Fig. 2i). The heat map indicated that the output feature from the Inception-V3 echocardiographic image network for each diagnostic category has its fingerprint. Next, we employed principal component analysis (PCA) displaying principal component one versus principal component two to assess the discriminatory capability of these features (Fig. S4). Together, these analyses indicated that the Inception-V3 echocardiographic image network can successfully classify the diagnostic categories by extracting features from single echo images and the 2048 features vector extracted by the network could be used to represent the single echocardiographic image.

### Performance of AIEchoDx

Temporal features are characteristic of videos. Therefore, for analyzing videos, instead of training a diagnostic neural network using separated single-frame images, it is desirable to combine consecutive single frames as a unit. A significant parameter to be determined is the number of consecutive frames to be used as combining all consecutive frames from a video is too cumbersome to use in training. We trained the diagnostic neural network under the different numbers of frames (such as 5, 25, 45, and 65) and found that reliable predictions of cardiac diseases improve as one includes more frames in the analysis of an echocardiographic video, and the reliability reaches a plateau when 45 frames are included. This unit of 45 consecutive static images generally spans at least one cardiac cycle in the apical 4-chamber echocardiographic video (approximately 17 ms for each frame and a total of ~ 765 ms for the cycle) (Fig. 2j–m and Table S3b–e). We, therefore, set the number of frames included in the diagnostic network at 45. For each resulting video clip of 45 frame slices, a 45 × 2048 feature matrix is presented to the diagnostic neural network (Figs. 1g, 2b).

Each dynamic apical 4-chamber echocardiographic video file was divided into smaller video clip files which each contain 45 consecutive static images. Therefore, all the videos from 1,276 patients were converted into 6380 video clips (videos from each patient were converted into five video clips randomly). The 3690 video clips were used for training and validation while the remaining 2690 video clips were for testing. We added a second, multi-layer diagnostic network that uses a continuous matrix derived from one of the best-performed Inception-V3 models (Figs. 1g, 2b; Fig. S5). For each disease category, a dedicated binary neural network was trained in which the video clips in that category were set as one group and the rest were set as the other (“Materials and methods”). This combined two-stage network is named as AIEchoDx.

AIEchoDx has an outstanding performance with improved error rates for the five clinical categories ranging from 1.49 to 6.13% using the test dataset 1 (Table S3d). The AUCs improved to 99.50%, 98.75%, 99.57%, 98.52%, and 98.70% to diagnose ASD, DCM, HCM, prior MI, and Normal, respectively (Fig. 2n,o and Table S3d). Confusion matrices for the assessments of five AIEchoDx classifiers at the probability threshold of 0.5 are shown in Fig. 2p. Thus, AIEchoDx extracting temporal information from cardiac cycle video clips achieves AI predictions with significantly increased sensitivity (from 80.49 to 93.52%) and specificity (from 96.11 to 100.00%) (Table S3d).

To examine the general applicability of AIEchoDx, a “real-world” dataset containing echocardiographic videos and clinical diagnoses of 339 patients from an independent institution was also included in this study (Test dataset 2; Fig. S6a, Table S2c,d). Compared to the performance in test dataset 1, the AUCs in test dataset 2 were only slightly reduced in ASD (97.41% vs 99.50%), HCM (98.75% vs 99.93%), prior MI (98.82% vs 99.57%), and Normal (96.90% vs 98.53%), but slightly increased in DCM (99.83% vs 98.75%) (Fig. S6b); the ROC curves are shown in Fig. S6c–g, respectively.

### Performance of AIEchoDx compared to physicians

**Figure 3:**
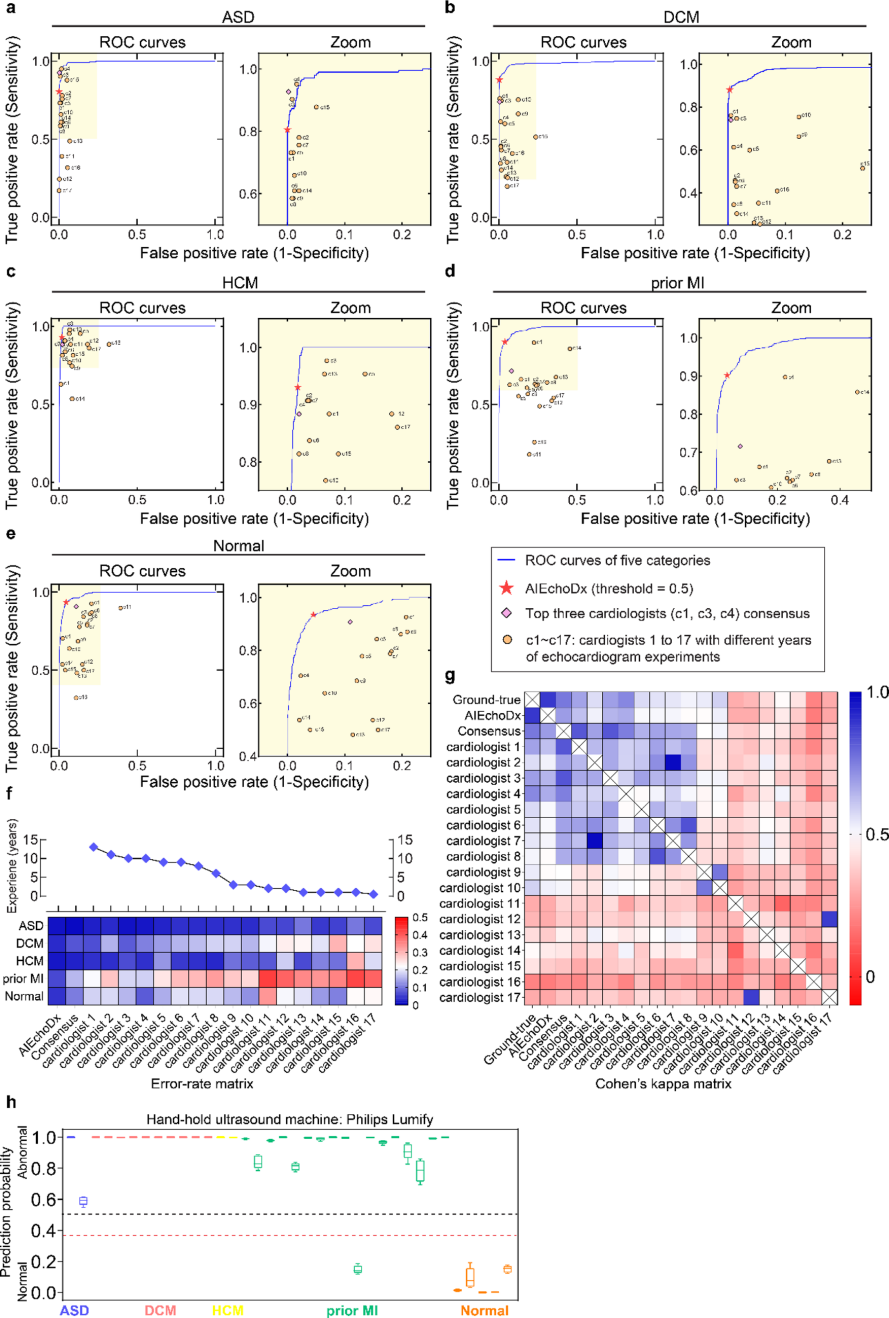
Diagnostic performance comparison between AIEchoDx and physicians. (**a**–**e**) ROC, and zoomed curves for comparing the performance on diagnosing ASD in (**a**), DCM in (**b**), HCM in (**c**), prior MI in (**d**), and Normal in (**e**) among AIEchoDx and seventeen human experts, respectively. The results were calculated from the 538 videos from 538 independent patients in test dataset 1. The red asterisk denoted the performance of AIEchoDx; the filled diamond with purple color denoted the performance of the consensus of three best-performed cardiologists (c1, c3, and c4); while the filled circles with orange color denoted the performance of seventeen physicians (cardiologists 1–17). (**f**) The error rate matrix of AIEchoDx and the seventeen physicians with distinct training experiences of echocardiogram for each of the five categories. The blue diamonds connected with the black line marked the training years for physicians. (**g**) Cohen’s kappa coefficient matrix of AIEchoDx, the expert consensus, and the seventeen physicians. The Cohen’s kappa coefficient matrix of each category has been shown in Fig. S7. (**h**) Prediction probabilities of ASD, DCM, HCM, and prior MI patients and Normal subjects taken by Philips Lumify. The black line represents a threshold of 0.50 and the red line represents a prediction threshold of 0.35 (abnormality detection).

To evaluate the performance of AIEchoDx, we recruited 17 cardiologists with 0.5–13 years of experience in echocardiography diagnosis (designated c1 through c17) from three independent cardiovascular clinical centers (“Materials and methods”). Each physician had not been previously exposed to the clinical data or diagnostic results used in this study and was provided with the full-length apical 4-chamber videos of the 538 cases in the test dataset 1 for evaluation. The evaluation results from each physician for the four specific cardiac diseases and the normal subjects were plotted on the respective ROC curves of the AIEchoDx trained model (circles, Fig. 3a–e, the zoom-in views of the ASD, DCM, HCM, prior MI, and Normal curves are presented at the right). For comparison, the performances of AIEchoDx at the probability threshold of 0.5 (red asterisks) were also plotted on the same ROC curves (Fig. 3a–e). For all clinical categories, we observed that our AIEchoDx model performed significantly better than the cardiologists with less than 10 years of experience (c5–c17) and comparable to the senior cardiologists (c1, c2, c3, and c4 with 13, 11, 10, and 10 years of experience, respectively). The error rates of AIEchoDx and each physician are summarized in Fig. 3f. The detailed values of accuracies, sensitivities, specificities and error rates for the 17 cardiologists are listed in Table S4a–r.

We chose the top three performing physicians (c1, c3, and c4) and plotted the “Expert consensus” values from this group of top performers on the respective ROC curves (diamonds, Fig. 3a–e, “Materials and methods”). Furthermore, we determined the overall agreement in diagnoses among AIEchoDx, the expert consensus, and the individual cardiologists to the actual clinical diagnosis (gold standard) using the Cohen Kappa statistic (Fig. 3g and Table S5a). We observed that the agreement of AIEchoDx was not only significantly higher than the junior cardiologists (0.89 versus 0.21 to 0.59) but also better than the senior cardiologists or the expert consensus (0.89 versus 0.57 to 0.74 and 0.89 versus 0.76, respectively; Table S5a). To provide a comprehensive picture, we created Cohen Kappa coefficient matrices for each of the five diagnostic categories (Fig. S7a–e with confidence intervals displayed in Table S5b–f). The matrix revealed a clear difference in diagnostic accuracy among physicians with different experiences of training. More importantly, AIEchoDx ranked at the top of the Cohen Kappa coefficient matrices (Fig. 3g).

Finally, diagnosis for prior MI is more challenging than other conditions since prior MI patients with subtle or no obvious regional wall motion abnormalities in comparison to significant morphological changes in ASD, DCM, and HCM. Interestingly, AIEchoDx performed significantly better than all physicians including the expert consensus in this section (Fig. 3d).

### Localization of critical areas of cardiovascular diseases

**Figure 4:**
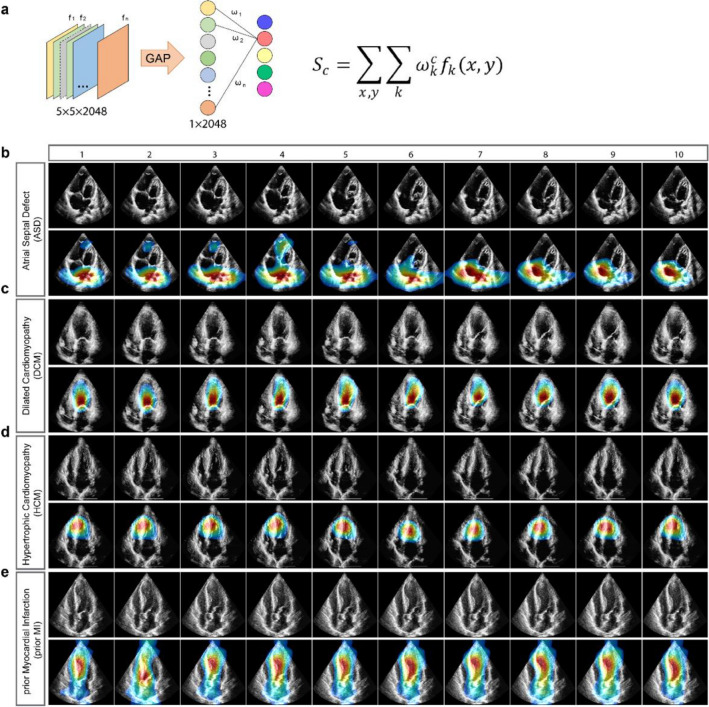
Class activation mapping of four cardiac diseases. (**a**) Class activation maps are generated by mapping back to the previous convolutional layer and the calculation formula listed on the right. (**b**–**e**) Examples of class activation mapping localized the lesion region of ASD in (**b**), DCM in (**c**), HCM in (**d**)**,** and prior MI in (**e**), respectively. The heatmaps of each second row from (**b**–**e**) represented the regions of interest (ROI) automatically identified by class activation mapping.

An important aspect of AI medical image analysis is to be able to identify the regions of interest (ROI) on images; such allows for translation of the abstract feature vectors to the identification of actual sites of abnormalities that are responsible for making a diagnostic prediction which ultimately mimics how physicians make their diagnosis. To achieve this, we utilized class activation mapping (CAM) to make auto-interpretation and localization of ROI^12,13^ (Fig. 4a). For each of the four diseases, we depicted 10 frames from a representative video clip (Fig. 4b–e). The upper row of each panel showed the raw echocardiogram images, while the second row showed the image of the localization heatmaps by CAM. Without any supervised guidance from experts, AIEchoDx identified ROI that was consistent with the respective diagnostic category and that would be used by experts to make diagnoses (Fig. 4b–e). For example, for the diagnosis of ASD, our framework focused on the atria and interatrial septum (Fig. 4b and Supplementary Movie S1). For DCM and prior MI, CAM localized to the left ventricular chamber in both diseases, however, it additionally focused on the akinetic segment of the ventricular wall for prior MI (Fig. 4c,e and Supplementary Movies S2 and S4). Concerning HCM, CAM focused on the interventricular septum (Fig. 4d and Supplementary Movie S3). Thus, as illustrated by these images and videos, the morphologic or structural features of each cardiac disease were readily recognized and localized by AIEchoDx.

### Identifying two distinct phenogroups of DCM

**Figure 5:**
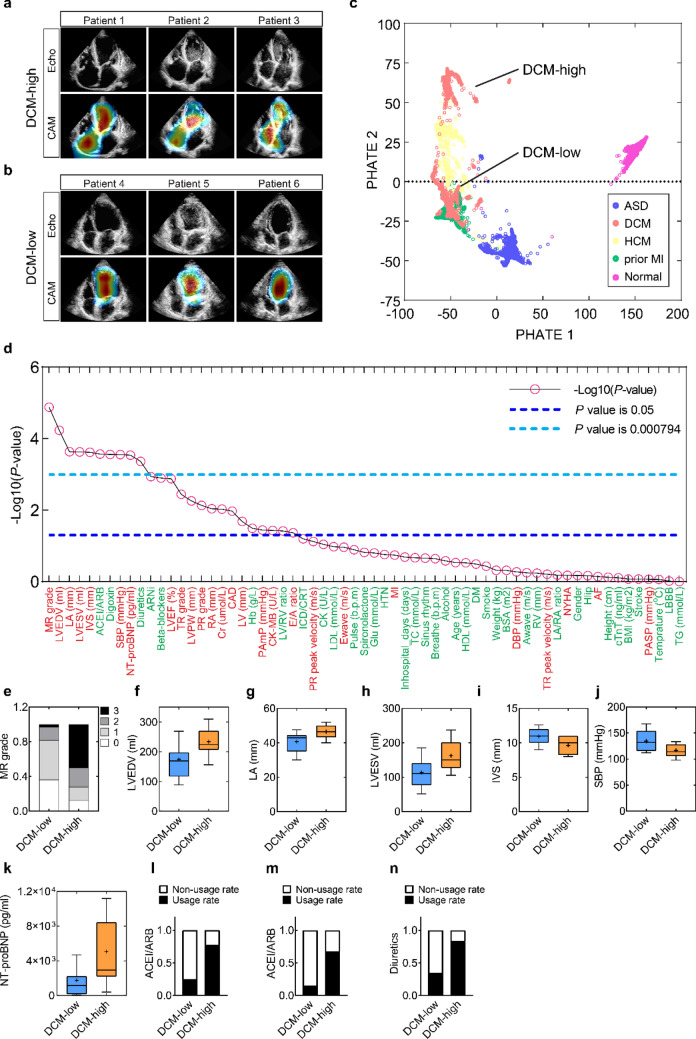
Distinct phenogroups identification. (**a**,**b**) The top row presented six cases with raw echocardiogram images of the DCM-high patients (1–3) and the DCM-low patients (4–6), corresponding with CAM heatmaps in the bottom row. (**c**) PHATE (Potential of Heat-diffusion for Affinity-based Trajectory Embedding) was used as a dimensionality reduction method to visualize structures projected from a 2048-dimensional array generated by the last hidden layer of the Inception-V3 model into a two-dimensional array (PHATE 1 and 2). (**d**) *P*-values of 63 clinical variables in total, sorting from the lowest value to the highest. *P* < 0.05, with a statistical difference, was defined as the threshold plotted in a blue dashed line; *P* < 0.000794 with a statistical difference using the Bonferroni method, was defined as the threshold plotted in a light blue dashed line. Traits commonly related to cardiac conditions are labeled in red, while others are labeled in green. (**e**–**n**) Histograms of 10 clinical characteristics with significant statistical differences using the Bonferroni method.

Interestingly, CAM feature maps revealed two different types of ROI among the echocardiographic videos of DCM patients. Both the right atrium (RA) and left ventricle (LV) are emphasized in one type of DCM patients (DCM-high), while only LV is highlighted in the second (DCM-low) (Fig. 5a,b; Supplementary Movie S5). This raised the possibility that DCM patients could be further divided into two phenogroups as DCM are a primary myocardial disease with highly variable clinical presentations^14,15^.

By applying kernel density estimation with the first and the second principal components, the echocardiogram images of all 166 DCM patients could be represented and smoothed to a 2-D density contour map (Fig. S8e). Unlike the maps of ASD, HCM, prior MI, and Normal, only the map of the DCM patients demonstrated two summits, supporting the possibility of two different phenogroups in DCM patients (Fig. S8). The DCM phenogroups were also separated using a K-mean clustering algorithm (Fig. S9). To further confirm that the DCM group was comprised of relatively distinct phenogroups, we employed a novel affinity-preserving embedding and dimensionality reduction method^16^, called PHATE (Potential of Heat-diffusion for Affinity-based Trajectory Embedding). With this analysis, each point represents the two-dimensional projection of a 2,048-dimensional output of the network’s last hidden layer for each image, which, as was the case for PCA (Fig. S8e), illustrates those images of DCM patients congregate into two distinct phenogroups (Fig. 5c). With concordance of PCA, K-mean clustering, and PHATE analyses, DCM patients could be grouped into two phenogroups (DCM-high and DCM-low).

To further clarify the clinical characteristics and differences of these DCM phenogroups, we examined 63 clinical parameters extracted from the electronic medical record (EMR) system of 33 DCM-high patients and 32 DCM-low patients (Table S6). Overall, 25 characteristics exhibited statistically significant differences between phenogroups at a P level of < 0.05 (Fig. 5d, blue line). Moreover, after applying a Bonferroni correction to account for multiple comparisons (Fig. 5d, light blue line), 10 characteristics remained statistically at P < 0.000794 (where 0.000794 equals 0.05/64) which includes: the mitral regurgitation grade, LV end-diastolic volume, left atrium (LA) dimension, LV end-systolic volume, interventricular septal dimension, prescription of an ACEI or ARB, prescription of digoxin, systolic blood pressure, NT-proBNP and prescription of a diuretic. The differences between these 10 parameters for the two phenogroups are summarized in Fig. 5e–n. Collectively, the findings indicate that the DCM-low phenogroup is characterized by a more mildly dilated cardiomyopathic state (MDCM) with milder systolic dysfunction and less severe clinical manifestations of heart failure as compared to those in the DCM-high phenogroup^17,18^. Trends in other parameters (with slightly higher P values, e.g., LV ejection fraction, tricuspid regurgitation, LV and RA dimensions, and serum creatinine) were also consistent with this conclusion (Fig. S10).

### AIEchoDx for anomaly detection and diseases identification of handheld echocardiography

In addition to being able to make specific diagnoses, another application of AIEchoDx is simply to identify an echocardiographic video as either normal or abnormal; a so-called “anomaly detector”. Anomaly detection is a very practical and important feature for population screening, which often occurs in family and village clinics, ambulances, and emergency room settings where cardiologist experts are not available. For a dichotomous outcome such as this, the goal is to achieve high sensitivity. We, therefore, tested AIEchoDx for this purpose, by setting the probability threshold to 0.35 (predication value for abnormality) and analyzed the two test datasets. For test dataset 1, AIEchoDx identified 417 of 430 (96.97%) patients with abnormal echocardiographic videos, with 10 of 108 (9.25%) false positive detection (Fig. S11a,c). For test dataset 2, AIEchoDx identified 134 of 140 (95.71%) patients with abnormal echocardiographic videos with 34 of 199 (17.09%) false positive detection (Fig. S11b,d).

In recent years, several handheld echocardiography (HHE) devices have become commercially available, including GE Healthcare’s VScan, Philips’ Lumify, and Butterfly Network’s Butterfly IQ^2,3^. The combination of convenience in use, e.g., monitoring images in a cell phone with a portable device, and the price affordability could transform the utilization of ultrasound video in both medical practice and home usage. Medical personnel of non-cardiologist could use HHE for routine physical examination or point-of-care. The devices could also facilitate the learning process for interns, medical and other students. Furthermore, HHE devices could become a common household item similar to a sphygmomanometer for blood pressure. An efficient AI classifier is essential for the realization of such a transformation. To examine the potential utility of AIEchoDx for HHE devices, a new test dataset consisting of apical 4-chamber echocardiographic videos from 36 patients was generated with the Philips’ Lumify device. Test dataset 3 included 2 patients with ASD, 10 with DCM, 2 with HCM, 17 with prior MI, and 5 Normal (Table S2d). AIEchoDx correctly identified nearly all the disease condition cases (30/31) from ASD, DCM, HCM, and prior MI patients (Fig. 3h).

## Discussion

Echocardiography is a powerful imaging tool to screen populations for specific cardiac diseases and to track response to therapy. With technological advances that make acquisition easier and enhance image quality, there is a growing burden and great opportunity for rapid and reliable interpretation of these tests. In the present study, we described a two-step deep-learning framework, AIEchoDx, which can interpret echocardiographic videos by assigning one of five different diagnostic categories: ASD, DCM, HCM, prior MI, and Normal. In AIEchoDx, the first stage employed a retrained Inception-V3 network to extract features and convert each static image into a vector of 2048 features. Then multiple 2048 feature vectors of consecutive frame images were combined to generate a feature matrix to represent a video clip. The second phase of AIEchoDx consisted of a diagnostic neural network. Our analysis showed that we achieved the best performance when the network was trained with 45 frames of a given video, which typically spans a complete heartbeat. This implies that the network relies on information contained within frames at different points in the cardiac cycle, which mimics how physicians interpret these videos. With this approach, we have achieved an overall AUC of 0.99 which corresponded to sensitivities and specificities to make a proper diagnosis comparable to those achieved by senior cardiologists with ten years of experience on images of cart-based equipment. The approach of analyzing echocardiograms by AIEchoDx could be also adapted for characterizing other medical videos.

Interestingly, analyzing the AIEchoDx results by the CAM algorithm identified anatomic regions of interest relevant to the diagnosis. Thus, similar to an echocardiographer’s approach to interpretation, AIEchoDx identified the interatrial septum for ASD, the left ventricular chamber for DCM, the interventricular septum for HCM, and more variable patterns for prior MI as regions of interest. Such information, particularly when displayed graphically on the images, informs clinicians how, in part, the network arrives at a particular diagnosis and can positively influence a clinician’s decision toward a final diagnosis.

Furthermore, through analyzing features learned from echocardiographic images, our AIEchoDx model demonstrated the ability to phenogroup patients with DCM into milder versus more severe states of heart failure, as confirmed by a multitude of clinical characteristics (Fig. 5), which has significant implications when considering population screening by less well-trained clinicians or untrained technicians.

We also noticed that there are differences among the four conditions when comparing the performance between AIEchoDx and physicians and AIEchoDx significantly outperformed physicians for prior MI. Interestingly, diagnosis of a prior MI can be particularly challenging for physicians because global LV function indexed by ejection fraction can be normal and diagnosis may rely on subtle changes in regional wall motion. Furthermore, with large MI there can be LV chamber dilation and global wall motion abnormalities mimicking DCM. It is possible that AIEchoDx tracks consecutive frames of videos and is particularly sensitive to small changes such as regional wall motion, which are difficult to detect by human eyes.

AIEchoDx was trained using images from patients with four specific common cardiac diseases and normal subjects. As such, the goal of this initial effort was to provide proof of concept that AIEchoDx is efficient to analyze multi-frame echocardiographic videos for disease classification, and yields significant improvements in diagnostic accuracy in comparison to AI analysis of static echocardiographic images^19^. Having now established the methodology, AIEchoDx can be further trained with multiple views of echocardiograms from these four common cardiac diseases or using echocardiographic videos of patients with a wider range of diseases including other forms of ASD (e.g., sinus venosus and coronary sinus defects).

Traditional machine learning and advanced deep learning algorithms have been implemented in cardiovascular medicine, in many cases using support vector machines to diagnose acute coronary syndrome to make referral decisions^20^, applying decision trees to predict cardiovascular event risk^21^, employing tensor factorization to subtype congestive heart failure with preserved ejection fraction^22^ and to analyze static echocardiographic images^19,23–27^. The current results achieved with AIEchoDx show how echocardiographic video image analysis enhances the accuracy of disease diagnostic classification, achieving similar results as senior clinicians. Interestingly, AIEchoDx achieved such a performance overcoming the image variations contributed by examiners at different hospitals or body habitus characteristics of different patients. Importantly, AIEchoDx has similar performance for echocardiographic videos from handheld echocardiography devices. Given the complexity of heart structure and function, clinicians require a fairly long period of training to become experts in evaluating echocardiographic videos. The availability of well-trained echocardiographers can be therefore limited in many settings, even in tertiary care centers where quick and accurate diagnoses are required, such as emergency rooms or in hospitals without full-time trained physicians. As technological advances such as handheld echocardiography devices popularize, the potential applications of the automated tool such as AIEchoDx could not only assist physicians in primary and point-of-care but also aid medical practice at home or remote clinics, significantly broadening the application of AI-assistant echocardiography in different echocardiographic machines and medical settings.

## Materials and methods

### Ethics approval

The Institutional Review Board (IRB) and Ethics Committee (EC) of Chinese PLA General Hospital approvals were obtained (No. S2019-319-01). The work was adherent to the tenets of the Declaration of Helsinki. The IRB/EC provided a waiver of informed consent as all echocardiographic data were deidentified and stored in a deidentified database for this study. This study was registered at the Chinese Clinical Trial Registry center on 27/02/2020 (No. ChiCTR2000030278).

### Echocardiographic datasets and clinical diagnoses

#### Image acquisition and processing

This study included echocardiographic images obtained during standard clinical care of patients presenting to Chinese PLA General Hospital between December 1st, 2013, and September 30th, 2018. Images were obtained from ultrasound equipment from several different manufacturers and models including Mindray M9cv with transducer SP5-1s (Mindray, Shenzhen, Guangdong, China), Siemens SC2000 with transducer 4V1c (Siemens, Munich, Germany), Philips iE-elite with transducer S5-1 and X5-1 (Philips, Andover, MA, USA) and Vivid E95 (General Electric, Fairfield, CT, USA).

The echocardiography videos in our institution’s echocardiography electronic records for DICOM images associated with Normal, ASD, HCM, DCM, or prior MI diagnoses were visually inspected for image quality. Image quality was deemed adequate if 3 or more of the standard 6 LV segments (base, mid-wall, apex of free wall, and septum) and RA, right ventricle (RV), and LA could be visualized. In total, 1,807 echocardiography videos from 1276 patients with Normal, ASD, HCM, DCM, or prior MI conditions were included in this study. Importantly, once passing the initial image quality screen, no videos were excluded from the analysis.

#### Image identification and clinical diagnoses

51,676 inpatients’ echocardiographic reports in the Department of Cardiology between December 1st, 2013, and September 30th, 2018 were reviewed for initial inclusion. According to the ICD-11 code (https://icd.who.int/ and Table S1), patients who met the definition in each disease were enrolled (Fig. S1). Two cardiac residents performed this phase and an independent experienced cardiologist was authorized to confirm the paradoxical cases. Meanwhile, for ASD patients, primum and sinus venous ASD, as well as ASD combined with other defects, were excluded (n = 6, 9, 11, respectively) and for pMI, due to undetected inferior and posterior MI in A4c view, 720 cases were also excluded (Fig. S1). After this phase, if any echocardiographic video is incomplete acquisition (one of the four chambers in A4c view couldn’t be visualized) or the absence of the results cardiac catheterization for final confirmative diagnosis was also excluded (Fig. S1). To establish the whole echocardiogram, 326 age and gender-matched normal patients were included (Fig. S1).

The final database (training and validation dataset (#1) and test dataset 1 (#2)) from The First Medical Center of PLA General hospital included a total of 1807 echocardiographic studies for 1276 patients with the following breakdown: 418 echocardiographic studies from 326 normal subjects (Normal); 186 echocardiographic studies from 113 ASDs; 469 echocardiographic studies from 310 DCMs (patients with symptomatic heart failure and normal coronary anatomy); 176 echocardiographic studies from 121 HCMs; and 558 echocardiographic studies from 406 prior MIs with a history of myocardial infarction (MI, whether or not they have heart failure). The test dataset 2 (#3) from The Fourth Medical Center of PLA General hospital included a total of 339 echocardiographic studies for 339 patients with the following breakdown: 199 echocardiographic studies from 199 normal subjects; 12 echocardiographic studies from 12 ASDs; 9 echocardiographic studies from 9 DCMs; 11 echocardiographic studies from 11 HCMs; and 108 echocardiographic studies from 108 prior MIs with a history of myocardial infarction. The test dataset 3 (Lumify) from The First Medical Center of PLA General hospital included a total of 36 echocardiographic studies for 36 patients with the following breakdown: 5 normal subjects; 2 ASDs; 10 DCMs; 2 HCMs; and 17 prior MIs. Each patient contained only one echocardiographic study.

#### Echocardiographic measurements

Dimensions of four chambers, wall thickness, and myocardial systolic function were evaluated according to international guidelines^28^. Specifically, LV volumes and LV ejection fraction were calculated by Simpson’s biplane method. LV volumes were indexed according to body surface area. Trans-mitral E and A wave velocities were measured using pulsed wave Doppler at the level of the mitral leaflet tips. Mitral regurgitation and tricuspid regurgitation were assessed by visualized classification method. All measurements were obtained from the mean of three beats when the patient was in sinus rhythm or from five beats in the presence of atrial fibrillation.

#### Echocardiographic preprocessing

The 1807 echocardiographic videos obtained from the 1276 patients were split into 192,676 single apical 4-chamber images that were used for training and testing the AI algorithm described below. To enhance image contrast, we applied a CLAHE (Contrast Limited Adaptive Histogram Equalization) algorithm to each image (Fig. S12). 124,532 single images belonging to 738 patients (58%) were used to train the neural networks (described below). These patients were randomly divided into eight groups of approximately equal size (Dataset in Table S2a). Images from the first 1/8th of the patients were used as a validation dataset, while images from the remaining seven subgroups were used to train the model. Eightfold cross-validation was achieved to evaluate the performance of the deep learning model (detailed below). 68,144 echocardiographic images from the other 538 patients (42%, Dataset 2 in Table S2d) were used to test the AI algorithms. Multiple videos from individual patients were clustered so that training, validation, and test datasets were comprised of completely disjointed patients.

The 339 echocardiographic videos in test dataset 2 (Table S2d) and the 36 echocardiographic videos in test dataset 3 (Lumify) (Table S2d) were preprocessed in the same manner as described above.

### Network architectures and training protocol

#### Feature extraction network

The first stage of our AIEchoDx model consists of a “Feature extraction network”. It employed the Inception-v3 network in which the parameters were initialized to the best parameter set that was trained on ImageNet competition. The main advantage of this architecture is the use of inception modules which are made of a variety of convolutions having different kernel sizes (1 × 1, 3 × 3, 5 × 5) along with a 3 × 3 max pooling. The initial 7 layers include 5 convolution layers and 2 max-pooling layers and followed by 11 stacks of inception modules. The end of this architecture is combined with a fully connected global average pooling layer and then a final softmax output layer. At the training time, it takes as input part of the single apical 4-chamber image that has been converted from grayscale to RGB and resized to 224 × 224 × 3. The final softmax layer was trained to recognize the five diagnostic classes using the stochastic gradient descent (SGD) function with a learning rate of 0.001 and momentum of 0.9 was used as the optimizer to train the weights. After removing the final layer, the last hidden layer (the second to last layer) with the outputs of a 2048 vector could be used to represent the single echocardiographic image as a feature vector.

#### Diagnostic network

The diagnostic network is a four-layer neural network and consists of two 1-dimensional convolutional layers for time-lapse detection, one fully connected internal layer, and one fully connected sigmoid layer to recognize one of five cardiac classes. To train the diagnostic network, we first split each echocardiographic video into five video clips with the same frame size *n*. We generated four groups in which the frame size *n* was 5, 25, 45, and 60, respectively. This resulted in 3690 video clips from the 738 patients from the training and validation sets. These clips were next converted to 3440 × *n* × 2048 using the well-trained feature extraction network (outputs from the last hidden layer) in which the value *n* is 5, 15, 30, 45, or 60. We trained the diagnostic network with different values of *n* and finally found that with a value of *n* equaling 45, the diagnostic network could achieve the highest accuracy and the lowest error rate. Increased frame numbers were particularly important for the diagnosis of HCM and prior MI (Fig. S10). During the training, and like the feature extraction network, the diagnostic network also utilized an SGD function with a learning rate of 0.001and a momentum of 0.9 was used as the optimizer to train the weights. A cross-entropy loss function was used to measure the performance of the model.

#### Training the entire network

The Inception-v3 network has been fully trained using the single frame training datasets, following the procedure previously described^29,30^. In this study, the parameters from all convolutional layers, the global average pooling layer, and the fully connected classification layer were optimized by ImageNet with no frozen parts. A dropout layer between the global average pooling layer and the final layer with the parameter of 0.5 was performed during the training procedure to reduce the overfitting of the neural network. During the training period, data augmentation was achieved by rotating the images within ± 15° and zooming in and out within 10%. Data normalization was achieved to transform images to a range of 0–1. When there was no decrease in the cross-entropy loss of the validation datasets, the training job was stopped and the model with the best testing score was selected. In the second stage, we removed the final softmax layer and calculated the output feature vector from the global average pooling layer (last hidden layer; *see* Fig. S2) by taking the single ultrasound image as the input. The datasets with the same size of 45 frames in each clip were then converted into the datasets with 45 × 2048 matrices. The diagnostic networks have been trained using the training and validation datasets of 45 × 2048 matrices to classify our specific categories. The models have been trained in two computer setups, including a Yale high-performance computing machine, the Farnam Cluster, with or without four NVIDIA Tesla K80 GPUs, and a Deep learning workstation purchased from Exxact company with an Intel® Xeon® processor E52650 v4, four 32 GB DDR4 2133 MHz LR ECC LRDIMM memories and four NVIDIA GeForce Titan X Pascal 12 GB GDDR5X GPUs with the Ubuntu 18.04 operating system.

#### Cross-validation

When training complex classifiers such as the Inception-V3 model with a high-dimensional image dataset, overfitting the training data is a typical problem. To estimate the performance of the Inception-V3 model, eightfold cross-validation was achieved on the training and validation dataset. The training and validation dataset (Table S2d) contains five categories and 1,269 apical 4-chamber echocardiographic videos from 738 patients. The dataset was first randomly split into eight groups based on 738 patients. The model was trained eight times. At each time, data from one group was used as the validation dataset, and data from the other seven groups were used as the training dataset. The detailed training was described above. The AUCs, ROC curves, sensitivities, specificities, and error rates were calculated, and the results were presented in Fig. 2c–g and Fig. S3d–r. The confusion matrix and its associated standard deviation matrix were presented in Fig. 2h and Fig. S3c, respectively.

#### Comparisons with physician diagnoses

To evaluate the performance of our model and trained physicians, a test dataset of 538 apical 4-chamber echocardiographic videos from 538 patients (test dataset 1; detailed in Table S2b 2#) is completely independent of the training and validation dataset was generated. For AIEchoDx, we first split the echocardiographic video into five 45-frame video clips with overlap between adjacent video clips to make full use of the echocardiographic videos. Then, the predictions derived from each of the five video clips from a given patient were all determined. The final prediction for each patient was taken as the median value of the five predictions. For physicians, the full length of the 538 apical 4-chamber echocardiographic videos was provided for diagnosis. 17 clinicians with different levels of training and experience (one 13-year cardiologist, one 11-year cardiologist, two 10-year cardiologists, two 9-year cardiologists, one 6-year cardiologist, two 3-year cardiologists, two 2-year cardiologists, four 1-year trainees, and one 0.5-year trainee) from multiple cardiovascular institutions were recruited to make the clinical diagnosis solely based on the echocardiograms. The values of error rates, sensitivities, specificities, and AUCs were calculated based on the prediction results of the algorithm and the human physicians. The values of Cohen’s kappa coefficient of total patients or each of five categories in test dataset 1 were calculated based on the gold standard data, the prediction results of the algorithm at a prediction threshold of 0.5, the consensus of the top 3 ‘best performed’ cardiologists (c1, c3, and c4) and the performances of 17 cardiologists.

### Data analysis

#### ROC curves

ROC curves plotted the true positive rate (sensitivity) versus the false-positive rate (1–specificity). ROC curves and confusion matrices were generated by classification probabilities of true clinical diagnosis versus the false and true labels of each test video clip. The results were computed using mathematical functions/operations written by python from the libraries of Numpy, Pandas, Scikit-Learn, and Matplotlib. The graphs were drawn using GraphPad Prism version 7.

#### Expert consensus

The agreement of the consensus was based on the top 3 performing cardiologists (c1, c3, and c4 with 13-, 10-, and 10 years of experience, respectively). For each of the five categories, the positive ones were diagnosed when at least two of the three agreed; while the negative ones were diagnosed when at least two of the three disagreed. The expert consensus diagnosis of each category was independent of the others.

#### Cohen’s Kappa coefficient

Cohen’s Kappa coefficient is a statistic that is used to quantify inter-rater reliability for different items. The values were calculated based on Python from the libraries of Numpy, Pandas, and Scikit-learn. The heatmaps in Fig. 3g and Fig. S6 were drawn using GraphPad Prism version 7. Bootstrapping has been used to evaluate the confidential intervals of Cohen’s Kappa coefficient between two items and listed in Table S5a–f.

#### Class activation mapping

As described by Zhou et al.^10^, we performed a class activation mapping test to identify the localization of key pathologies of different cardiovascular diseases and to decipher the implicit attention of AIEchoDx. Thus, for a given image, let ${f}_{k}\left(x,y\right)$ represent the activation of unit *k* in the last convolutional layer at spatial location (*x*, *y*). We performed a global average pooling between the last convolutional layer and the final softmax class layer. Thus, for a given class *c*, the softmax predicted value could be represented as ${S}_{c}=\sum_{k}{\omega }_{k}^{c}\sum_{x,y}{f}_{k}\left(x,y\right)$, where ${\omega }_{k}^{c}$ is the weight of class *c* for unit *k*. Finally, the ${S}_{c}$ could be calculated by ${\sum }_{x,y}\sum_{k}{\omega }_{k}^{c}{f}_{k}\left(x,y\right)$, where each spatial location (*x*, *y*) could be given by ${M}_{c}\left(x,y\right)=\sum_{k}{\omega }_{k}^{c}{f}_{k}\left(x,y\right)$ for a class *c*^10^. By calculating each ${M}_{c}\left(x,y\right)$, we can obtain the class activation map; and by resizing the class activation map to the size of the input image, we can localize the ROI on each image belonging to one class (c).

#### Principal component analysis

As described before by Minka et al.^31^, we computed principal components using an algorithm written by python from the Scikit-learn library that performs well on datasets with tens of thousands of samples by approximating only the top *n* principal components that explain the most variation, in which *n* is specified in advance. In Fig. 2d, Figs. S3, S6, and S7, we computed the top 10 principal components using a 25,000 × 2048 matrix generated from the features of 25,000 randomly selected single echocardiogram images from the last hidden layer of the Inception-V3 network and plotted the top 2 or 4 components of the figures, respectively. The 25,000 randomly selected single echocardiogram images were selected from the training dataset with every 5000 images in each cardiac condition. After the transformation, we used the K-mean clustering algorithm to analyze the results.

#### PHATE analysis

As described before by Moon et al.^32^, we reduced the dimension of the 25,000 × 2048 feature matrix output from the last hidden layer of the Inception-V3 network to 2 dimensions. We used the python version of PHATE in the Ubuntu 18.04 operating system. After the transformation, we plot the results and found that DCM patients were automatically isolated into the two phenogroups.

#### Statistical analysis

The clinical characteristics of all patients were expressed as mean and standard deviation, median and interquartile range, or counts and percentage, as appropriate. After the normality test and homogeneity test of variance, comparisons between the clinical records of DCM-low and DCM-high patients were made by chi-square test for discrete variables and the analysis of variance (ANOVA) on continuous variables, or by the non-parametric Wilcoxon rank-sum test when necessary. Results are regarded as statistically significant when *P* < 0.05. All calculations were performed by using IBM SPSS version 23.0 for Mac OS.

## Supplementary Information

Supplementary Information 1.

Supplementary Video 1.

Supplementary Video 2.

Supplementary Video 3.

Supplementary Video 4.

Supplementary Video 5.

## Data Availability

Both training and testing echocardiographic datasets are available from the corresponding author upon reasonable and non-commercial requests.

## Code availability

The code is available at https://github.com/superresolution/AIEchoDx.
