# Supplementary material for: A deep learning framework assisted echocardiography with diagnosis, lesion localization, phenogrouping heterogeneous disease, and anomaly detection
Source: Sci Rep. 2023 Jan 2;13:3. doi: 10.1038/s41598-022-27211-w (PMC9807607; doi:10.1038/s41598-022-27211-w)
Supplement: Supplementary file 1 — Supplementary Information 1. [file 41598_2022_27211_MOESM1_ESM.docx]

**Abbreviations**

CVDs = cardiovascular diseases

AI = artificial intelligence

ASD = atrial septal defect

DCM = dilated cardiomyopathy

HCM = hypertrophic cardiomyopathy

prior MI = prior myocardial infarction

CNN = convolutional neural network

AIEchoDx = AI Echocardiogram Diagnosis Network

A4c = apical 4-chamber

AUCs = the area under the curves

ROC = receiver operating characteristic

PCA = principal component analysis

ROI = the regions of interest

CAM = class activation mapping

RA = right atrium

LV = left ventricle

PHATE = Potential of Heat-diffusion for Affinity-based Trajectory Embedding

ACEI = Angiotensin-Converting Enzyme Inhibitors

ARB = Angiotensin Receptor Blockers

NT-proBNP = N-terminal pro brain natriuretic peptide

MDCM = mildly dilated cardiomyopathy

HHE = handheld echocardiography

RV = right ventricle

LA = left atrium

CLAHE = Contrast Limited Adaptive Histogram Equalization

SGD = the stochastic gradient descent

**Figure S1. The flow diagram of datasets of the training, validation, and internal test.** 51676 echocardiographic reports were reviewed for the initial inclusion to build up the training, validation, and internal test dataset. The definition of the four cardiac diseases was based on the ICD-11 code (<https://icd.who.int/> and Table S1). In ASD and prior MI groups, 6 patients of primum ASD, 9 patients of sinus venous ASD, 11 patients combined with other congenital defects, 720 patients with inferior and posterior MI were excluded. Besides, 113 patients with incomplete acquisition in A4c view due to image quality or patient gesture limitation and 296 patients without the definite results of cardiac catheterization were also excluded. Finally, a total of 1,276 patients of ASD (n=113), DCM (n=310), HCM (n=121), and prior MI (n=406) with A4c view video and 326 normal subjects matched age and gender were enrolled in the training, validation, and internal test.

**
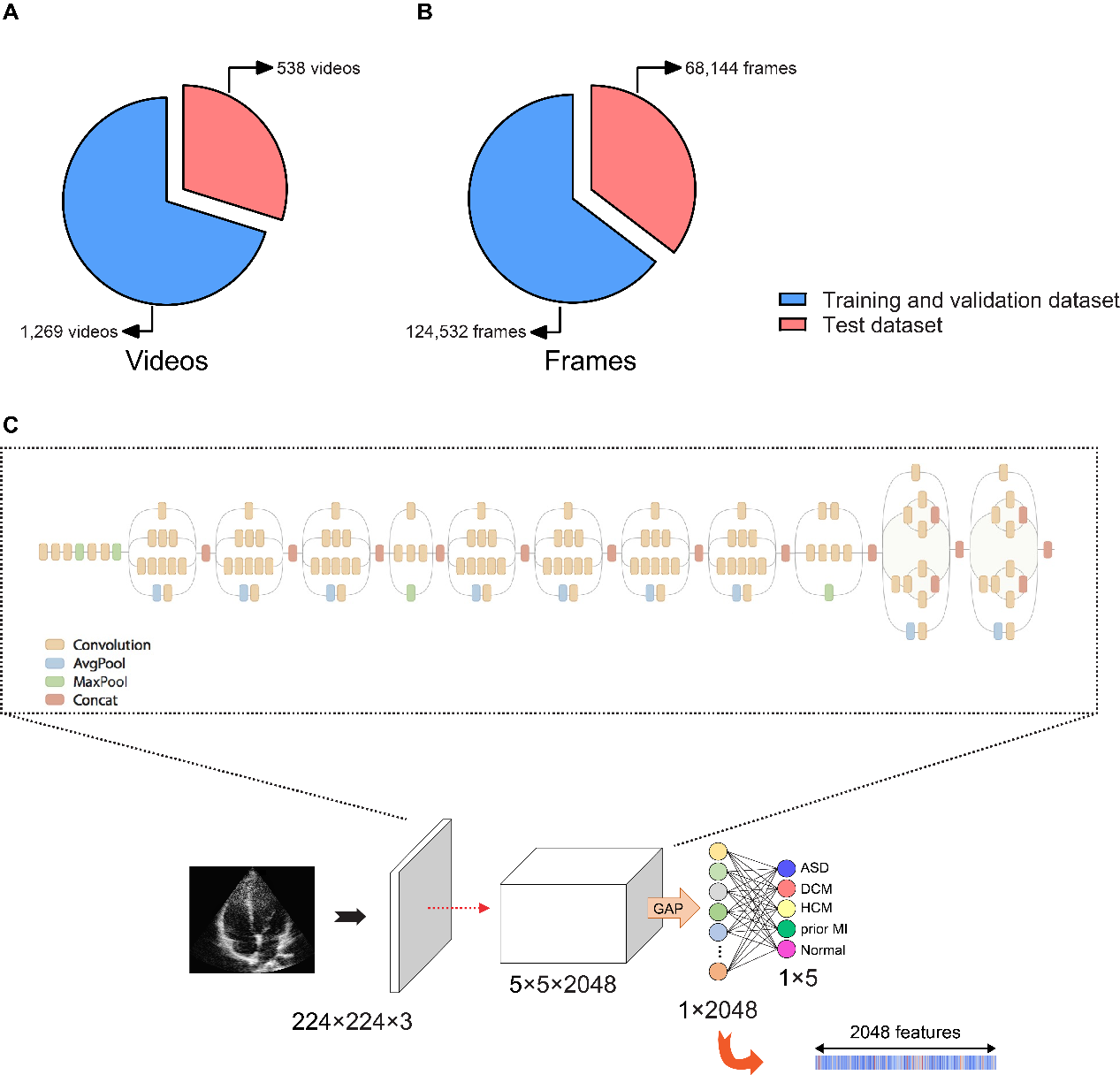
**

**Figure S2. Overview of the dataset and the structure of the feature extraction network, related to Figure 1.** (A) The distribution of total videos in training and validation dataset versus the test dataset in our institution. (B) The distribution of total frames in the training and validation dataset versus the test dataset. (C) The pre-trained Google Inception-V3 network was fine-tuned to detect four classes of heart diseases and normal control based on single images. The Inception-V3 structure graph was captured from <https://ai.googleblog.com/2016/03/train-your-own-image-classifier-with.html>.

**
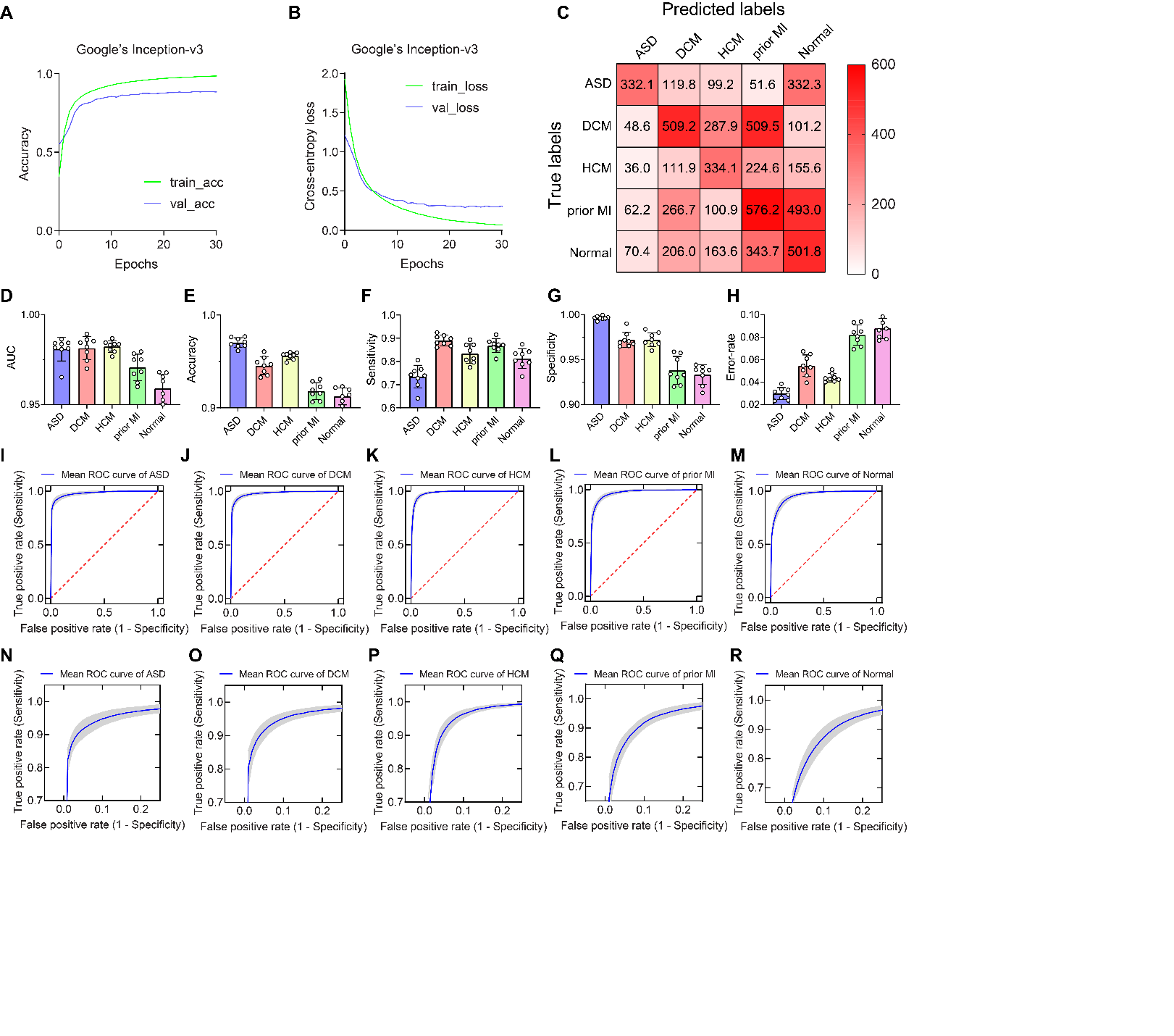
**

**Figure S3. The performance of Inception-V3 network, related to Figure 2.** (A-B) An example of the accuracy and cross-entropy loss of training and validation datasets were plotted as increasing the training epochs. (C) The confusion table displayed the standard deviations of 8-fold cross-validation results of the feature extraction network classified five categories from the test dataset. (D-H) The AUC (D), accuracy (E), sensitivity (F), specificity (G), and error-rate (H) values of the 8-fold cross-validation results of the feature extraction network. (I-M) ROC diagrams of ASD (I), DCM (J), HCM (K), prior MI (L), and Normal (M), respectively. (N-R) Zoomed ROC diagrams of ASD (N), DCM (O), HCM (P), prior MI (Q), and Normal (R), respectively. The grey regions in i-r represent the confidential intervals of ROC curves.


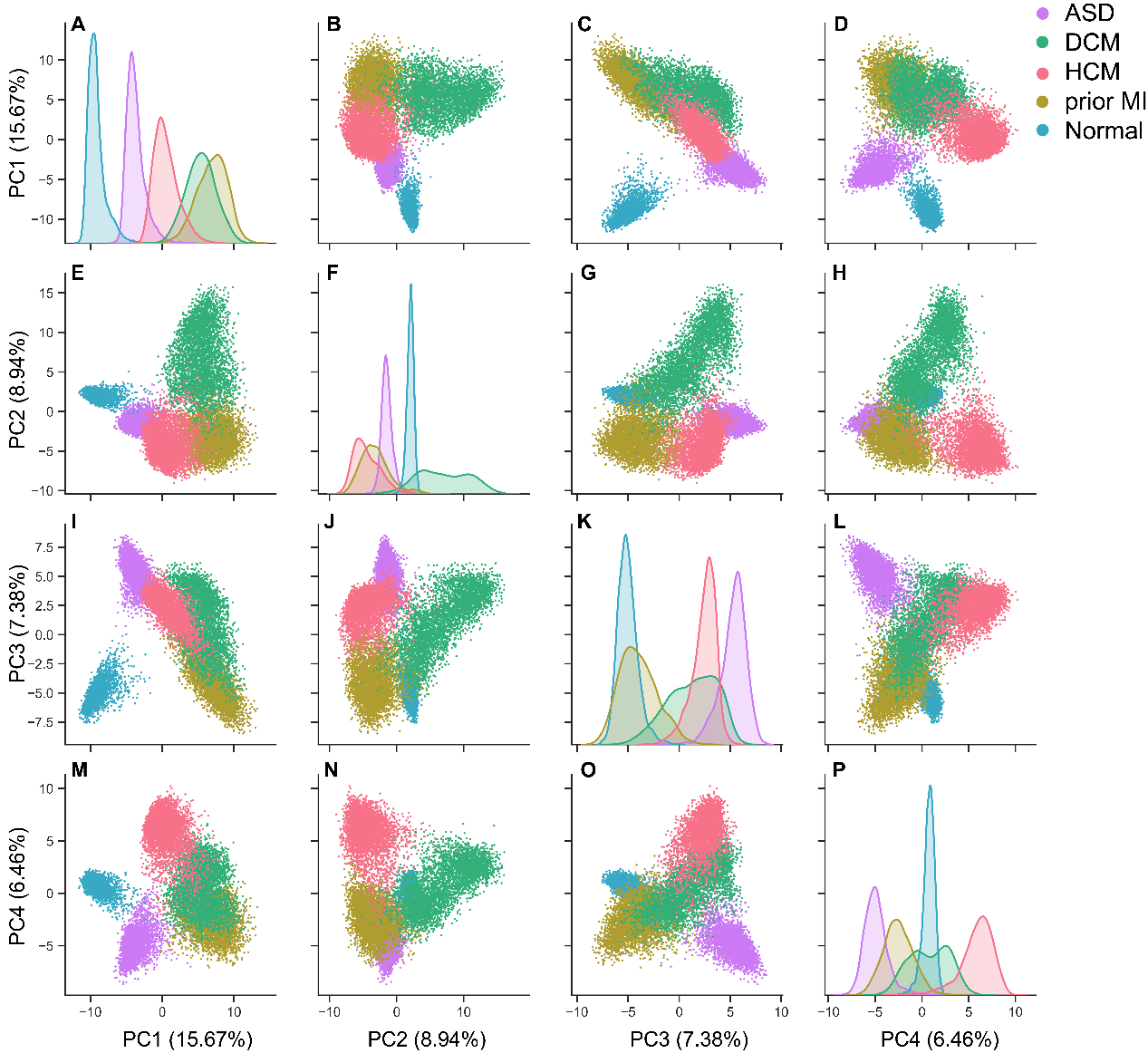


**Figure S4. Principle component analysis (PCA) of the internal features extracted from the last hidden layer, related to Figure 2.** We have utilized PCA to reduce the 2048-dimensional feature matrix into the 4-dimensional data.

**
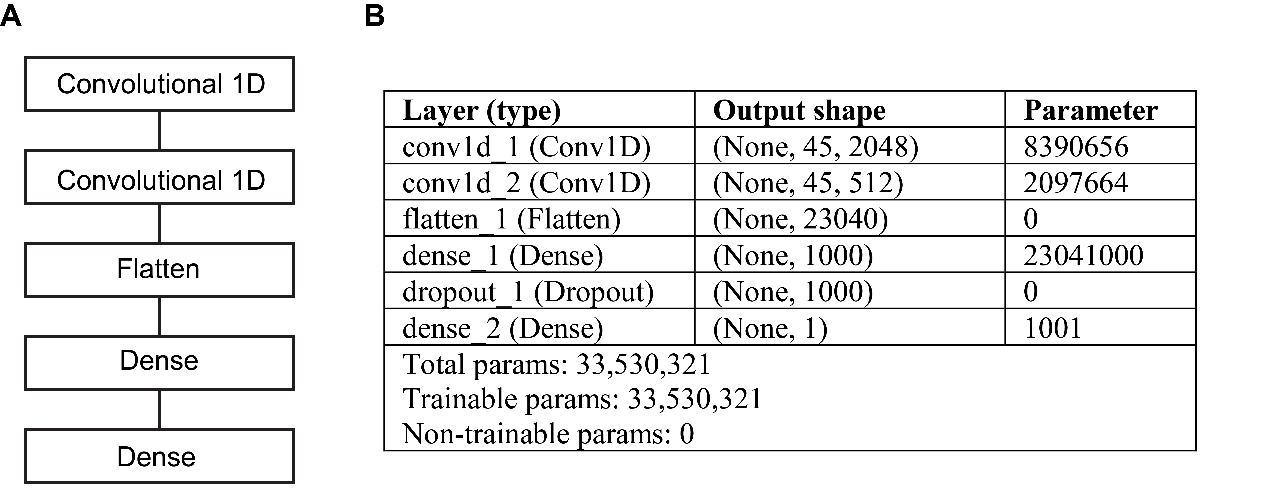
**

**Figure S5. The** **architecture of the diagnostic network and its parameters, related to Figure 2.** (A) The architecture of the diagnostic network; (B) The parameters of each layer of the diagnostic network.


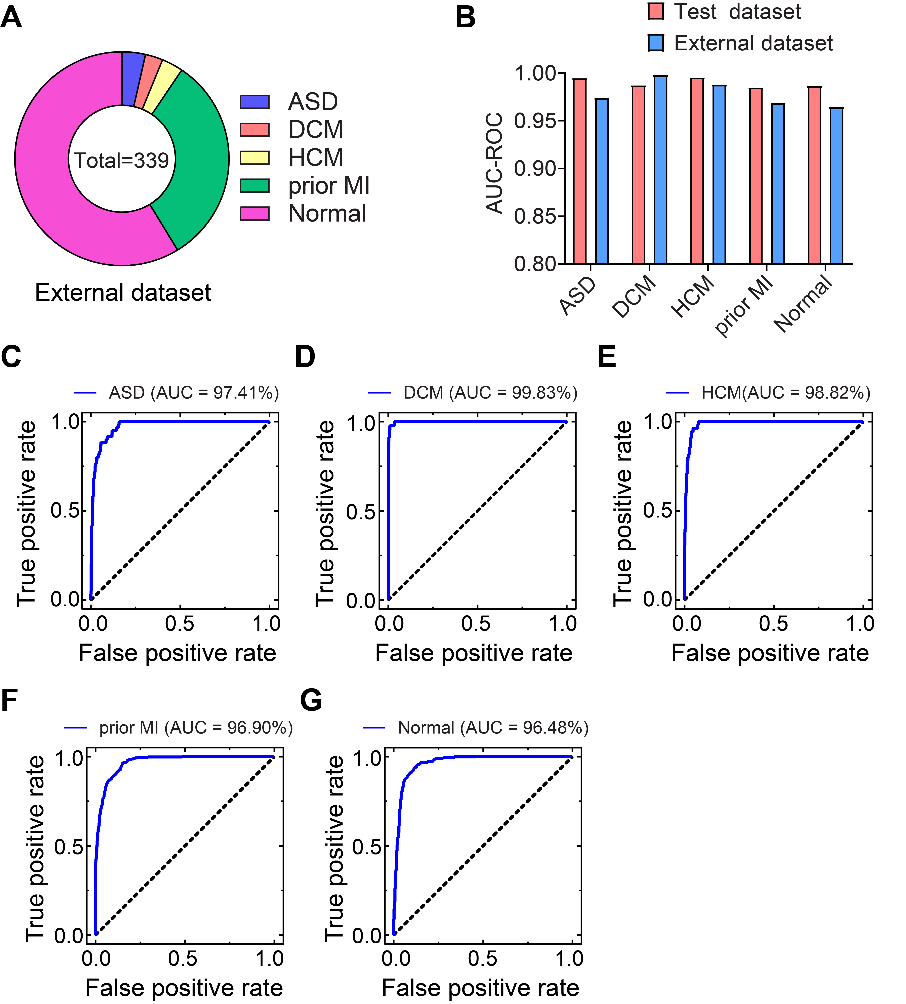


**Figure S6. Performance of the AIEchoDx system on the external dataset, related to Figure 2.** (A) The distributions of patients in echocardiography external dataset. (B) The AUC values of five AIEchoDx classifiers were compared between the test dataset (red) and external dataset (blue). (C-G) ROC curves of AIEchoDx-ASD (C), AIEchoDx-DCM (D), AIEchoDX-HCM (E), AIEchoDx-prior MI (F), and AIEchoDx-Normal (G) in external dataset.

**
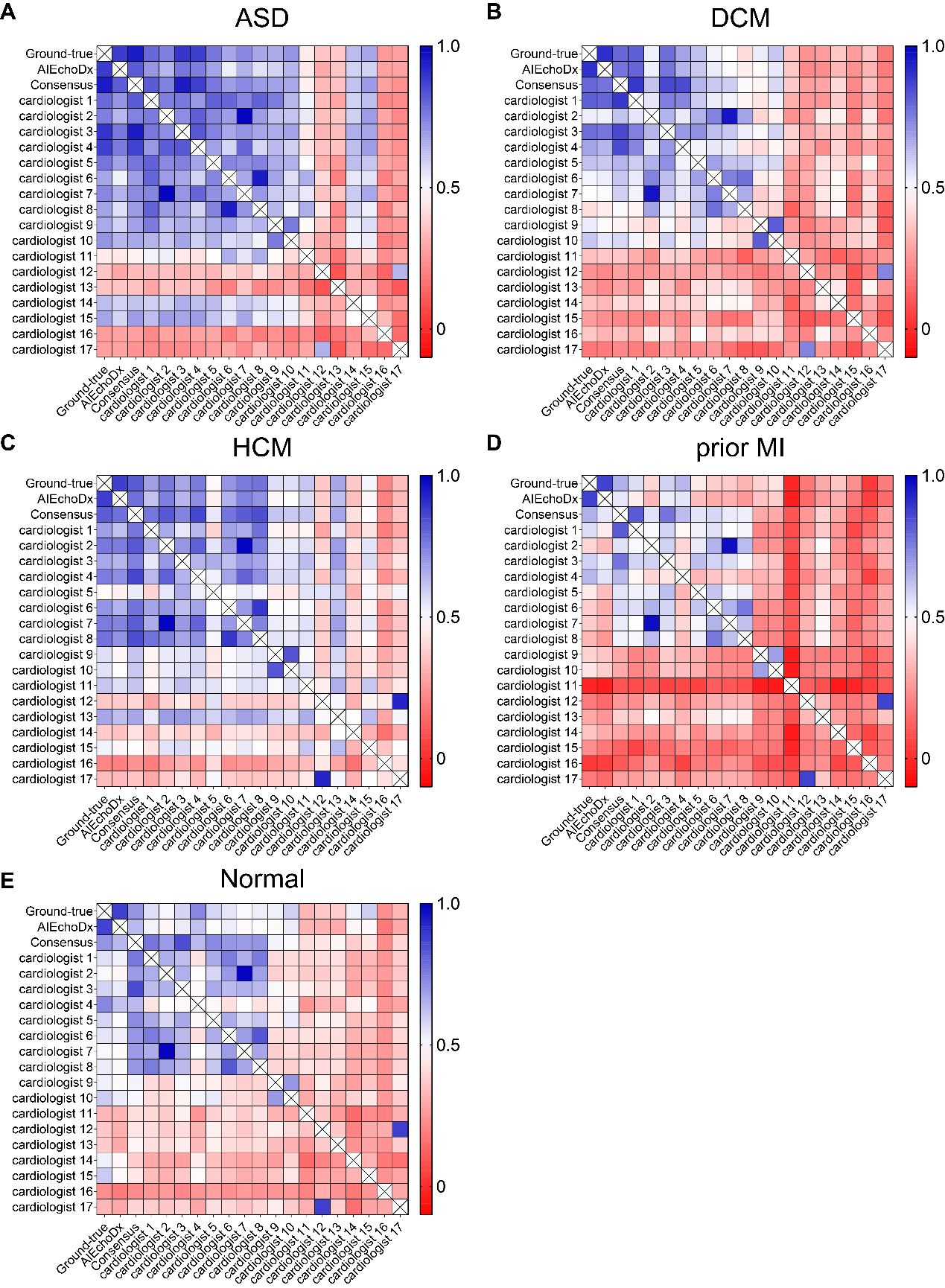
**

**Figure S7.** **The Cohen’s kappa coefficient heatmaps of inter-cardiologists and AIEchoDx model variability when diagnosing each of five categories, related to Figure 3.**


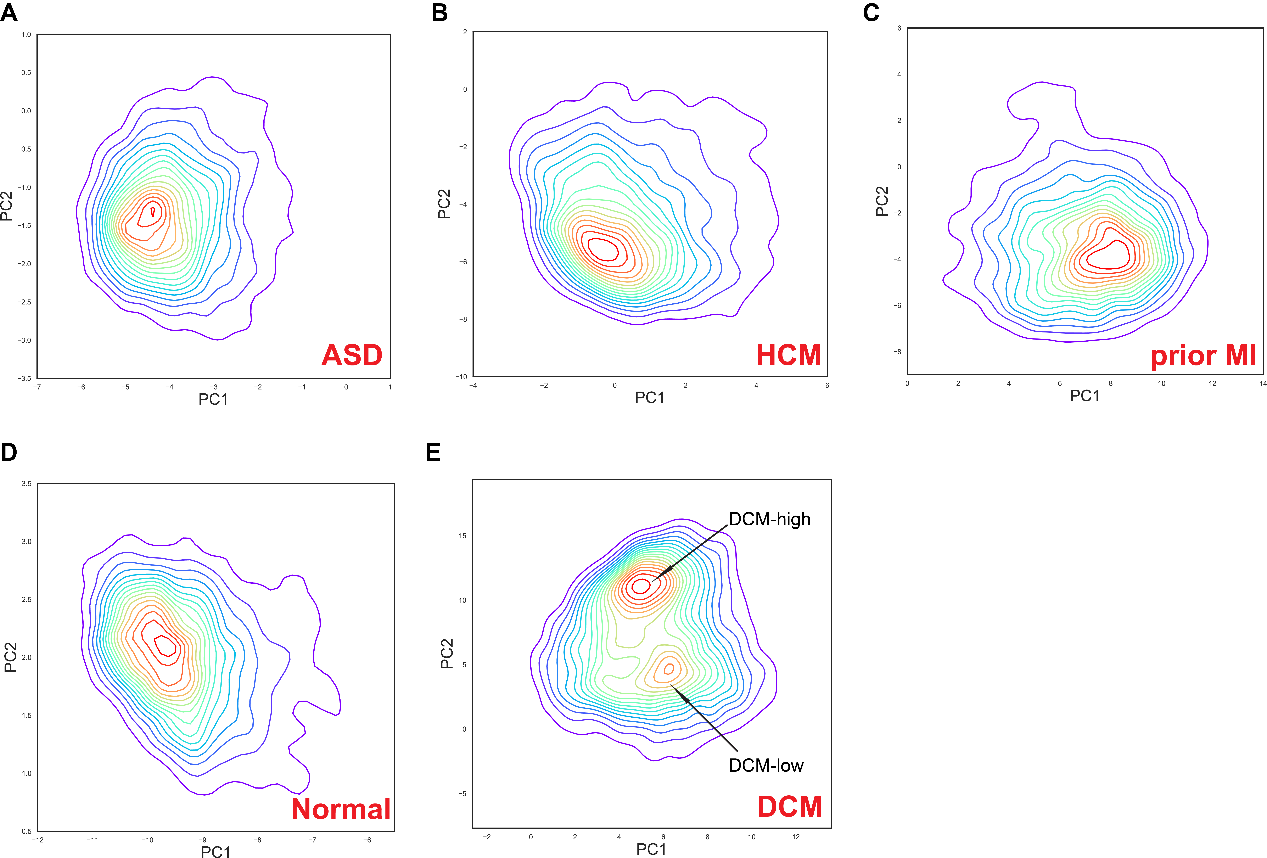


**Figure S8.** **2-D density contour map, related to Figure 5.** (A) 2-D density contour map of ASD training dataset. (B) 2-D density contour map of HCM training dataset. (C) 2-D density contour map of prior MI training dataset. (D) 2-D density contour map of Normal training dataset. (E) 2-D density contour map of DCM training dataset.


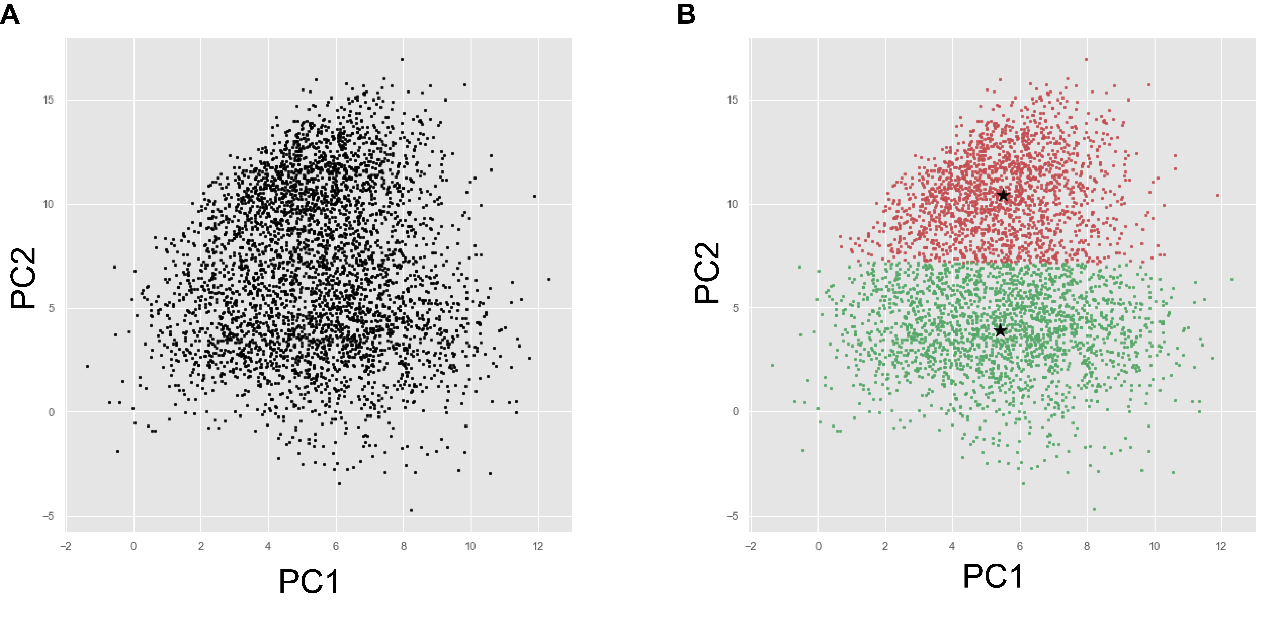


**Figure S9. K-mean clustering was used to find subgroups in DCM patients, related to Figure 5.** (A-B) K-mean clustering was used to identify subgroups from DCM patients’ PCA results.


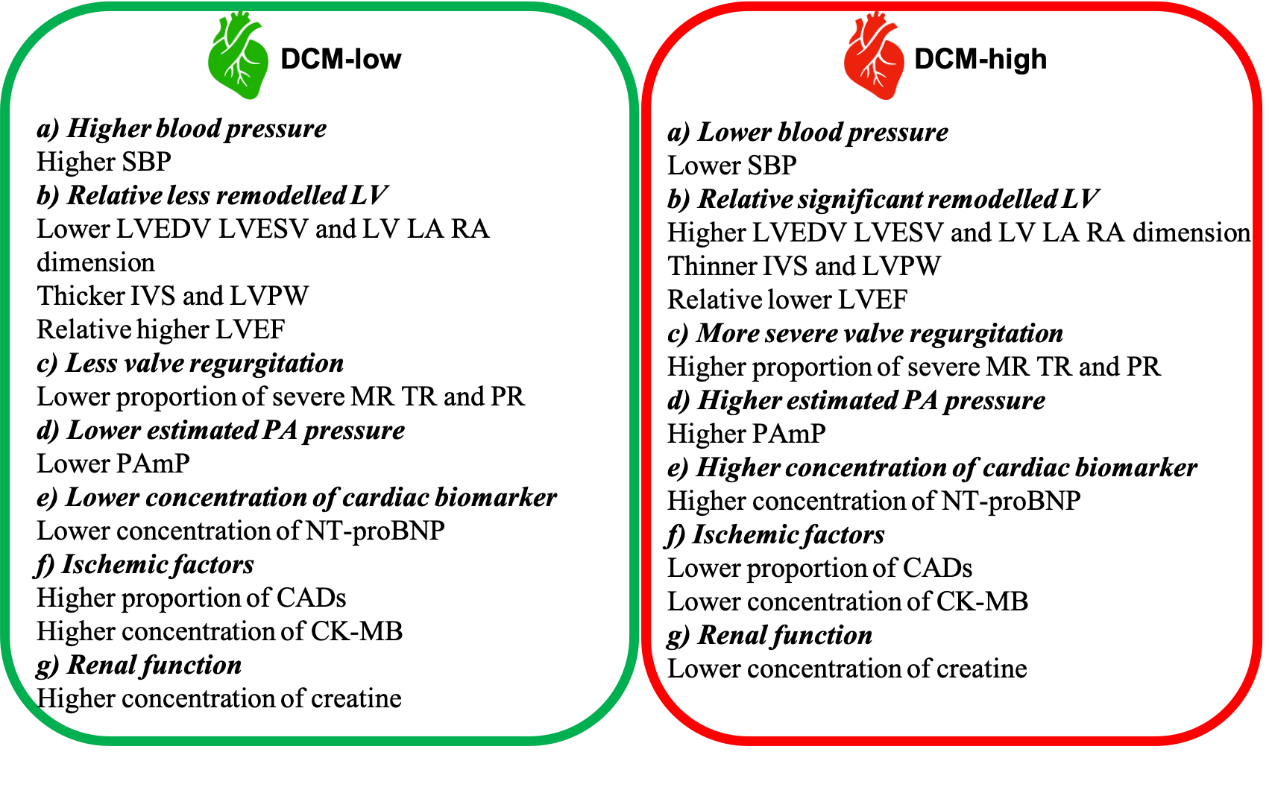


**Figure S10. Typical clinical characteristics of the two phenogroups, DCM-low and DCM-high.** Features listed here demonstrate that the AIEchoDx classifier separated patients into two phenogroups with clinically and physiologically different disease severities.


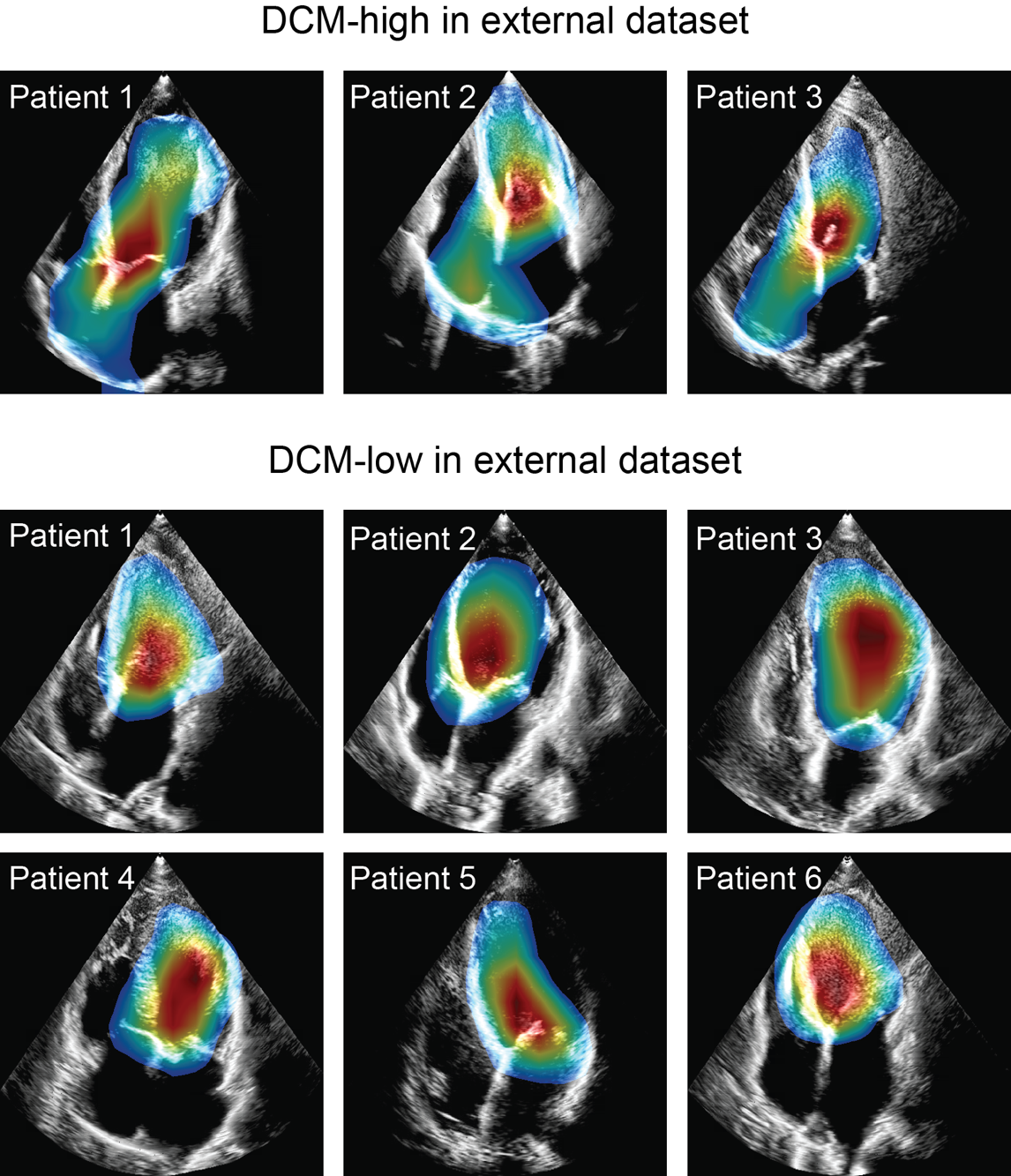


**Figure S11. Class activation mapping (CAM) represented two phenogroups of DCM patients in the external dataset, related to Figure 5.** Three DCM patients from the external dataset were allocated into the DCM-high phenogroup (top row), whereas the remaining six DCM patients from the same cohort belonged to the DCM-low phenogroup (bottom two rows).


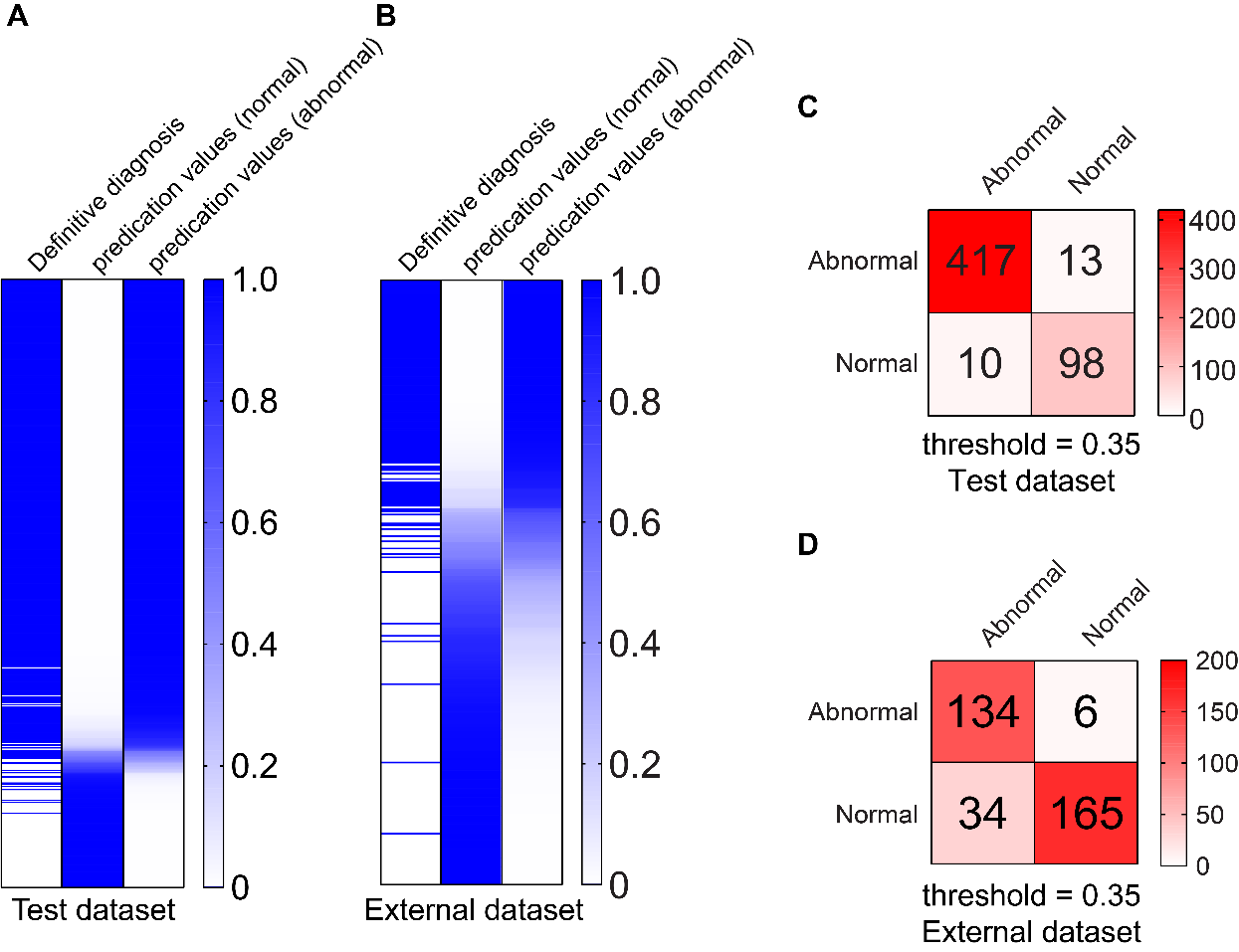


**Figure S12. The distributions of patients in five categories and their associated prediction values, related to Figure S3.** (A) Patients in the test dataset were sorted according to the prediction values of AIEchoDx-Normal from low to high. The left column represents the ground truth distribution of non-normal patients (value = 1) and normal patients (value = 0). The middle column represents the prediction values of all patients (prediction values (normal)). The right column represents 1 – prediction values of all patients (prediction values (abnormal)). (B) Patients in the external dataset were sorted according to the prediction values of AIEchoDx-Normal from low to high. The left column represents the ground truth distribution of non-normal patients (value = 1) and normal patients (value = 0). The middle column represents the prediction values of all patients (prediction values (normal)). The right column represents 1 – prediction values of all patients (prediction values (abnormal)). (C) The confusion table for normal and abnormal ones in the test dataset when a threshold is 0.35 (predication values (abnormal)). (D) The confusion table for normal and abnormal ones in the external dataset when a threshold is 0.35 (predication values (abnormal)).

**
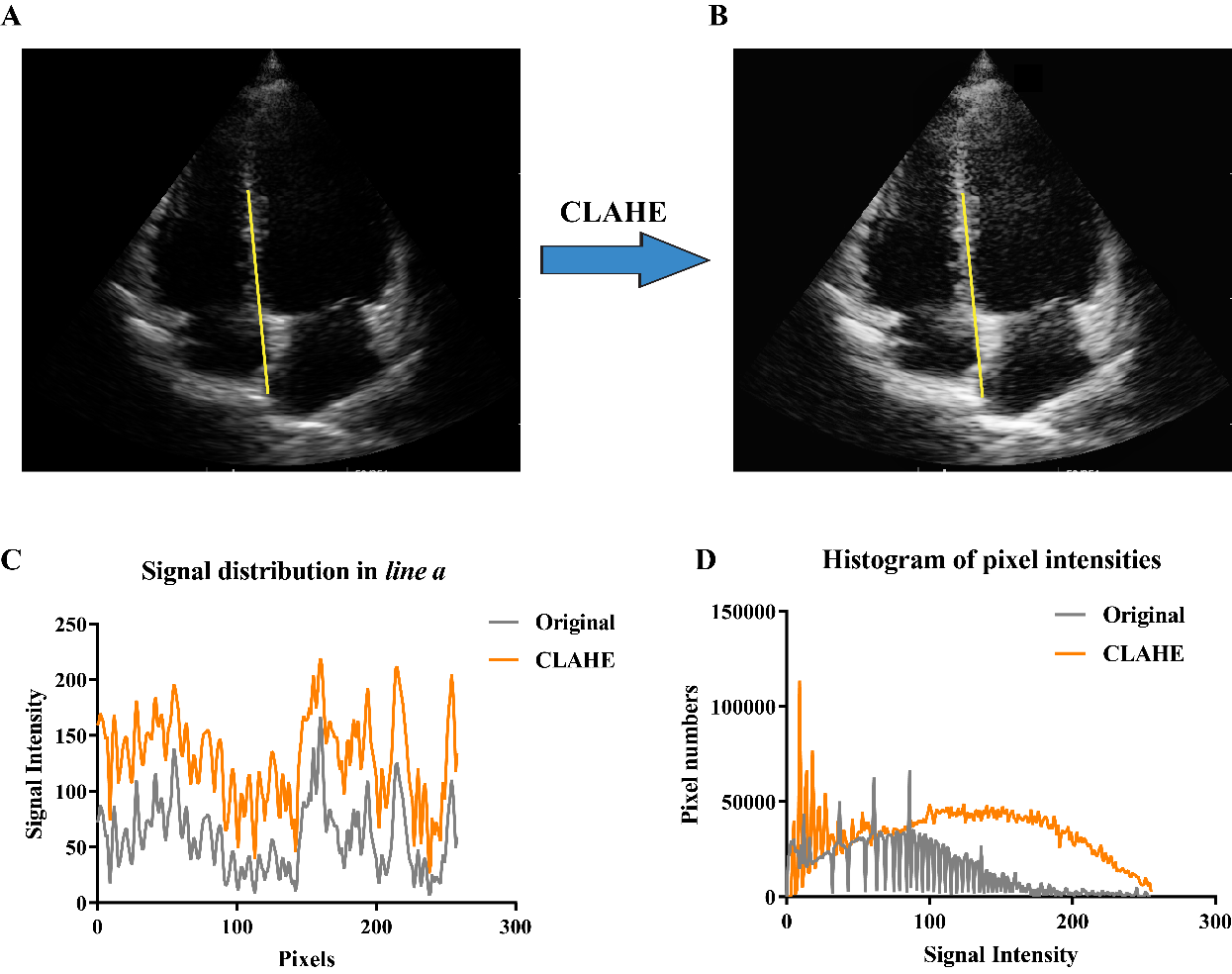
**

**Figure S13. Single echocardiogram frame contrast enhancement by CLAHE (Contrast Limited Adaptive Histogram Equalization) algorithm, related to Discussion and Methods sections.** (A-B) Example of a single image before and after CLAHE treatment. (C) Signal distribution of line a before and after CLAHE treatment. (D) Histogram of pixel intensities distribution.

**Table S1. The definition of the four cardiac diseases**

| **Disease category** | **ICD-11 code** | **ICD-11 name** | **Description** | **Trees and Links** |
| --- | --- | --- | --- | --- |
| ASD | LA8E.1 | Atrial septal defect within oval fossa | A congenital cardiovascular malformation in which there is an interatrial communication confined to the region of the oval fossa (fossa ovalis), most commonly due to a deficiency of the primary atrial septum (septum primum) but deficiency of the septum secundum (superior interatrial fold) may also contribute. Source: ISNPCHD | 20 Developmental anomalies Structural developmental anomalies primarily affecting one body system Structural developmental anomalies of the circulatory system Structural developmental anomaly of heart or great vessels LA8E Congenital anomaly of atrial septum LA8E.1 Atrial septal defect within oval fossa |
| DCM | BC43.0 | Dilated cardiomyopathy | Dilated cardiomyopathy is a myocardial disorder in which there is systolic dysfunction and chamber dilation of one or both ventricles in the absence of a hemodynamic cause that can produce the existent dilation and dysfunction, including physiological (such as sepsis) or anatomic causes with either abnormal loading conditions (such as coarctation of the aorta) or ischemia (such as coronary artery disease or anomalies). Additional information: Physiological and anatomic conditions can affect the dilated cardiomyopathy morphofunctional phenotype. If this morphofunctional phenotype is retained after appropriate intervention, then a dilated cardiomyopathy is established. | [11 Diseases of the circulatory system Diseases of the myocardium or cardiac chambers BC43 Cardiomyopathy BC43.0 Dilated cardiomyopathy](https://icd.who.int/browse11/l-m/en#/http%3a%2f%2fid.who.int%2ficd%2fentity%2f426429380) |
| HCM | BC43.1 | Hypertrophic cardiomyopathy | Hypertrophic cardiomyopathy is the presence of a hypertrophied, non-dilated ventricle in the absence of a hemodynamic cause that is capable of producing the existent magnitude of wall thickening excluding both physiologic hypertrophy secondary to physical activity, and pathologic hypertrophy due to systemic hypertension, aortic valvar stenosis, and coarctation. | 11 Diseases of the circulatory system Diseases of the myocardium or cardiac chambers BC43 Cardiomyopathy BC43.1 Hypertrophic cardiomyopathy |
| pMI | BA50 | Old myocardial infarction | Past myocardial infarction diagnosed by ECG or other special investigation, but currently presenting no symptoms. | 11 Diseases of the circulatory system Ischemic heart diseases Chronic ischemic heart disease BA50 Old myocardial infarction |

**Table S2A. Patient characteristics for each category of heart conditions, related to Figure 1.**

|  | **ASD**  **(n=72)** | **DCM**  **(n=168)** | **HCM**  **(n=78)** | **Normal**  **(n=218)** | **prior MI**  **(n=202)** |
| --- | --- | --- | --- | --- | --- |
| **Demographic** | | | | | |
| Male (%) | 32/72 (44.4) | 120/168 (71.4) | 58/78 (74.3) | 147/218 (67.4) | 168/202 (83.2) |
| Age (years) | 36.1±17.7 | 55.8±14.5 | 57.7±12.5 | 46.5±15.2 | 59.7±16.8 |
| Height (cm) | 159.9±17.1 | 166.5±23.8 | 170.5±7.1 | 139.1±64.0 | 169.0±7.5 |
| Weight (kg) | 57.9±15.8 | 71.0±14.0 | 76.7±13.2 | 57.2±29.2 | 73.1±15.9 |
| SBP (mmHg) | 113.4±21.4 | 83.1±17.9 | 138.5±21.6 | 118.3±33.1 | 125.6±17.8 |
| DBP (mmHg) | 69.1±11.9 | 75.2±13.2 | 81.1±15.4 | 71.1±20.4 | 75.6±9.9 |
| HR (b.p.m) | 79.6±12.7 | 83.1±17.6 | 78.1±13.2 | 77.3±21.7 | 77.7±12.9 |
| **Laboratory tests** | | | | | |
| cTnT (ng/ml) | 0.005±0.005 | 0.070±0.300 | 0.020±0.020 | 0.007±0.040 | 0.820±2.100 |
| NT-proBNP (pg/ml) | 116.1±226.1 | 4431.9±5413.3 | 603.2±1208.5 | 142.8±439.5 | 5126.7±9136.6 |
| Creatinine (μmol/L) | 65.3±14.6 | 92.5±62.4 | 94.1±52.5 | 65.7±30.9 | 103.8±90.5 |
| Glucose (mmol/L) | 5.3±1.6 | 6.1±3.0 | 6.0±1.9 | 5.4±1.8 | 7.5±3.6 |
| **Echocardiographic** | | | | | |
| LVEF (%) | 62.8±4.5 | 31.3±10.3 | 62.4±5.4 | 61.5±3.4 | 44.0±7.9 |
| LVEDV (ml) | 68.8±22.7 | 210.7±73.1 | 95.1±21.6 | 93.2±19.6 | 146.4±165.1 |
| LVESV (ml) | 28.2±17.7 | 149.4±70.3 | 36.3±11.9 | 35.5±8.2 | 70.4±25.1 |
| LV (mm) | 39.5±5.3 | 59.8±8.7 | 45.2±3.7 | 37.1±15.4 | 48.1±8.9 |
| RV (mm) | 39.3±8.9 | 36.9±7.0 | 31.6±3.6 | 31.2±3.3 | 32.3±2.8 |
| LA (mm) | 31.7±5.7 | 44.6±7.3 | 37.1±4.6 | 30.3±13.1 | 37.8±7.3 |
| RA (mm) | 39.0±8.5 | 39.2±7.3 | 33.9±4.0 | 31.3±2.9 | 33.7±4.2 |
| IVS (mm) | 9.3±1.6 | 10.1±1.5 | 16.9±3.8 | 10.1±1.1 | 10.8±1.6 |

Abbreviation: ASD, atrial septal defect; DCM, dilated cardiomyopathy; HCM, hypertrophic cardiomyopathy; prior MI, prior myocardial infarction; HR, heart rate; b.p.m, beats per minute; SBP, systolic blood pressure; DBP, diastolic blood pressure; LVEDV, left ventricular end diastolic volume; LVESV, left ventricular end-systolic volume; LVEF, left ventricular ejection fraction

**Table S2B. The clinical characteristics of the test dataset, related to Figures 1 and 2**

|  | **ASD**  **(n=41)** | **DCM**  **(n=142)** | **HCM**  **(n=43)** | **Normal**  **(n=108)** | **prior MI**  **(n=204)** |
| --- | --- | --- | --- | --- | --- |
| **Demographic** | | | | | |
| Male (%) | 19/41 (46.3) | 111/142 (78.2) | 33/43 (76.7) | 66/108 (61.1) | 171/204 (83.8) |
| Age (years) | 36.1±17.7 | 55.8±14.5 | 57.7±12.5 | 46.5±15.2 | 59.7±16.8 |
| Height (cm) | 159.9±17.1 | 166.5±23.8 | 170.5±7.1 | 139.1±64.0 | 169.0±7.5 |
| Weight (kg) | 57.9±15.8 | 71.0±14.0 | 76.7±13.2 | 57.2±29.2 | 73.1±15.9 |
| SBP (mmHg) | 113.4±21.4 | 83.1±17.9 | 138.5±21.6 | 118.3±33.1 | 125.6±17.8 |
| DBP (mmHg) | 69.1±11.9 | 75.2±13.2 | 81.1±15.4 | 71.1±20.4 | 75.6±9.9 |
| HR (b.p.m) | 79.6±12.7 | 83.1±17.6 | 78.1±13.2 | 77.3±21.7 | 77.7±12.9 |
| **Laboratory tests** | | | | | |
| cTnT (ng/ml) | 0.005±0.005 | 0.070±0.300 | 0.020±0.020 | 0.007±0.040 | 0.820±2.100 |
| NT-proBNP (pg/ml) | 116.1±226.1 | 4431.9±5413.3 | 603.2±1208.5 | 142.8±439.5 | 5126.7±9136.6 |
| Creatinine (μmol/L) | 65.3±14.6 | 92.5±62.4 | 94.1±52.5 | 65.7±30.9 | 103.8±90.5 |
| Glucose (mmol/L) | 5.3±1.6 | 6.1±3.0 | 6.0±1.9 | 5.4±1.8 | 7.5±3.6 |
| **Echocardiographic** | | | | | |
| LVEF (%) | 62.8±4.5 | 31.3±10.3 | 62.4±5.4 | 61.5±3.4 | 44.0±7.9 |
| LVEDV (ml) | 68.8±22.7 | 210.7±73.1 | 95.1±21.6 | 93.2±19.6 | 146.4±165.1 |
| LVESV (ml) | 28.2±17.7 | 149.4±70.3 | 36.3±11.9 | 35.5±8.2 | 70.4±25.1 |
| LV (mm) | 39.5±5.3 | 59.8±8.7 | 45.2±3.7 | 37.1±15.4 | 48.1±8.9 |
| RV (mm) | 39.3±8.9 | 36.9±7.0 | 31.6±3.6 | 31.2±3.3 | 32.3±2.8 |
| LA (mm) | 31.7±5.7 | 44.6±7.3 | 37.1±4.6 | 30.3±13.1 | 37.8±7.3 |
| RA (mm) | 39.0±8.5 | 39.2±7.3 | 33.9±4.0 | 31.3±2.9 | 33.7±4.2 |
| IVS (mm) | 9.3±1.6 | 10.1±1.5 | 16.9±3.8 | 10.1±1.1 | 10.8±1.6 |

Abbreviation: ASD, atrial septal defect; DCM, dilated cardiomyopathy; HCM, hypertrophic cardiomyopathy; prior MI, prior myocardial infarction; HR, heart rate; b.p.m, beats per minute; SBP, systolic blood pressure; DBP, diastolic blood pressure; LVEDV, left ventricular end-diastolic volume; LVESV, left ventricular end-systolic volume; LVEF, left ventricular ejection fraction

**Table S2C. The clinical characteristics of the external testing dataset, related to Figure S6.**

|  | **ASD**  **(n=12)** | **DCM**  **(n=9)** | **HCM**  **(n=11)** | **Normal**  **(n=199)** | **prior MI**  **(n=108)** |
| --- | --- | --- | --- | --- | --- |
| **Demographic** | | | | | |
| Male (%) | 7/12 (58.3) | 7/9 (77.8) | 10/11 (90.9) | 113/199 (56.8) | 89/108 (82.4) |
| Age (years) | 32.5±20.8 | 63.8±12.9 | 55.0±17.6 | 38.7±14.5 | 64.1±18.9 |
| Hight (cm) | 154.1±24.6 | 171.4±6.9 | 170.9±6.4 | 132.3±69.9 | 169.1±7.5 |
| Weight (kg) | 54.2±19.7 | 71.0±13.6 | 72.9±11.8 | 53.9±29.1 | 71.7±11.5 |
| SBP (mmHg) | 99.4±36.2 | 118.1±9.9 | 146.5±17.0 | 116.7±31.5 | 127.5±18.0 |
| DBP (mmHg) | 60.6±23.3 | 72.6±10.6 | 79.5±14.3 | 70.9±20.0 | 73.9±10.1 |
| HR (b.p.m) | 86.9±14.5 | 92.3±17.4 | 84.0±14.2 | 76.2±21.4 | 75.6±13.1 |
| **Laboratory tests** | | | | | |
| cTnT (ng/ml) | 0.002±0.002 | 0.018±0.020 | 0.021±0.010 | NA | 0.500±2.000 |
| NT-proBNP (pg/ml) | 135.5±179.4 | 2681.4±1665.6 | 1142.4±1156.7 | 103.6±375.8 | 3845.9±7528.6 |
| Creatinine (μmol/L) | 57.0±25.6 | 81.8±18.3 | 106.2±87.8 | 61.3±21.7 | 88.1±44.4 |
| Glucose (mmol/L) | 5.2±3.0 | 5.0±0.6 | 5.2±0.7 | 5.1±1.5 | 6.9±2.6 |
| **Echocardiographic** | | | | | |
| LVEF (%) | 63.5±3.3 | 35.9±14.2 | 61.6±6.9 | 61.5±2.6 | 45.8±7.4 |
| LVEDV (ml) | 63.8±21.4 | 186.6±66.7 | 85.5±25.3 | 93.1±20.4 | 151.1±188.6 |
| LVESV (ml) | 23.5±8.3 | 187.4±138.3 | 32.9±12.0 | 35.4±8.2 | 66.8±23.5 |
| LV (mm) | 38.5±6.1 | 60.4±9.1 | 44.5±4.1 | 35.4±17.0 | 47.4±9.4 |
| RV (mm) | 35.9±11.0 | 32.4±8.03 | 30.6±2.8 | 30.9±2.6 | 32.1±2.8 |
| LA (mm) | 31.4±4.9 | 48.6±4.9 | 38.4±5.6 | 29.3±14.2 | 37.8±8.1 |
| RA (mm) | 35.4±10.6 | 37.3±3.6 | 33.1±5.3 | 30.7±2.5 | 33.4±4.0 |
| IVS (mm) | 9.0±2.2 | 10.4±2.4 | 20.0±6.9 | 10.0±1.1 | 11.1±1.6 |

Abbreviation: ASD, atrial septal defect; DCM, dilated cardiomyopathy; HCM, hypertrophic cardiomyopathy; prior MI, prior myocardial infarction; HR, heart rate; b.p.m, beats per minute; SBP, systolic blood pressure; DBP, diastolic blood pressure; LVEDV, left ventricular end diastolic volume; LVESV, left ventricular end-systolic volume; LVEF, left ventricular ejection fraction

**Table S2D. Overview of datasets used for training, validation, and testing of the AIEchoDx systems.**

|  | Dataset | Number of patients | Details | Source |
| --- | --- | --- | --- | --- |
| #1 | Training and validation datasets | 738 | ASD: 72  DCM: 168  HCM: 78  prior MI: 202  Normal: 218 | The First Medical Center of PLA General hospital |
| #2 | Test dataset | 538 | ASD: 41  DCM: 142  HCM: 43  prior MI: 204  Normal: 108 | The First Medical Center of PLA General hospital |
| #3 | External dataset | 339 | ASD: 12  DCM: 9  HCM: 11  prior MI: 108  Normal: 199 | The Fourth Medical Center of PLA General hospital |
| #4 | Lumify’s test dataset | 36 | ASD: 2  DCM: 10  HCM: 2  prior MI: 17  Normal: 5 | The First Medical Center of PLA General hospital |
| #5 | Human results:  Clinicians on A4c ultrasound only | Same as #2 | Cardiologists: 17 | 1) The First Medical Center of PLA General hospital;  2) Beijing Tongren Hospital;  3) The Fourth Medical Center of PLA General hospital; |

**Table S3A. The diagnostic performance of Inception-V3 alone on 8-fold cross validation, related to Figure 2.**

|  | **ASD**  **(n = 15,828)** | **DCM**  **(n = 59,864)** | **HCM**  **(n = 25,131)** | **prior MI**  **(n = 49,719)** | **Normal**  **(n = 42,134)** | **Mean** |
| --- | --- | --- | --- | --- | --- | --- |
| **AUC** | 98.09±0.66% | 98.13±0.65% | 98.22±0.32% | 97.06±0.74% | 95.89±0.81% | 97.48±1.10% |
| **Accuracy** | 97.03±0.53% | 94.54±0.95% | 95.61±0.38% | 91.81±0.90% | 91.23±0.91% | 94.04±2.36% |
| **Sensitivity** | 73.49±4.89% | 89.07±2.29% | 83.42±4.25% | 86.80±2.98% | 81.33±4.22% | 82.82±6.55% |
| **Specificity** | 99.63±0.21% | 97.20±0.84% | 97.20±0.78% | 93.80±1.55% | 93.32±1.10% | 96.23±2.57% |
| **Error Rate** | 2.97±0.53% | 5.46±0.95% | 4.39±0.38% | 8.19±0.90% | 8.77±0.91% | 5.96±2.36% |

Abbreviation: ASD, atrial septal defect; DCM, dilated cardiomyopathy; HCM, hypertrophic cardiomyopathy; prior MI, prior myocardial infarction

**Table S3B. The diagnostic performance of the AIEchoDx system (5 frames), related to Figure 2.**

|  | **ASD**  **(n = 41)** | **DCM**  **(n = 142)** | **HCM**  **(n = 43)** | **prior MI**  **(n = 204)** | **Normal**  **(n = 108)** | **Mean** |
| --- | --- | --- | --- | --- | --- | --- |
| **AUC** | 98.77% | 98.55% | 99.35% | 97.92% | 97.69% | 98.46% |
| **Accuracy** | 97.58% | 95.17% | 97.96% | 92.01% | 93.68% | 95.28% |
| **Sensitivity** | 70.73% | 89.44% | 95.35% | 90.20% | 87.04% | 86.55% |
| **Specificity** | 99.80% | 97.22% | 98.18% | 93.11% | 95.35% | 96.73% |
| **Error Rate** | 2.42% | 4.83% | 2.04% | 7.99% | 6.32% | 4.72% |

Abbreviation: ASD, atrial septal defect; DCM, dilated cardiomyopathy; HCM, hypertrophic cardiomyopathy; prior MI, prior myocardial infarction

**Table S3C. The diagnostic performance of the AIEchoDx system (25 frames), related to Figure 2.**

|  | **ASD**  **(n = 41)** | **DCM**  **(n = 142)** | **HCM**  **(n = 43)** | **prior MI**  **(n = 204)** | **Normal**  **(n = 108)** | **Mean** |
| --- | --- | --- | --- | --- | --- | --- |
| **AUC** | 99.11% | 98.72% | 99.53% | 98.35% | 98.44% | 98.83% |
| **Accuracy** | 97.96% | 96.10% | 97.96% | 93.31% | 94.61% | 95.99% |
| **Sensitivity** | 73.17% | 89.44% | 93.02% | 90.69% | 90.74% | 87.41% |
| **Specificity** | 100.00% | 98.48% | 98.38% | 94.91% | 95.58% | 97.47% |
| **Error Rate** | 2.04% | 3.90% | 2.04% | 6.69% | 5.39% | 4.01% |

Abbreviation: ASD, atrial septal defect; DCM, dilated cardiomyopathy; HCM, hypertrophic cardiomyopathy; prior MI, prior myocardial infarction

**Table S3D. The diagnostic performance of the AIEchoDx system (45 frames), related to Figure 2.**

|  | **ASD**  **(n = 41)** | **DCM**  **(n = 142)** | **HCM**  **(n = 43)** | **prior MI**  **(n = 204)** | **Normal**  **(n = 108)** | **Mean** |
| --- | --- | --- | --- | --- | --- | --- |
| **AUC** | 99.50% | 98.75% | 99.57% | 98.52% | 98.70% | 99.01% |
| **Accuracy** | 98.51% | 96.47% | 98.14% | 93.87% | 95.72% | 96.54% |
| **Sensitivity** | 80.49% | 88.03% | 93.02% | 90.20% | 93.52% | 89.05% |
| **Specificity** | 100.00% | 99.49% | 98.59% | 96.11% | 96.28% | 98.09% |
| **Error Rate** | 1.49% | 3.53% | 1.86% | 6.13% | 4.28% | 3.46% |

Abbreviation: ASD, atrial septal defect; DCM, dilated cardiomyopathy; HCM, hypertrophic cardiomyopathy; prior MI, prior myocardial infarction

**Table S3E. The diagnostic performance of the AIEchoDx system (65 frames), related to Figure 2.**

|  | **ASD**  **(n = 41)** | **DCM**  **(n = 142)** | **HCM**  **(n = 43)** | **prior MI**  **(n = 204)** | **Normal**  **(n = 108)** | **Mean** |
| --- | --- | --- | --- | --- | --- | --- |
| **AUC** | 99.47% | 98.71% | 99.62% | 98.50% | 98.40% | 98.94% |
| **Accuracy** | 98.51% | 96.10% | 98.14% | 93.49% | 94.98% | 96.25% |
| **Sensitivity** | 80.49% | 89.44% | 95.35% | 89.22% | 91.67% | 89.23% |
| **Specificity** | 100.00% | 98.48% | 98.38% | 96.11% | 95.81% | 97.76% |
| **Error Rate** | 1.49% | 3.90% | 1.86% | 6.51% | 5.02% | 3.75% |

Abbreviation: ASD, atrial septal defect; DCM, dilated cardiomyopathy; HCM, hypertrophic cardiomyopathy; prior MI, prior myocardial infarction

**Table S4A. The diagnostic performance of the consensus of top 3 cardiologists, related to Figure 3.**

|  | **ASD**  **(n = 41)** | **DCM**  **(n = 142)** | **HCM**  **(n = 43)** | **prior MI**  **(n = 204)** | **Normal**  **(n = 108)** | **Mean** |
| --- | --- | --- | --- | --- | --- | --- |
| **Accuracy** | 99.26% | 92.75% | 97.21% | 84.20% | 89.41% | 92.57% |
| **Sensitivity** | 92.68% | 73.94% | 88.37% | 71.57% | 90.74% | 83.46% |
| **Specificity** | 99.80% | 99.49% | 97.98% | 91.92% | 89.07% | 95.65% |
| **Error Rate** | 0.74% | 7.25% | 2.79% | 15.80% | 10.59% | 7.43% |

Abbreviation: ASD, atrial septal defect; DCM, dilated cardiomyopathy; HCM, hypertrophic cardiomyopathy; prior MI, prior myocardial infarction

**Table S4B. The diagnostic performance of cardiologist 1 with 13-year experience, related to Figure 3.**

|  | **ASD**  **(n = 41)** | **DCM**  **(n = 142)** | **HCM**  **(n = 43)** | **prior MI**  **(n = 204)** | **Normal**  **(n = 108)** | **Mean** |
| --- | --- | --- | --- | --- | --- | --- |
| **Accuracy** | 97.40% | 93.31% | 95.91% | 78.51% | 81.97% | 89.42% |
| **Sensitivity** | 73.17% | 76.06% | 62.79% | 66.18% | 92.59% | 74.16% |
| **Specificity** | 99.40% | 99.49% | 98.79% | 85.80% | 79.30% | 92.56% |
| **Error Rate** | 2.60% | 6.69% | 4.09% | 21.49% | 18.03% | 10.58% |

Abbreviation: ASD, atrial septal defect; DCM, dilated cardiomyopathy; HCM, hypertrophic cardiomyopathy; prior MI, prior myocardial infarction

**Table S4C. The diagnostic performance of cardiologist 2 with 11-year experience, related to Figure 3.**

|  | **ASD**  **(n = 41)** | **DCM**  **(n = 142)** | **HCM**  **(n = 43)** | **prior MI**  **(n = 204)** | **Normal**  **(n = 108)** | **Mean** |
| --- | --- | --- | --- | --- | --- | --- |
| **Accuracy** | 96.47% | 84.76% | 96.10% | 71.75% | 81.41% | 86.10% |
| **Sensitivity** | 78.05% | 45.77% | 90.70% | 63.24% | 79.63% | 71.48% |
| **Specificity** | 97.99% | 98.74% | 96.57% | 76.95% | 81.86% | 90.42% |
| **Error Rate** | 3.53% | 15.24% | 3.90% | 28.25% | 18.59% | 13.90% |

Abbreviation: ASD, atrial septal defect; DCM, dilated cardiomyopathy; HCM, hypertrophic cardiomyopathy; prior MI, prior myocardial infarction

**Table S4D. The diagnostic performance of cardiologist 3 with 10-year experience, related to Figure 3.**

|  | **ASD**  **(n = 41)** | **DCM**  **(n = 142)** | **HCM**  **(n = 43)** | **prior MI**  **(n = 204)** | **Normal**  **(n = 108)** | **Mean** |
| --- | --- | --- | --- | --- | --- | --- |
| **Accuracy** | 98.51% | 92.19% | 93.49% | 81.60% | 84.39% | 90.04% |
| **Sensitivity** | 90.24% | 74.65% | 97.67% | 62.75% | 84.26% | 81.91% |
| **Specificity** | 99.20% | 98.48% | 93.13% | 93.11% | 84.42% | 93.67% |
| **Error Rate** | 1.49% | 7.81% | 6.51% | 18.40% | 15.61% | 9.96% |

Abbreviation: ASD, atrial septal defect; DCM, dilated cardiomyopathy; HCM, hypertrophic cardiomyopathy; prior MI, prior myocardial infarction

**Table S4E. The diagnostic performance of cardiologist 4 with 10-year experience, related to Figure 3.**

|  | **ASD**  **(n = 41)** | **DCM**  **(n = 142)** | **HCM**  **(n = 43)** | **prior MI**  **(n = 204)** | **Normal**  **(n = 108)** | **Mean** |
| --- | --- | --- | --- | --- | --- | --- |
| **Accuracy** | 98.14% | 89.03% | 95.91% | 82.16% | 92.19% | 91.49% |
| **Sensitivity** | 95.12% | 61.27% | 90.70% | 89.71% | 70.37% | 81.43% |
| **Specificity** | 98.39% | 98.99% | 96.36% | 77.54% | 97.67% | 93.79% |
| **Error Rate** | 1.86% | 10.97% | 4.09% | 17.84% | 7.81% | 8.51% |

Abbreviation: ASD, atrial septal defect; DCM, dilated cardiomyopathy; HCM, hypertrophic cardiomyopathy; prior MI, prior myocardial infarction

**Table S4F. The diagnostic performance of cardiologist 5 with 9-year experience, related to Figure 3.**

|  | **ASD**  **(n = 41)** | **DCM**  **(n = 142)** | **HCM**  **(n = 43)** | **prior MI**  **(n = 204)** | **Normal**  **(n = 108)** | **Mean** |
| --- | --- | --- | --- | --- | --- | --- |
| **Accuracy** | 97.03% | 86.62% | 87.17% | 75.28% | 85.13% | 86.25% |
| **Sensitivity** | 73.17% | 59.86% | 95.35% | 55.39% | 77.78% | 72.31% |
| **Specificity** | 98.99% | 96.21% | 86.46% | 87.43% | 86.98% | 91.21% |
| **Error Rate** | 2.97% | 13.38% | 12.83% | 24.72% | 14.87% | 13.75% |

Abbreviation: ASD, atrial septal defect; DCM, dilated cardiomyopathy; HCM, hypertrophic cardiomyopathy; prior MI, prior myocardial infarction

**Table S4G. The diagnostic performance of cardiologist 6 with 9-year experience, related to Figure 3.**

|  | **ASD**  **(n = 41)** | **DCM**  **(n = 142)** | **HCM**  **(n = 43)** | **prior MI**  **(n = 204)** | **Normal**  **(n = 108)** | **Mean** |
| --- | --- | --- | --- | --- | --- | --- |
| **Accuracy** | 95.91% | 84.57% | 95.17% | 70.82% | 80.66% | 85.43% |
| **Sensitivity** | 60.98% | 45.07% | 83.72% | 62.25% | 87.04% | 67.81% |
| **Specificity** | 98.79% | 98.74% | 96.16% | 76.05% | 79.09% | 89.77% |
| **Error Rate** | 4.09% | 15.43% | 4.83% | 29.18% | 19.34% | 14.57% |

Abbreviation: ASD, atrial septal defect; DCM, dilated cardiomyopathy; HCM, hypertrophic cardiomyopathy; prior MI, prior myocardial infarction

**Table S4H. The diagnostic performance of cardiologist 7 with 8-year experience, related to Figure 3.**

|  | **ASD**  **(n = 41)** | **DCM**  **(n = 142)** | **HCM**  **(n = 43)** | **prior MI**  **(n = 204)** | **Normal**  **(n = 108)** | **Mean** |
| --- | --- | --- | --- | --- | --- | --- |
| **Accuracy** | 96.28% | 83.83% | 95.91% | 70.45% | 81.41% | 85.58% |
| **Sensitivity** | 75.61% | 42.96% | 90.70% | 62.75% | 78.70% | 70.14% |
| **Specificity** | 97.99% | 98.48% | 96.36% | 75.15% | 82.09% | 90.02% |
| **Error Rate** | 3.72% | 16.17% | 4.09% | 29.55% | 18.59% | 14.42% |

Abbreviation: ASD, atrial septal defect; DCM, dilated cardiomyopathy; HCM, hypertrophic cardiomyopathy; prior MI, prior myocardial infarction

**Table S4I. The diagnostic performance of cardiologist 8 with 6-year experience, related to Figure 3.**

|  | **ASD**  **(n = 41)** | **DCM**  **(n = 142)** | **HCM**  **(n = 43)** | **prior MI**  **(n = 204)** | **Normal**  **(n = 108)** | **Mean** |
| --- | --- | --- | --- | --- | --- | --- |
| **Accuracy** | 96.10% | 81.97% | 96.65% | 67.29% | 81.41% | 84.68% |
| **Sensitivity** | 58.54% | 34.51% | 81.40% | 64.22% | 86.11% | 64.95% |
| **Specificity** | 99.20% | 98.99% | 97.98% | 69.16% | 80.23% | 89.11% |
| **Error Rate** | 3.90% | 18.03% | 3.35% | 32.71% | 18.59% | 15.32% |

Abbreviation: ASD, atrial septal defect; DCM, dilated cardiomyopathy; HCM, hypertrophic cardiomyopathy; prior MI, prior myocardial infarction

**Table S4J. The diagnostic performance of cardiologist 9 with 3-year experience, related to Figure 3.**

|  | **ASD**  **(n = 41)** | **DCM**  **(n = 142)** | **HCM**  **(n = 43)** | **prior MI**  **(n = 204)** | **Normal**  **(n = 108)** | **Mean** |
| --- | --- | --- | --- | --- | --- | --- |
| **Accuracy** | 95.91% | 81.97% | 90.35% | 72.12% | 84.01% | 84.87% |
| **Sensitivity** | 58.54% | 66.20% | 74.42% | 56.86% | 68.52% | 64.91% |
| **Specificity** | 98.99% | 87.63% | 91.70% | 81.44% | 87.91% | 89.53% |
| **Error Rate** | 4.09% | 18.03% | 9.65% | 27.88% | 15.99% | 15.13% |

Abbreviation: ASD, atrial septal defect; DCM, dilated cardiomyopathy; HCM, hypertrophic cardiomyopathy; prior MI, prior myocardial infarction

**Table S4K. The diagnostic performance of cardiologist 10 with 3-year experience, related to Figure 3.**

|  | **ASD**  **(n = 41)** | **DCM**  **(n = 142)** | **HCM**  **(n = 43)** | **prior MI**  **(n = 204)** | **Normal**  **(n = 108)** | **Mean** |
| --- | --- | --- | --- | --- | --- | --- |
| **Accuracy** | 96.28% | 84.39% | 92.01% | 73.98% | 87.55% | 86.84% |
| **Sensitivity** | 65.85% | 75.35% | 76.74% | 60.78% | 63.89% | 68.52% |
| **Specificity** | 98.79% | 87.63% | 93.33% | 82.04% | 93.49% | 91.06% |
| **Error Rate** | 3.72% | 15.61% | 7.99% | 26.02% | 12.45% | 13.16% |

Abbreviation: ASD, atrial septal defect; DCM, dilated cardiomyopathy; HCM, hypertrophic cardiomyopathy; prior MI, prior myocardial infarction

**Table S4L. The diagnostic performance of cardiologist 11 with 2-year experience, related to Figure 3.**

|  | **ASD**  **(n = 41)** | **DCM**  **(n = 142)** | **HCM**  **(n = 43)** | **prior MI**  **(n = 204)** | **Normal**  **(n = 108)** | **Mean** |
| --- | --- | --- | --- | --- | --- | --- |
| **Accuracy** | 93.68% | 79.00% | 92.38% | 56.88% | 66.54% | 77.70% |
| **Sensitivity** | 39.02% | 35.21% | 88.37% | 18.14% | 89.81% | 54.11% |
| **Specificity** | 98.19% | 94.70% | 92.73% | 80.54% | 60.70% | 85.37% |
| **Error Rate** | 6.32% | 21.00% | 7.62% | 43.12% | 33.46% | 22.30% |

Abbreviation: ASD, atrial septal defect; DCM, dilated cardiomyopathy; HCM, hypertrophic cardiomyopathy; prior MI, prior myocardial infarction

**Table S4M. The diagnostic performance of cardiologist 12 with 2-year experience, related to Figure 3.**

|  | **ASD**  **(n = 41)** | **DCM**  **(n = 142)** | **HCM**  **(n = 43)** | **prior MI**  **(n = 204)** | **Normal**  **(n = 108)** | **Mean** |
| --- | --- | --- | --- | --- | --- | --- |
| **Accuracy** | 94.05% | 76.21% | 82.34% | 60.97% | 78.81% | 78.48% |
| **Sensitivity** | 24.39% | 25.35% | 88.37% | 52.45% | 53.70% | 48.85% |
| **Specificity** | 99.80% | 94.44% | 81.82% | 66.17% | 85.12% | 85.47% |
| **Error Rate** | 5.95% | 23.79% | 17.66% | 39.03% | 21.19% | 21.52% |

Abbreviation: ASD, atrial septal defect; DCM, dilated cardiomyopathy; HCM, hypertrophic cardiomyopathy; prior MI, prior myocardial infarction

**Table S4N. The diagnostic performance of cardiologist 13 with 1-year experience, related to Figure 3.**

|  | **ASD**  **(n = 41)** | **DCM**  **(n = 142)** | **HCM**  **(n = 43)** | **prior MI**  **(n = 204)** | **Normal**  **(n = 108)** | **Mean** |
| --- | --- | --- | --- | --- | --- | --- |
| **Accuracy** | 89.78% | 77.14% | 93.68% | 65.06% | 80.48% | 81.23% |
| **Sensitivity** | 48.78% | 26.06% | 95.35% | 67.65% | 48.15% | 57.20% |
| **Specificity** | 93.16% | 95.45% | 93.54% | 63.47% | 88.60% | 86.85% |
| **Error Rate** | 10.22% | 22.86% | 6.32% | 34.94% | 19.52% | 18.77% |

Abbreviation: ASD, atrial septal defect; DCM, dilated cardiomyopathy; HCM, hypertrophic cardiomyopathy; prior MI, prior myocardial infarction

**Table S4O. The diagnostic performance of cardiologist 14 with 1-year experience, related to Figure 3.**

|  | **ASD**  **(n = 41)** | **DCM**  **(n = 142)** | **HCM**  **(n = 43)** | **prior MI**  **(n = 204)** | **Normal**  **(n = 108)** | **Mean** |
| --- | --- | --- | --- | --- | --- | --- |
| **Accuracy** | 95.17% | 80.48% | 88.85% | 66.36% | 86.80% | 83.53% |
| **Sensitivity** | 60.98% | 30.28% | 53.49% | 85.78% | 50.00% | 56.11% |
| **Specificity** | 97.99% | 98.48% | 91.92% | 54.49% | 96.05% | 87.79% |
| **Error Rate** | 4.83% | 19.52% | 11.15% | 33.64% | 13.20% | 16.47% |

Abbreviation: ASD, atrial septal defect; DCM, dilated cardiomyopathy; HCM, hypertrophic cardiomyopathy; prior MI, prior myocardial infarction

**Table S4P. The diagnostic performance of cardiologist 15 with 1-year experience, related to Figure 3.**

|  | **ASD**  **(n = 41)** | **DCM**  **(n = 142)** | **HCM**  **(n = 43)** | **prior MI**  **(n = 204)** | **Normal**  **(n = 108)** | **Mean** |
| --- | --- | --- | --- | --- | --- | --- |
| **Accuracy** | 94.42% | 69.89% | 90.33% | 64.50% | 89.03% | 81.64% |
| **Sensitivity** | 87.80% | 51.41% | 81.40% | 49.02% | 53.70% | 64.67% |
| **Specificity** | 94.97% | 76.52% | 91.11% | 73.95% | 97.91% | 86.89% |
| **Error Rate** | 5.58% | 30.11% | 9.67% | 35.50% | 10.97% | 18.36% |

Abbreviation: ASD, atrial septal defect; DCM, dilated cardiomyopathy; HCM, hypertrophic cardiomyopathy; prior MI, prior myocardial infarction

**Table S4Q. The diagnostic performance of cardiologist 16 with 1-year experience, related to Figure 3.**

|  | **ASD**  **(n = 41)** | **DCM**  **(n = 142)** | **HCM**  **(n = 43)** | **prior MI**  **(n = 204)** | **Normal**  **(n = 108)** | **Mean** |
| --- | --- | --- | --- | --- | --- | --- |
| **Accuracy** | 89.78% | 78.07% | 69.70% | 57.81% | 77.70% | 74.61% |
| **Sensitivity** | 31.71% | 40.85% | 88.37% | 25.98% | 32.41% | 43.86% |
| **Specificity** | 94.57% | 91.41% | 68.08% | 77.25% | 89.07% | 84.08% |
| **Error Rate** | 10.22% | 21.93% | 30.30% | 42.19% | 22.30% | 25.39% |

Abbreviation: ASD, atrial septal defect; DCM, dilated cardiomyopathy; HCM, hypertrophic cardiomyopathy; prior MI, prior myocardial infarction

**Table S4R. The diagnostic performance of cardiologist 17 with 0.5-year experience, related to Figure 3.**

|  | **ASD**  **(n = 41)** | **DCM**  **(n = 142)** | **HCM**  **(n = 43)** | **prior MI**  **(n = 204)** | **Normal**  **(n = 108)** | **Mean** |
| --- | --- | --- | --- | --- | --- | --- |
| **Accuracy** | 93.68% | 74.91% | 81.23% | 60.97% | 77.32% | 77.62% |
| **Sensitivity** | 17.07% | 19.72% | 86.05% | 54.41% | 50.00% | 45.45% |
| **Specificity** | 100.00% | 94.70% | 80.81% | 64.97% | 84.19% | 84.93% |
| **Error Rate** | 6.32% | 25.09% | 18.77% | 39.03% | 22.68% | 22.38% |

Abbreviation: ASD, atrial septal defect; DCM, dilated cardiomyopathy; HCM, hypertrophic cardiomyopathy; prior MI, prior myocardial infarction

**Table S5a. Inter-cardiologists and AIEchoDx model variability when diagnosing all five categories estimated with the Cohen’s Kappa statistic**

|  | Gold-standard | AIEchoDx | Consensus | cardiologist 1 | cardiologist 2 | cardiologist 3 | cardiologist 4 | cardiologist 5 | cardiologist 6 | cardiologist 7 | cardiologist 8 | cardiologist 9 | cardiologist 10 | cardiologist 11 | cardiologist 12 | cardiologist 13 | cardiologist 14 | cardiologist 15 | cardiologist 16 | cardiologist 17 |
| --- | --- | --- | --- | --- | --- | --- | --- | --- | --- | --- | --- | --- | --- | --- | --- | --- | --- | --- | --- | --- |
| Gold-standard | 1.00 CIs = [1.00,1.00] | 0.89 CIs = [0.87,0.91] | 0.76 CIs = [0.73,0.80] | 0.68 CIs = [0.64,0.72] | 0.57 CIs = [0.52,0.61] | 0.69 CIs = [0.65,0.73] | 0.74 CIs = [0.70,0.77] | 0.57 CIs = [0.53,0.61] | 0.56 CIs = [0.51,0.60] | 0.55 CIs = [0.51,0.59] | 0.52 CIs = [0.48,0.56] | 0.54 CIs = [0.49,0.58] | 0.59 CIs = [0.55,0.62] | 0.30 CIs = [0.26,0.35] | 0.33 CIs = [0.28,0.37] | 0.41 CIs = [0.37,0.46] | 0.49 CIs = [0.45,0.53] | 0.43 CIs = [0.39,0.48] | 0.21 CIs = [0.17,0.25] | 0.30 CIs = [0.25,0.34] |
| AIEchoDx | 0.89 CIs = [0.87,0.91] | 1.00 CIs = [1.00,1.00] | 0.69 CIs = [0.66,0.73] | 0.62 CIs = [0.58,0.66] | 0.53 CIs = [0.49,0.57] | 0.64 CIs = [0.61,0.68] | 0.66 CIs = [0.62,0.70] | 0.51 CIs = [0.47,0.55] | 0.51 CIs = [0.47,0.56] | 0.52 CIs = [0.48,0.56] | 0.49 CIs = [0.45,0.53] | 0.49 CIs = [0.45,0.53] | 0.53 CIs = [0.49,0.57] | 0.30 CIs = [0.26,0.35] | 0.30 CIs = [0.26,0.35] | 0.38 CIs = [0.33,0.42] | 0.45 CIs = [0.41,0.49] | 0.38 CIs = [0.34,0.42] | 0.19 CIs = [0.15,0.23] | 0.29 CIs = [0.25,0.33] |
| Consensus | 0.76 CIs = [0.73,0.80] | 0.69 CIs = [0.66,0.73] | 1.00 CIs = [1.00,1.00] | 0.83 CIs = [0.80,0.86] | 0.68 CIs = [0.64,0.72] | 0.83 CIs = [0.80,0.86] | 0.75 CIs = [0.71,0.78] | 0.65 CIs = [0.61,0.69] | 0.68 CIs = [0.64,0.72] | 0.67 CIs = [0.63,0.71] | 0.66 CIs = [0.62,0.70] | 0.50 CIs = [0.45,0.54] | 0.52 CIs = [0.48,0.56] | 0.39 CIs = [0.35,0.44] | 0.38 CIs = [0.33,0.42] | 0.47 CIs = [0.42,0.51] | 0.44 CIs = [0.39,0.48] | 0.36 CIs = [0.31,0.40] | 0.23 CIs = [0.19,0.27] | 0.35 CIs = [0.31,0.40] |
| cardiologist 1 | 0.68 CIs = [0.64,0.72] | 0.62 CIs = [0.58,0.66] | 0.83 CIs = [0.80,0.86] | 1.00 CIs = [1.00,1.00] | 0.64 CIs = [0.60,0.68] | 0.67 CIs = [0.63,0.71] | 0.59 CIs = [0.55,0.63] | 0.60 CIs = [0.56,0.65] | 0.68 CIs = [0.64,0.72] | 0.62 CIs = [0.58,0.66] | 0.67 CIs = [0.63,0.71] | 0.43 CIs = [0.39,0.47] | 0.44 CIs = [0.39,0.49] | 0.38 CIs = [0.33,0.42] | 0.35 CIs = [0.30,0.39] | 0.42 CIs = [0.38,0.47] | 0.38 CIs = [0.34,0.42] | 0.30 CIs = [0.25,0.34] | 0.21 CIs = [0.17,0.26] | 0.34 CIs = [0.30,0.38] |
| cardiologist 2 | 0.57 CIs = [0.52,0.61] | 0.53 CIs = [0.48,0.57] | 0.68 CIs = [0.65,0.72] | 0.64 CIs = [0.59,0.68] | 1.00 CIs = [1.00,1.00] | 0.67 CIs = [0.63,0.71] | 0.59 CIs = [0.55,0.62] | 0.61 CIs = [0.57,0.65] | 0.72 CIs = [0.68,0.76] | 0.98 CIs = [0.97,0.99] | 0.71 CIs = [0.67,0.75] | 0.42 CIs = [0.38,0.47] | 0.44 CIs = [0.39,0.48] | 0.37 CIs = [0.32,0.41] | 0.40 CIs = [0.35,0.44] | 0.53 CIs = [0.49,0.57] | 0.43 CIs = [0.39,0.48] | 0.33 CIs = [0.29,0.37] | 0.30 CIs = [0.26,0.35] | 0.38 CIs = [0.34,0.42] |
| cardiologist 3 | 0.69 CIs = [0.65,0.73] | 0.64 CIs = [0.61,0.68] | 0.83 CIs = [0.80,0.86] | 0.67 CIs = [0.63,0.71] | 0.67 CIs = [0.63,0.71] | 1.00 CIs = [1.00,1.00] | 0.59 CIs = [0.55,0.63] | 0.68 CIs = [0.64,0.72] | 0.64 CIs = [0.60,0.68] | 0.65 CIs = [0.61,0.69] | 0.62 CIs = [0.57,0.66] | 0.52 CIs = [0.47,0.56] | 0.52 CIs = [0.47,0.56] | 0.41 CIs = [0.37,0.46] | 0.39 CIs = [0.34,0.43] | 0.48 CIs = [0.44,0.52] | 0.37 CIs = [0.33,0.42] | 0.37 CIs = [0.32,0.41] | 0.27 CIs = [0.23,0.31] | 0.38 CIs = [0.34,0.42] |
| cardiologist 4 | 0.73 CIs = [0.70,0.77] | 0.66 CIs = [0.62,0.70] | 0.75 CIs = [0.72,0.78] | 0.59 CIs = [0.55,0.63] | 0.58 CIs = [0.55,0.63] | 0.59 CIs = [0.55,0.63] | 1.00 CIs = [1.00,1.00] | 0.49 CIs = [0.45,0.53] | 0.52 CIs = [0.48,0.56] | 0.59 CIs = [0.55,0.63] | 0.51 CIs = [0.47,0.56] | 0.48 CIs = [0.43,0.52] | 0.51 CIs = [0.46,0.55] | 0.28 CIs = [0.24,0.33] | 0.36 CIs = [0.32,0.41] | 0.44 CIs = [0.40,0.49] | 0.52 CIs = [0.48,0.56] | 0.40 CIs = [0.36,0.44] | 0.21 CIs = [0.17,0.25] | 0.33 CIs = [0.29,0.37] |
| cardiologist 5 | 0.57 CIs = [0.53,0.61] | 0.51 CIs = [0.47,0.55] | 0.65 CIs = [0.61,0.69] | 0.60 CIs = [0.56,0.64] | 0.61 CIs = [0.57,0.65] | 0.68 CIs = [0.64,0.72] | 0.49 CIs = [0.45,0.53] | 1.00 CIs = [1.00,1.00] | 0.65 CIs = [0.61,0.69] | 0.59 CIs = [0.55,0.63] | 0.61 CIs = [0.57,0.65] | 0.48 CIs = [0.43,0.52] | 0.49 CIs = [0.45,0.54] | 0.36 CIs = [0.32,0.41] | 0.42 CIs = [0.38,0.47] | 0.47 CIs = [0.43,0.51] | 0.37 CIs = [0.33,0.42] | 0.38 CIs = [0.34,0.43] | 0.35 CIs = [0.31,0.39] | 0.40 CIs = [0.36,0.45] |
| cardiologist 6 | 0.55 CIs = [0.51,0.60] | 0.51 CIs = [0.47,0.56] | 0.68 CIs = [0.64,0.72] | 0.68 CIs = [0.64,0.72] | 0.72 CIs = [0.68,0.76] | 0.64 CIs = [0.61,0.68] | 0.52 CIs = [0.48,0.56] | 0.65 CIs = [0.61,0.69] | 1.00 CIs = [1.00,1.00] | 0.72 CIs = [0.68,0.75] | 0.84 CIs = [0.81,0.87] | 0.44 CIs = [0.40,0.48] | 0.43 CIs = [0.38,0.47] | 0.40 CIs = [0.35,0.44] | 0.42 CIs = [0.38,0.47] | 0.50 CIs = [0.45,0.54] | 0.46 CIs = [0.41,0.50] | 0.32 CIs = [0.28,0.37] | 0.25 CIs = [0.21,0.29] | 0.40 CIs = [0.36,0.45] |
| cardiologist 7 | 0.55 CIs = [0.50,0.59] | 0.52 CIs = [0.48,0.56] | 0.67 CIs = [0.63,0.71] | 0.62 CIs = [0.58,0.67] | 0.98 CIs = [0.97,0.99] | 0.65 CIs = [0.61,0.69] | 0.59 CIs = [0.55,0.63] | 0.59 CIs = [0.55,0.64] | 0.72 CIs = [0.67,0.75] | 1.00 CIs = [1.00,1.00] | 0.70 CIs = [0.67,0.74] | 0.41 CIs = [0.36,0.45] | 0.42 CIs = [0.38,0.47] | 0.36 CIs = [0.32,0.41] | 0.40 CIs = [0.36,0.45] | 0.53 CIs = [0.48,0.57] | 0.43 CIs = [0.39,0.48] | 0.32 CIs = [0.28,0.36] | 0.29 CIs = [0.25,0.33] | 0.37 CIs = [0.33,0.42] |
| cardiologist 8 | 0.52 CIs = [0.48,0.57] | 0.49 CIs = [0.45,0.53] | 0.66 CIs = [0.62,0.70] | 0.67 CIs = [0.63,0.71] | 0.71 CIs = [0.67,0.75] | 0.62 CIs = [0.57,0.66] | 0.51 CIs = [0.47,0.56] | 0.61 CIs = [0.56,0.64] | 0.84 CIs = [0.81,0.87] | 0.71 CIs = [0.67,0.74] | 1.00 CIs = [1.00,1.00] | 0.42 CIs = [0.38,0.47] | 0.41 CIs = [0.37,0.45] | 0.38 CIs = [0.33,0.42] | 0.43 CIs = [0.38,0.47] | 0.52 CIs = [0.47,0.56] | 0.46 CIs = [0.42,0.51] | 0.32 CIs = [0.28,0.36] | 0.26 CIs = [0.22,0.30] | 0.39 CIs = [0.35,0.44] |
| cardiologist 9 | 0.54 CIs = [0.50,0.58] | 0.49 CIs = [0.45,0.53] | 0.50 CIs = [0.46,0.54] | 0.43 CIs = [0.39,0.47] | 0.42 CIs = [0.38,0.47] | 0.52 CIs = [0.47,0.56] | 0.48 CIs = [0.43,0.52] | 0.48 CIs = [0.44,0.52] | 0.44 CIs = [0.39,0.48] | 0.41 CIs = [0.36,0.45] | 0.42 CIs = [0.38,0.47] | 1.00 CIs = [1.00,1.00] | 0.77 CIs = [0.73,0.80] | 0.28 CIs = [0.24,0.33] | 0.34 CIs = [0.30,0.39] | 0.35 CIs = [0.30,0.39] | 0.34 CIs = [0.30,0.39] | 0.37 CIs = [0.33,0.42] | 0.26 CIs = [0.22,0.30] | 0.32 CIs = [0.27,0.36] |
| cardiologist 10 | 0.59 CIs = [0.55,0.63] | 0.53 CIs = [0.49,0.57] | 0.52 CIs = [0.48,0.56] | 0.44 CIs = [0.40,0.48] | 0.44 CIs = [0.40,0.48] | 0.52 CIs = [0.48,0.56] | 0.51 CIs = [0.46,0.55] | 0.50 CIs = [0.45,0.54] | 0.43 CIs = [0.39,0.47] | 0.42 CIs = [0.38,0.47] | 0.41 CIs = [0.36,0.45] | 0.77 CIs = [0.73,0.80] | 1.00 CIs = [1.00,1.00] | 0.27 CIs = [0.23,0.32] | 0.33 CIs = [0.29,0.37] | 0.35 CIs = [0.31,0.40] | 0.38 CIs = [0.34,0.43] | 0.40 CIs = [0.35,0.44] | 0.27 CIs = [0.22,0.31] | 0.30 CIs = [0.26,0.35] |
| cardiologist 11 | 0.30 CIs = [0.26,0.35] | 0.30 CIs = [0.26,0.35] | 0.40 CIs = [0.35,0.44] | 0.38 CIs = [0.34,0.42] | 0.37 CIs = [0.32,0.41] | 0.42 CIs = [0.37,0.46] | 0.28 CIs = [0.24,0.33] | 0.36 CIs = [0.32,0.41] | 0.39 CIs = [0.35,0.44] | 0.37 CIs = [0.32,0.41] | 0.38 CIs = [0.33,0.42] | 0.28 CIs = [0.24,0.33] | 0.27 CIs = [0.23,0.32] | 1.00 CIs = [1.00,1.00] | 0.33 CIs = [0.29,0.38] | 0.27 CIs = [0.23,0.31] | 0.13 CIs = [0.09,0.18] | 0.22 CIs = [0.18,0.26] | 0.21 CIs = [0.17,0.25] | 0.31 CIs = [0.26,0.35] |
| cardiologist 12 | 0.33 CIs = [0.29,0.38] | 0.30 CIs = [0.26,0.35] | 0.38 CIs = [0.33,0.42] | 0.35 CIs = [0.31,0.39] | 0.40 CIs = [0.35,0.44] | 0.39 CIs = [0.34,0.43] | 0.36 CIs = [0.32,0.41] | 0.42 CIs = [0.38,0.47] | 0.42 CIs = [0.38,0.46] | 0.40 CIs = [0.35,0.44] | 0.43 CIs = [0.38,0.47] | 0.34 CIs = [0.30,0.38] | 0.33 CIs = [0.29,0.38] | 0.33 CIs = [0.29,0.38] | 1.00 CIs = [1.00,1.00] | 0.42 CIs = [0.37,0.46] | 0.35 CIs = [0.30,0.40] | 0.28 CIs = [0.24,0.32] | 0.33 CIs = [0.28,0.37] | 0.88 CIs = [0.85,0.90] |
| cardiologist 13 | 0.42 CIs = [0.37,0.46] | 0.38 CIs = [0.34,0.42] | 0.47 CIs = [0.42,0.51] | 0.42 CIs = [0.38,0.46] | 0.53 CIs = [0.48,0.57] | 0.48 CIs = [0.44,0.52] | 0.44 CIs = [0.40,0.49] | 0.47 CIs = [0.43,0.51] | 0.50 CIs = [0.46,0.54] | 0.52 CIs = [0.48,0.57] | 0.51 CIs = [0.47,0.56] | 0.35 CIs = [0.30,0.39] | 0.35 CIs = [0.31,0.40] | 0.27 CIs = [0.22,0.31] | 0.41 CIs = [0.37,0.46] | 1.00 CIs = [1.00,1.00] | 0.45 CIs = [0.41,0.50] | 0.33 CIs = [0.28,0.37] | 0.30 CIs = [0.25,0.34] | 0.44 CIs = [0.39,0.48] |
| cardiologist 14 | 0.49 CIs = [0.45,0.53] | 0.45 CIs = [0.41,0.49] | 0.44 CIs = [0.40,0.48] | 0.38 CIs = [0.34,0.42] | 0.43 CIs = [0.39,0.48] | 0.37 CIs = [0.33,0.42] | 0.52 CIs = [0.48,0.56] | 0.38 CIs = [0.33,0.42] | 0.46 CIs = [0.41,0.50] | 0.43 CIs = [0.39,0.48] | 0.46 CIs = [0.42,0.51] | 0.34 CIs = [0.30,0.39] | 0.38 CIs = [0.34,0.43] | 0.13 CIs = [0.09,0.18] | 0.35 CIs = [0.30,0.39] | 0.45 CIs = [0.41,0.50] | 1.00 CIs = [1.00,1.00] | 0.31 CIs = [0.27,0.35] | 0.19 CIs = [0.15,0.23] | 0.33 CIs = [0.28,0.37] |
| cardiologist 15 | 0.43 CIs = [0.39,0.48] | 0.38 CIs = [0.34,0.42] | 0.36 CIs = [0.31,0.40] | 0.30 CIs = [0.25,0.34] | 0.33 CIs = [0.29,0.38] | 0.37 CIs = [0.32,0.41] | 0.40 CIs = [0.35,0.44] | 0.38 CIs = [0.34,0.43] | 0.32 CIs = [0.28,0.37] | 0.32 CIs = [0.28,0.37] | 0.32 CIs = [0.28,0.36] | 0.38 CIs = [0.33,0.42] | 0.39 CIs = [0.35,0.44] | 0.22 CIs = [0.18,0.26] | 0.28 CIs = [0.24,0.32] | 0.32 CIs = [0.29,0.36] | 0.31 CIs = [0.27,0.35] | 1.00 CIs = [1.00,1.00] | 0.26 CIs = [0.21,0.30] | 0.27 CIs = [0.23,0.31] |
| cardiologist 16 | 0.21 CIs = [0.16,0.25] | 0.19 CIs = [0.15,0.23] | 0.23 CIs = [0.19,0.27] | 0.21 CIs = [0.17,0.26] | 0.30 CIs = [0.25,0.34] | 0.27 CIs = [0.22,0.31] | 0.21 CIs = [0.17,0.26] | 0.35 CIs = [0.31,0.39] | 0.25 CIs = [0.21,0.29] | 0.29 CIs = [0.24,0.33] | 0.26 CIs = [0.22,0.30] | 0.26 CIs = [0.22,0.31] | 0.27 CIs = [0.23,0.31] | 0.21 CIs = [0.17,0.25] | 0.33 CIs = [0.28,0.37] | 0.30 CIs = [0.26,0.34] | 0.19 CIs = [0.14,0.23] | 0.26 CIs = [0.21,0.30] | 1.00 CIs = [1.00,1.00] | 0.33 CIs = [0.28,0.38] |
| cardiologist 17 | 0.30 CIs = [0.26,0.34] | 0.29 CIs = [0.25,0.33] | 0.35 CIs = [0.31,0.40] | 0.34 CIs = [0.30,0.38] | 0.38 CIs = [0.33,0.42] | 0.38 CIs = [0.34,0.43] | 0.33 CIs = [0.29,0.38] | 0.41 CIs = [0.36,0.45] | 0.40 CIs = [0.36,0.45] | 0.37 CIs = [0.33,0.42] | 0.39 CIs = [0.35,0.44] | 0.32 CIs = [0.27,0.36] | 0.30 CIs = [0.26,0.34] | 0.31 CIs = [0.26,0.35] | 0.88 CIs = [0.86,0.91] | 0.44 CIs = [0.40,0.48] | 0.33 CIs = [0.29,0.37] | 0.27 CIs = [0.23,0.32] | 0.33 CIs = [0.28,0.37] | 1.00 CIs = [1.00,1.00] |

**Table S5b. Inter-cardiologists and AIEchoDx model variability when diagnosing ASD and non-ASD patients estimated with the Cohen’s Kappa statistic**

|  | Ground-true | AIEchoDx | Consensus | cardiologist 1 | cardiologist 2 | cardiologist 3 | cardiologist 4 | cardiologist 5 | cardiologist 6 | cardiologist 7 | cardiologist 8 | cardiologist 9 | cardiologist 10 | cardiologist 11 | cardiologist 12 | cardiologist 13 | cardiologist 14 | cardiologist 15 | cardiologist 16 | cardiologist 17 |
| --- | --- | --- | --- | --- | --- | --- | --- | --- | --- | --- | --- | --- | --- | --- | --- | --- | --- | --- | --- | --- |
| Ground-true | 1.00 CIs = [1.00,1.00] | 0.88 CIs = [0.81,0.94] | 0.95 CIs = [0.90,0.99] | 0.80 CIs = [0.70,0.88] | 0.75 CIs = [0.66,0.83] | 0.89 CIs = [0.83,0.96] | 0.88 CIs = [0.80,0.93] | 0.77 CIs = [0.67,0.86] | 0.67 CIs = [0.55,0.78] | 0.73 CIs = [0.64,0.82] | 0.67 CIs = [0.56,0.79] | 0.66 CIs = [0.55,0.77] | 0.71 CIs = [0.60,0.80] | 0.45 CIs = [0.31,0.58] | 0.36 CIs = [0.22,0.50] | 0.37 CIs = [0.25,0.47] | 0.63 CIs = [0.51,0.74] | 0.67 CIs = [0.58,0.76] | 0.26 CIs = [0.15,0.38] | 0.27 CIs = [0.14,0.43] |
| AIEchoDx | 0.88 CIs = [0.81,0.94] | 1.00 CIs = [1.00,1.00] | 0.82 CIs = [0.73,0.90] | 0.70 CIs = [0.60,0.81] | 0.64 CIs = [0.52,0.75] | 0.77 CIs = [0.66,0.85] | 0.75 CIs = [0.67,0.84] | 0.68 CIs = [0.57,0.79] | 0.53 CIs = [0.39,0.66] | 0.65 CIs = [0.53,0.76] | 0.56 CIs = [0.43,0.69] | 0.59 CIs = [0.46,0.71] | 0.64 CIs = [0.52,0.75] | 0.45 CIs = [0.31,0.58] | 0.29 CIs = [0.14,0.44] | 0.36 CIs = [0.25,0.47] | 0.59 CIs = [0.46,0.70] | 0.59 CIs = [0.48,0.68] | 0.28 CIs = [0.15,0.41] | 0.28 CIs = [0.12,0.44] |
| Consensus | 0.95 CIs = [0.90,0.99] | 0.82 CIs = [0.73,0.90] | 1.00 CIs = [1.00,1.00] | 0.82 CIs = [0.73,0.90] | 0.75 CIs = [0.64,0.83] | 0.95 CIs = [0.89,0.99] | 0.87 CIs = [0.80,0.93] | 0.77 CIs = [0.68,0.85] | 0.69 CIs = [0.58,0.79] | 0.73 CIs = [0.63,0.82] | 0.70 CIs = [0.58,0.80] | 0.68 CIs = [0.57,0.78] | 0.67 CIs = [0.55,0.77] | 0.47 CIs = [0.34,0.59] | 0.34 CIs = [0.19,0.48] | 0.35 CIs = [0.23,0.46] | 0.62 CIs = [0.50,0.73] | 0.70 CIs = [0.59,0.78] | 0.28 CIs = [0.16,0.39] | 0.24 CIs = [0.10,0.38] |
| cardiologist 1 | 0.79 CIs = [0.70,0.88] | 0.71 CIs = [0.59,0.81] | 0.82 CIs = [0.73,0.90] | 1.00 CIs = [1.00,1.00] | 0.75 CIs = [0.66,0.85] | 0.77 CIs = [0.66,0.86] | 0.70 CIs = [0.60,0.80] | 0.81 CIs = [0.71,0.89] | 0.80 CIs = [0.71,0.89] | 0.74 CIs = [0.64,0.83] | 0.81 CIs = [0.71,0.89] | 0.76 CIs = [0.65,0.85] | 0.64 CIs = [0.52,0.76] | 0.52 CIs = [0.38,0.65] | 0.34 CIs = [0.17,0.49] | 0.29 CIs = [0.18,0.40] | 0.59 CIs = [0.47,0.71] | 0.63 CIs = [0.53,0.73] | 0.22 CIs = [0.11,0.34] | 0.23 CIs = [0.09,0.37] |
| cardiologist 2 | 0.75 CIs = [0.66,0.83] | 0.64 CIs = [0.53,0.75] | 0.74 CIs = [0.65,0.83] | 0.76 CIs = [0.65,0.85] | 1.00 CIs = [1.00,1.00] | 0.72 CIs = [0.62,0.81] | 0.81 CIs = [0.74,0.89] | 0.73 CIs = [0.64,0.82] | 0.69 CIs = [0.58,0.79] | 0.99 CIs = [0.96,1.00] | 0.69 CIs = [0.58,0.79] | 0.62 CIs = [0.51,0.74] | 0.61 CIs = [0.48,0.72] | 0.54 CIs = [0.40,0.66] | 0.35 CIs = [0.22,0.49] | 0.36 CIs = [0.25,0.46] | 0.59 CIs = [0.47,0.70] | 0.69 CIs = [0.60,0.77] | 0.29 CIs = [0.16,0.41] | 0.22 CIs = [0.10,0.36] |
| cardiologist 3 | 0.90 CIs = [0.83,0.95] | 0.76 CIs = [0.67,0.86] | 0.95 CIs = [0.89,0.99] | 0.76 CIs = [0.67,0.85] | 0.72 CIs = [0.63,0.81] | 1.00 CIs = [1.00,1.00] | 0.82 CIs = [0.75,0.90] | 0.74 CIs = [0.65,0.84] | 0.67 CIs = [0.55,0.77] | 0.71 CIs = [0.60,0.80] | 0.67 CIs = [0.56,0.77] | 0.66 CIs = [0.54,0.76] | 0.68 CIs = [0.57,0.78] | 0.45 CIs = [0.32,0.57] | 0.32 CIs = [0.19,0.45] | 0.34 CIs = [0.23,0.45] | 0.66 CIs = [0.54,0.76] | 0.68 CIs = [0.58,0.76] | 0.26 CIs = [0.15,0.37] | 0.24 CIs = [0.11,0.37] |
| cardiologist 4 | 0.87 CIs = [0.81,0.94] | 0.76 CIs = [0.66,0.84] | 0.87 CIs = [0.80,0.93] | 0.70 CIs = [0.60,0.80] | 0.82 CIs = [0.73,0.89] | 0.83 CIs = [0.75,0.90] | 1.00 CIs = [1.00,1.00] | 0.68 CIs = [0.58,0.78] | 0.58 CIs = [0.47,0.69] | 0.80 CIs = [0.71,0.88] | 0.58 CIs = [0.47,0.69] | 0.63 CIs = [0.52,0.74] | 0.59 CIs = [0.48,0.71] | 0.44 CIs = [0.31,0.55] | 0.35 CIs = [0.23,0.48] | 0.35 CIs = [0.25,0.47] | 0.63 CIs = [0.52,0.73] | 0.69 CIs = [0.60,0.77] | 0.28 CIs = [0.18,0.40] | 0.24 CIs = [0.11,0.37] |
| cardiologist 5 | 0.77 CIs = [0.67,0.85] | 0.68 CIs = [0.57,0.78] | 0.77 CIs = [0.67,0.86] | 0.81 CIs = [0.72,0.89] | 0.73 CIs = [0.63,0.82] | 0.75 CIs = [0.65,0.83] | 0.68 CIs = [0.58,0.79] | 1.00 CIs = [1.00,1.00] | 0.74 CIs = [0.64,0.83] | 0.72 CIs = [0.61,0.81] | 0.74 CIs = [0.63,0.84] | 0.66 CIs = [0.55,0.77] | 0.65 CIs = [0.54,0.76] | 0.50 CIs = [0.36,0.63] | 0.32 CIs = [0.18,0.48] | 0.25 CIs = [0.14,0.36] | 0.63 CIs = [0.52,0.74] | 0.61 CIs = [0.51,0.70] | 0.29 CIs = [0.16,0.43] | 0.26 CIs = [0.11,0.42] |
| cardiologist 6 | 0.67 CIs = [0.56,0.77] | 0.53 CIs = [0.40,0.65] | 0.69 CIs = [0.58,0.79] | 0.80 CIs = [0.70,0.89] | 0.69 CIs = [0.58,0.79] | 0.67 CIs = [0.55,0.77] | 0.59 CIs = [0.47,0.69] | 0.74 CIs = [0.62,0.83] | 1.00 CIs = [1.00,1.00] | 0.67 CIs = [0.56,0.77] | 0.94 CIs = [0.89,1.00] | 0.68 CIs = [0.55,0.80] | 0.56 CIs = [0.44,0.69] | 0.66 CIs = [0.53,0.77] | 0.41 CIs = [0.24,0.57] | 0.20 CIs = [0.09,0.31] | 0.55 CIs = [0.43,0.66] | 0.55 CIs = [0.45,0.65] | 0.20 CIs = [0.07,0.32] | 0.30 CIs = [0.13,0.45] |
| cardiologist 7 | 0.73 CIs = [0.64,0.82] | 0.65 CIs = [0.53,0.76] | 0.73 CIs = [0.62,0.82] | 0.74 CIs = [0.64,0.83] | 0.99 CIs = [0.96,1.00] | 0.70 CIs = [0.60,0.80] | 0.80 CIs = [0.71,0.88] | 0.72 CIs = [0.61,0.81] | 0.67 CIs = [0.56,0.78] | 1.00 CIs = [1.00,1.00] | 0.70 CIs = [0.60,0.80] | 0.60 CIs = [0.47,0.72] | 0.59 CIs = [0.47,0.71] | 0.54 CIs = [0.42,0.67] | 0.36 CIs = [0.22,0.50] | 0.36 CIs = [0.24,0.47] | 0.60 CIs = [0.48,0.71] | 0.67 CIs = [0.58,0.76] | 0.29 CIs = [0.17,0.41] | 0.23 CIs = [0.11,0.37] |
| cardiologist 8 | 0.68 CIs = [0.56,0.78] | 0.56 CIs = [0.42,0.69] | 0.70 CIs = [0.58,0.80] | 0.81 CIs = [0.70,0.89] | 0.69 CIs = [0.59,0.79] | 0.68 CIs = [0.56,0.78] | 0.58 CIs = [0.46,0.69] | 0.74 CIs = [0.62,0.84] | 0.95 CIs = [0.88,0.99] | 0.71 CIs = [0.59,0.80] | 1.00 CIs = [1.00,1.00] | 0.64 CIs = [0.51,0.76] | 0.53 CIs = [0.39,0.66] | 0.66 CIs = [0.53,0.77] | 0.44 CIs = [0.28,0.61] | 0.22 CIs = [0.11,0.32] | 0.57 CIs = [0.44,0.70] | 0.53 CIs = [0.42,0.63] | 0.19 CIs = [0.07,0.30] | 0.33 CIs = [0.16,0.50] |
| cardiologist 9 | 0.67 CIs = [0.55,0.77] | 0.59 CIs = [0.46,0.70] | 0.68 CIs = [0.57,0.78] | 0.76 CIs = [0.65,0.85] | 0.62 CIs = [0.50,0.73] | 0.66 CIs = [0.55,0.77] | 0.63 CIs = [0.52,0.73] | 0.67 CIs = [0.53,0.78] | 0.68 CIs = [0.56,0.80] | 0.60 CIs = [0.48,0.72] | 0.65 CIs = [0.52,0.76] | 1.00 CIs = [1.00,1.00] | 0.76 CIs = [0.65,0.86] | 0.49 CIs = [0.36,0.62] | 0.38 CIs = [0.20,0.53] | 0.23 CIs = [0.12,0.34] | 0.54 CIs = [0.39,0.66] | 0.57 CIs = [0.46,0.67] | 0.24 CIs = [0.11,0.37] | 0.25 CIs = [0.10,0.42] |
| cardiologist 10 | 0.71 CIs = [0.59,0.80] | 0.64 CIs = [0.52,0.75] | 0.67 CIs = [0.56,0.77] | 0.65 CIs = [0.52,0.75] | 0.61 CIs = [0.50,0.72] | 0.68 CIs = [0.57,0.78] | 0.59 CIs = [0.48,0.70] | 0.65 CIs = [0.54,0.76] | 0.57 CIs = [0.44,0.68] | 0.59 CIs = [0.48,0.70] | 0.52 CIs = [0.39,0.65] | 0.76 CIs = [0.65,0.85] | 1.00 CIs = [1.00,1.00] | 0.41 CIs = [0.27,0.55] | 0.29 CIs = [0.15,0.44] | 0.29 CIs = [0.17,0.40] | 0.53 CIs = [0.40,0.65] | 0.54 CIs = [0.43,0.64] | 0.19 CIs = [0.08,0.30] | 0.23 CIs = [0.10,0.38] |
| cardiologist 11 | 0.45 CIs = [0.32,0.57] | 0.44 CIs = [0.30,0.59] | 0.47 CIs = [0.33,0.60] | 0.52 CIs = [0.38,0.65] | 0.53 CIs = [0.42,0.66] | 0.45 CIs = [0.31,0.57] | 0.43 CIs = [0.32,0.55] | 0.50 CIs = [0.37,0.64] | 0.66 CIs = [0.52,0.77] | 0.55 CIs = [0.41,0.67] | 0.66 CIs = [0.52,0.78] | 0.49 CIs = [0.34,0.62] | 0.41 CIs = [0.26,0.55] | 1.00 CIs = [1.00,1.00] | 0.30 CIs = [0.14,0.48] | 0.17 CIs = [0.07,0.28] | 0.36 CIs = [0.23,0.50] | 0.43 CIs = [0.31,0.54] | 0.24 CIs = [0.11,0.36] | 0.30 CIs = [0.12,0.46] |
| cardiologist 12 | 0.36 CIs = [0.23,0.49] | 0.29 CIs = [0.14,0.46] | 0.34 CIs = [0.19,0.47] | 0.33 CIs = [0.17,0.49] | 0.36 CIs = [0.22,0.49] | 0.32 CIs = [0.18,0.45] | 0.35 CIs = [0.22,0.48] | 0.33 CIs = [0.17,0.47] | 0.40 CIs = [0.25,0.57] | 0.36 CIs = [0.21,0.49] | 0.44 CIs = [0.27,0.61] | 0.38 CIs = [0.21,0.54] | 0.29 CIs = [0.14,0.45] | 0.31 CIs = [0.14,0.48] | 1.00 CIs = [1.00,1.00] | 0.09 CIs = [0.01,0.19] | 0.32 CIs = [0.16,0.49] | 0.25 CIs = [0.15,0.36] | 0.13 CIs = [0.02,0.26] | 0.64 CIs = [0.40,0.86] |
| cardiologist 13 | 0.36 CIs = [0.26,0.48] | 0.36 CIs = [0.25,0.48] | 0.35 CIs = [0.24,0.46] | 0.29 CIs = [0.17,0.40] | 0.36 CIs = [0.24,0.47] | 0.34 CIs = [0.22,0.45] | 0.35 CIs = [0.25,0.45] | 0.25 CIs = [0.14,0.36] | 0.20 CIs = [0.11,0.31] | 0.36 CIs = [0.25,0.47] | 0.21 CIs = [0.11,0.32] | 0.24 CIs = [0.12,0.34] | 0.29 CIs = [0.18,0.39] | 0.18 CIs = [0.07,0.28] | 0.09 CIs = [0.00,0.19] | 1.00 CIs = [1.00,1.00] | 0.31 CIs = [0.19,0.41] | 0.33 CIs = [0.22,0.43] | 0.19 CIs = [0.08,0.28] | 0.11 CIs = [0.02,0.20] |
| cardiologist 14 | 0.63 CIs = [0.52,0.73] | 0.59 CIs = [0.46,0.71] | 0.62 CIs = [0.50,0.73] | 0.59 CIs = [0.47,0.70] | 0.59 CIs = [0.48,0.69] | 0.66 CIs = [0.55,0.76] | 0.63 CIs = [0.52,0.73] | 0.63 CIs = [0.51,0.74] | 0.55 CIs = [0.41,0.67] | 0.60 CIs = [0.49,0.71] | 0.57 CIs = [0.44,0.70] | 0.53 CIs = [0.40,0.66] | 0.53 CIs = [0.40,0.65] | 0.36 CIs = [0.23,0.49] | 0.33 CIs = [0.18,0.47] | 0.30 CIs = [0.19,0.42] | 1.00 CIs = [1.00,1.00] | 0.49 CIs = [0.39,0.60] | 0.24 CIs = [0.12,0.35] | 0.27 CIs = [0.11,0.41] |
| cardiologist 15 | 0.68 CIs = [0.58,0.77] | 0.58 CIs = [0.48,0.69] | 0.69 CIs = [0.61,0.78] | 0.63 CIs = [0.53,0.73] | 0.69 CIs = [0.60,0.78] | 0.68 CIs = [0.58,0.76] | 0.69 CIs = [0.61,0.78] | 0.61 CIs = [0.51,0.70] | 0.55 CIs = [0.45,0.65] | 0.67 CIs = [0.58,0.75] | 0.53 CIs = [0.42,0.64] | 0.56 CIs = [0.46,0.67] | 0.54 CIs = [0.42,0.64] | 0.42 CIs = [0.31,0.54] | 0.25 CIs = [0.14,0.37] | 0.33 CIs = [0.22,0.43] | 0.50 CIs = [0.39,0.60] | 1.00 CIs = [1.00,1.00] | 0.34 CIs = [0.23,0.44] | 0.16 CIs = [0.06,0.26] |
| cardiologist 16 | 0.27 CIs = [0.14,0.38] | 0.28 CIs = [0.16,0.40] | 0.27 CIs = [0.16,0.39] | 0.22 CIs = [0.10,0.33] | 0.28 CIs = [0.17,0.41] | 0.26 CIs = [0.14,0.38] | 0.29 CIs = [0.16,0.40] | 0.29 CIs = [0.17,0.41] | 0.20 CIs = [0.09,0.32] | 0.29 CIs = [0.17,0.40] | 0.19 CIs = [0.07,0.30] | 0.24 CIs = [0.12,0.36] | 0.19 CIs = [0.07,0.31] | 0.23 CIs = [0.11,0.37] | 0.13 CIs = [0.02,0.25] | 0.18 CIs = [0.09,0.29] | 0.24 CIs = [0.12,0.35] | 0.34 CIs = [0.23,0.44] | 1.00 CIs = [1.00,1.00] | 0.15 CIs = [0.04,0.28] |
| cardiologist 17 | 0.28 CIs = [0.14,0.42] | 0.28 CIs = [0.14,0.44] | 0.24 CIs = [0.10,0.38] | 0.23 CIs = [0.07,0.37] | 0.22 CIs = [0.10,0.36] | 0.23 CIs = [0.10,0.36] | 0.24 CIs = [0.12,0.36] | 0.27 CIs = [0.13,0.41] | 0.30 CIs = [0.13,0.46] | 0.23 CIs = [0.10,0.36] | 0.33 CIs = [0.15,0.50] | 0.26 CIs = [0.09,0.42] | 0.23 CIs = [0.10,0.39] | 0.29 CIs = [0.13,0.47] | 0.65 CIs = [0.39,0.84] | 0.11 CIs = [0.02,0.21] | 0.27 CIs = [0.11,0.43] | 0.16 CIs = [0.06,0.25] | 0.15 CIs = [0.03,0.28] | 1.00 CIs = [1.00,1.00] |

**Table S5c. Inter-cardiologists and AIEchoDx model variability when diagnosing DCM and non-DCM patients estimated with the Cohen’s Kappa statistic**

|  | Ground-true | AIEchoDx | Consensus | cardiologist 1 | cardiologist 2 | cardiologist 3 | cardiologist 4 | cardiologist 5 | cardiologist 6 | cardiologist 7 | cardiologist 8 | cardiologist 9 | cardiologist 10 | cardiologist 11 | cardiologist 12 | cardiologist 13 | cardiologist 14 | cardiologist 15 | cardiologist 16 | cardiologist 17 |
| --- | --- | --- | --- | --- | --- | --- | --- | --- | --- | --- | --- | --- | --- | --- | --- | --- | --- | --- | --- | --- |
| Ground-true | 1.00 CIs = [1.00,1.00] | 0.91 CIs = [0.87,0.94] | 0.80 CIs = [0.74,0.85] | 0.81 CIs = [0.77,0.86] | 0.53 CIs = [0.46,0.60] | 0.78 CIs = [0.73,0.83] | 0.68 CIs = [0.61,0.74] | 0.62 CIs = [0.55,0.68] | 0.53 CIs = [0.45,0.59] | 0.50 CIs = [0.43,0.57] | 0.42 CIs = [0.35,0.50] | 0.54 CIs = [0.47,0.60] | 0.61 CIs = [0.55,0.67] | 0.35 CIs = [0.28,0.43] | 0.24 CIs = [0.17,0.32] | 0.27 CIs = [0.19,0.34] | 0.36 CIs = [0.29,0.43] | 0.26 CIs = [0.20,0.33] | 0.36 CIs = [0.29,0.44] | 0.18 CIs = [0.11,0.25] |
| AIEchoDx | 0.91 CIs = [0.87,0.94] | 1.00 CIs = [1.00,1.00] | 0.77 CIs = [0.72,0.82] | 0.80 CIs = [0.75,0.85] | 0.53 CIs = [0.45,0.60] | 0.77 CIs = [0.71,0.82] | 0.65 CIs = [0.58,0.71] | 0.61 CIs = [0.54,0.68] | 0.54 CIs = [0.46,0.61] | 0.50 CIs = [0.43,0.58] | 0.46 CIs = [0.38,0.53] | 0.49 CIs = [0.42,0.55] | 0.54 CIs = [0.48,0.61] | 0.36 CIs = [0.29,0.45] | 0.22 CIs = [0.13,0.30] | 0.23 CIs = [0.15,0.31] | 0.33 CIs = [0.25,0.41] | 0.24 CIs = [0.16,0.31] | 0.32 CIs = [0.24,0.40] | 0.19 CIs = [0.12,0.26] |
| Consensus | 0.80 CIs = [0.75,0.85] | 0.77 CIs = [0.71,0.83] | 1.00 CIs = [1.00,1.00] | 0.89 CIs = [0.84,0.93] | 0.62 CIs = [0.54,0.69] | 0.86 CIs = [0.81,0.90] | 0.85 CIs = [0.80,0.90] | 0.64 CIs = [0.57,0.71] | 0.61 CIs = [0.53,0.68] | 0.59 CIs = [0.51,0.66] | 0.51 CIs = [0.43,0.59] | 0.47 CIs = [0.40,0.54] | 0.58 CIs = [0.51,0.64] | 0.37 CIs = [0.29,0.45] | 0.28 CIs = [0.19,0.36] | 0.29 CIs = [0.20,0.37] | 0.41 CIs = [0.33,0.50] | 0.22 CIs = [0.15,0.29] | 0.35 CIs = [0.26,0.44] | 0.22 CIs = [0.14,0.31] |
| cardiologist 1 | 0.81 CIs = [0.76,0.86] | 0.80 CIs = [0.74,0.85] | 0.89 CIs = [0.85,0.93] | 1.00 CIs = [1.00,1.00] | 0.55 CIs = [0.47,0.63] | 0.75 CIs = [0.69,0.80] | 0.74 CIs = [0.68,0.79] | 0.60 CIs = [0.52,0.67] | 0.54 CIs = [0.47,0.62] | 0.52 CIs = [0.44,0.60] | 0.47 CIs = [0.38,0.56] | 0.44 CIs = [0.36,0.51] | 0.53 CIs = [0.46,0.59] | 0.31 CIs = [0.22,0.40] | 0.25 CIs = [0.16,0.33] | 0.25 CIs = [0.16,0.33] | 0.37 CIs = [0.29,0.46] | 0.21 CIs = [0.14,0.28] | 0.31 CIs = [0.22,0.39] | 0.20 CIs = [0.12,0.28] |
| cardiologist 2 | 0.53 CIs = [0.45,0.60] | 0.53 CIs = [0.46,0.61] | 0.62 CIs = [0.54,0.68] | 0.55 CIs = [0.47,0.62] | 1.00 CIs = [1.00,1.00] | 0.65 CIs = [0.57,0.71] | 0.54 CIs = [0.46,0.62] | 0.65 CIs = [0.58,0.72] | 0.74 CIs = [0.67,0.81] | 0.96 CIs = [0.92,0.98] | 0.68 CIs = [0.59,0.76] | 0.40 CIs = [0.33,0.47] | 0.46 CIs = [0.39,0.53] | 0.24 CIs = [0.15,0.33] | 0.25 CIs = [0.16,0.35] | 0.45 CIs = [0.35,0.54] | 0.46 CIs = [0.36,0.55] | 0.19 CIs = [0.13,0.26] | 0.45 CIs = [0.37,0.54] | 0.18 CIs = [0.08,0.28] |
| cardiologist 3 | 0.78 CIs = [0.73,0.83] | 0.77 CIs = [0.71,0.82] | 0.86 CIs = [0.81,0.90] | 0.75 CIs = [0.69,0.81] | 0.64 CIs = [0.57,0.71] | 1.00 CIs = [1.00,1.00] | 0.70 CIs = [0.63,0.77] | 0.72 CIs = [0.65,0.78] | 0.63 CIs = [0.55,0.69] | 0.61 CIs = [0.54,0.68] | 0.53 CIs = [0.45,0.61] | 0.51 CIs = [0.44,0.59] | 0.59 CIs = [0.52,0.65] | 0.39 CIs = [0.31,0.48] | 0.29 CIs = [0.20,0.37] | 0.35 CIs = [0.27,0.44] | 0.38 CIs = [0.30,0.46] | 0.26 CIs = [0.19,0.34] | 0.41 CIs = [0.33,0.49] | 0.26 CIs = [0.18,0.35] |
| cardiologist 4 | 0.68 CIs = [0.62,0.74] | 0.64 CIs = [0.58,0.71] | 0.85 CIs = [0.80,0.90] | 0.74 CIs = [0.67,0.80] | 0.54 CIs = [0.46,0.62] | 0.70 CIs = [0.63,0.77] | 1.00 CIs = [1.00,1.00] | 0.52 CIs = [0.44,0.59] | 0.54 CIs = [0.45,0.62] | 0.54 CIs = [0.46,0.62] | 0.44 CIs = [0.36,0.54] | 0.42 CIs = [0.34,0.49] | 0.48 CIs = [0.41,0.55] | 0.39 CIs = [0.30,0.47] | 0.31 CIs = [0.21,0.40] | 0.23 CIs = [0.14,0.32] | 0.40 CIs = [0.31,0.49] | 0.22 CIs = [0.15,0.29] | 0.30 CIs = [0.21,0.38] | 0.22 CIs = [0.13,0.31] |
| cardiologist 5 | 0.62 CIs = [0.56,0.68] | 0.61 CIs = [0.54,0.67] | 0.65 CIs = [0.58,0.72] | 0.60 CIs = [0.53,0.67] | 0.65 CIs = [0.57,0.72] | 0.72 CIs = [0.65,0.78] | 0.52 CIs = [0.44,0.59] | 1.00 CIs = [1.00,1.00] | 0.68 CIs = [0.61,0.76] | 0.61 CIs = [0.54,0.69] | 0.61 CIs = [0.54,0.69] | 0.56 CIs = [0.49,0.63] | 0.57 CIs = [0.51,0.64] | 0.29 CIs = [0.20,0.37] | 0.27 CIs = [0.18,0.35] | 0.43 CIs = [0.34,0.51] | 0.41 CIs = [0.32,0.50] | 0.35 CIs = [0.27,0.42] | 0.50 CIs = [0.43,0.58] | 0.23 CIs = [0.14,0.31] |
| cardiologist 6 | 0.52 CIs = [0.45,0.60] | 0.53 CIs = [0.46,0.60] | 0.61 CIs = [0.53,0.68] | 0.54 CIs = [0.46,0.62] | 0.74 CIs = [0.67,0.81] | 0.62 CIs = [0.55,0.69] | 0.54 CIs = [0.45,0.63] | 0.69 CIs = [0.61,0.75] | 1.00 CIs = [1.00,1.00] | 0.75 CIs = [0.67,0.82] | 0.78 CIs = [0.70,0.85] | 0.45 CIs = [0.38,0.52] | 0.46 CIs = [0.40,0.54] | 0.25 CIs = [0.15,0.33] | 0.31 CIs = [0.22,0.41] | 0.42 CIs = [0.32,0.51] | 0.49 CIs = [0.39,0.58] | 0.21 CIs = [0.15,0.28] | 0.36 CIs = [0.27,0.45] | 0.26 CIs = [0.17,0.35] |
| cardiologist 7 | 0.50 CIs = [0.42,0.57] | 0.51 CIs = [0.43,0.58] | 0.59 CIs = [0.52,0.67] | 0.52 CIs = [0.45,0.60] | 0.96 CIs = [0.92,0.98] | 0.61 CIs = [0.54,0.68] | 0.54 CIs = [0.45,0.62] | 0.61 CIs = [0.53,0.69] | 0.75 CIs = [0.67,0.82] | 1.00 CIs = [1.00,1.00] | 0.66 CIs = [0.57,0.75] | 0.38 CIs = [0.31,0.45] | 0.44 CIs = [0.37,0.51] | 0.25 CIs = [0.16,0.34] | 0.25 CIs = [0.16,0.35] | 0.44 CIs = [0.35,0.55] | 0.48 CIs = [0.37,0.57] | 0.17 CIs = [0.10,0.24] | 0.43 CIs = [0.34,0.52] | 0.17 CIs = [0.07,0.26] |
| cardiologist 8 | 0.42 CIs = [0.35,0.49] | 0.46 CIs = [0.38,0.53] | 0.51 CIs = [0.42,0.60] | 0.47 CIs = [0.39,0.55] | 0.68 CIs = [0.59,0.76] | 0.53 CIs = [0.44,0.61] | 0.44 CIs = [0.35,0.52] | 0.62 CIs = [0.53,0.69] | 0.78 CIs = [0.70,0.85] | 0.66 CIs = [0.58,0.75] | 1.00 CIs = [1.00,1.00] | 0.40 CIs = [0.33,0.48] | 0.40 CIs = [0.34,0.47] | 0.13 CIs = [0.04,0.21] | 0.25 CIs = [0.15,0.35] | 0.40 CIs = [0.29,0.51] | 0.48 CIs = [0.37,0.57] | 0.19 CIs = [0.13,0.26] | 0.38 CIs = [0.28,0.46] | 0.11 CIs = [0.02,0.21] |
| cardiologist 9 | 0.54 CIs = [0.47,0.60] | 0.49 CIs = [0.41,0.56] | 0.47 CIs = [0.40,0.55] | 0.44 CIs = [0.37,0.51] | 0.40 CIs = [0.33,0.48] | 0.51 CIs = [0.44,0.58] | 0.42 CIs = [0.34,0.50] | 0.56 CIs = [0.49,0.64] | 0.45 CIs = [0.38,0.52] | 0.38 CIs = [0.31,0.46] | 0.40 CIs = [0.33,0.47] | 1.00 CIs = [1.00,1.00] | 0.82 CIs = [0.77,0.86] | 0.26 CIs = [0.18,0.34] | 0.18 CIs = [0.11,0.26] | 0.26 CIs = [0.19,0.34] | 0.29 CIs = [0.21,0.36] | 0.36 CIs = [0.29,0.43] | 0.42 CIs = [0.35,0.50] | 0.11 CIs = [0.04,0.18] |
| cardiologist 10 | 0.61 CIs = [0.54,0.67] | 0.55 CIs = [0.47,0.61] | 0.57 CIs = [0.51,0.64] | 0.52 CIs = [0.46,0.60] | 0.46 CIs = [0.39,0.53] | 0.59 CIs = [0.52,0.65] | 0.48 CIs = [0.41,0.54] | 0.57 CIs = [0.50,0.64] | 0.46 CIs = [0.39,0.53] | 0.44 CIs = [0.37,0.51] | 0.40 CIs = [0.33,0.46] | 0.82 CIs = [0.77,0.86] | 1.00 CIs = [1.00,1.00] | 0.31 CIs = [0.24,0.39] | 0.23 CIs = [0.16,0.30] | 0.26 CIs = [0.19,0.33] | 0.33 CIs = [0.26,0.39] | 0.38 CIs = [0.31,0.44] | 0.41 CIs = [0.34,0.49] | 0.17 CIs = [0.10,0.23] |
| cardiologist 11 | 0.35 CIs = [0.27,0.43] | 0.37 CIs = [0.29,0.44] | 0.37 CIs = [0.29,0.46] | 0.31 CIs = [0.22,0.39] | 0.24 CIs = [0.15,0.33] | 0.39 CIs = [0.30,0.47] | 0.39 CIs = [0.30,0.47] | 0.29 CIs = [0.20,0.38] | 0.24 CIs = [0.15,0.34] | 0.25 CIs = [0.16,0.34] | 0.13 CIs = [0.04,0.22] | 0.26 CIs = [0.19,0.34] | 0.32 CIs = [0.24,0.39] | 1.00 CIs = [1.00,1.00] | 0.28 CIs = [0.19,0.38] | 0.19 CIs = [0.10,0.29] | 0.12 CIs = [0.04,0.21] | 0.17 CIs = [0.10,0.23] | 0.23 CIs = [0.14,0.31] | 0.25 CIs = [0.15,0.35] |
| cardiologist 12 | 0.24 CIs = [0.16,0.32] | 0.22 CIs = [0.15,0.30] | 0.28 CIs = [0.19,0.36] | 0.25 CIs = [0.17,0.33] | 0.25 CIs = [0.16,0.35] | 0.29 CIs = [0.20,0.37] | 0.31 CIs = [0.22,0.40] | 0.27 CIs = [0.18,0.36] | 0.31 CIs = [0.21,0.41] | 0.25 CIs = [0.15,0.35] | 0.25 CIs = [0.15,0.35] | 0.18 CIs = [0.11,0.25] | 0.23 CIs = [0.17,0.30] | 0.28 CIs = [0.18,0.38] | 1.00 CIs = [1.00,1.00] | 0.22 CIs = [0.12,0.32] | 0.26 CIs = [0.15,0.37] | 0.10 CIs = [0.03,0.16] | 0.21 CIs = [0.13,0.30] | 0.74 CIs = [0.65,0.82] |
| cardiologist 13 | 0.26 CIs = [0.19,0.34] | 0.23 CIs = [0.15,0.31] | 0.29 CIs = [0.20,0.37] | 0.25 CIs = [0.16,0.33] | 0.45 CIs = [0.35,0.54] | 0.36 CIs = [0.27,0.44] | 0.23 CIs = [0.14,0.32] | 0.42 CIs = [0.33,0.52] | 0.42 CIs = [0.31,0.51] | 0.45 CIs = [0.35,0.54] | 0.40 CIs = [0.29,0.50] | 0.27 CIs = [0.19,0.34] | 0.26 CIs = [0.20,0.33] | 0.19 CIs = [0.10,0.28] | 0.22 CIs = [0.12,0.32] | 1.00 CIs = [1.00,1.00] | 0.45 CIs = [0.33,0.54] | 0.14 CIs = [0.07,0.21] | 0.48 CIs = [0.38,0.56] | 0.26 CIs = [0.15,0.36] |
| cardiologist 14 | 0.36 CIs = [0.29,0.44] | 0.33 CIs = [0.25,0.41] | 0.41 CIs = [0.33,0.50] | 0.37 CIs = [0.28,0.46] | 0.46 CIs = [0.37,0.56] | 0.38 CIs = [0.30,0.46] | 0.40 CIs = [0.30,0.49] | 0.41 CIs = [0.33,0.49] | 0.49 CIs = [0.39,0.58] | 0.47 CIs = [0.38,0.57] | 0.48 CIs = [0.37,0.58] | 0.29 CIs = [0.21,0.36] | 0.33 CIs = [0.25,0.39] | 0.12 CIs = [0.04,0.21] | 0.26 CIs = [0.16,0.37] | 0.44 CIs = [0.34,0.55] | 1.00 CIs = [1.00,1.00] | 0.17 CIs = [0.11,0.23] | 0.31 CIs = [0.22,0.40] | 0.17 CIs = [0.06,0.27] |
| cardiologist 15 | 0.27 CIs = [0.19,0.34] | 0.24 CIs = [0.17,0.32] | 0.22 CIs = [0.15,0.29] | 0.21 CIs = [0.14,0.28] | 0.19 CIs = [0.12,0.26] | 0.26 CIs = [0.19,0.34] | 0.22 CIs = [0.15,0.30] | 0.34 CIs = [0.28,0.42] | 0.22 CIs = [0.14,0.28] | 0.17 CIs = [0.10,0.24] | 0.19 CIs = [0.12,0.26] | 0.36 CIs = [0.29,0.44] | 0.38 CIs = [0.30,0.45] | 0.17 CIs = [0.10,0.24] | 0.10 CIs = [0.04,0.16] | 0.14 CIs = [0.08,0.21] | 0.17 CIs = [0.10,0.24] | 1.00 CIs = [1.00,1.00] | 0.24 CIs = [0.17,0.32] | 0.10 CIs = [0.03,0.16] |
| cardiologist 16 | 0.36 CIs = [0.28,0.43] | 0.32 CIs = [0.24,0.40] | 0.35 CIs = [0.27,0.43] | 0.31 CIs = [0.23,0.39] | 0.45 CIs = [0.36,0.53] | 0.41 CIs = [0.32,0.48] | 0.29 CIs = [0.21,0.38] | 0.51 CIs = [0.42,0.58] | 0.36 CIs = [0.27,0.45] | 0.43 CIs = [0.34,0.52] | 0.38 CIs = [0.28,0.47] | 0.42 CIs = [0.35,0.50] | 0.41 CIs = [0.34,0.48] | 0.23 CIs = [0.14,0.31] | 0.22 CIs = [0.12,0.30] | 0.47 CIs = [0.38,0.57] | 0.31 CIs = [0.22,0.41] | 0.24 CIs = [0.17,0.31] | 1.00 CIs = [1.00,1.00] | 0.23 CIs = [0.14,0.33] |
| cardiologist 17 | 0.18 CIs = [0.12,0.25] | 0.19 CIs = [0.12,0.26] | 0.22 CIs = [0.14,0.31] | 0.20 CIs = [0.11,0.29] | 0.18 CIs = [0.09,0.27] | 0.27 CIs = [0.18,0.34] | 0.22 CIs = [0.14,0.31] | 0.23 CIs = [0.15,0.31] | 0.26 CIs = [0.16,0.35] | 0.17 CIs = [0.07,0.26] | 0.11 CIs = [0.02,0.21] | 0.11 CIs = [0.04,0.18] | 0.17 CIs = [0.10,0.24] | 0.25 CIs = [0.15,0.35] | 0.74 CIs = [0.65,0.82] | 0.25 CIs = [0.16,0.36] | 0.17 CIs = [0.07,0.27] | 0.09 CIs = [0.04,0.16] | 0.23 CIs = [0.14,0.32] | 1.00 CIs = [1.00,1.00] |

**Table S5d. Inter-cardiologists and AIEchoDx model variability when diagnosing HCM and non-HCM patients estimated with the Cohen’s Kappa statistic**

|  | Ground-true | AIEchoDx | Consensus | cardiologist 1 | cardiologist 2 | cardiologist 3 | cardiologist 4 | cardiologist 5 | cardiologist 6 | cardiologist 7 | cardiologist 8 | cardiologist 9 | cardiologist 10 | cardiologist 11 | cardiologist 12 | cardiologist 13 | cardiologist 14 | cardiologist 15 | cardiologist 16 | cardiologist 17 |
| --- | --- | --- | --- | --- | --- | --- | --- | --- | --- | --- | --- | --- | --- | --- | --- | --- | --- | --- | --- | --- |
| Ground-true | 1.00 CIs = [1.00,1.00] | 0.88 CIs = [0.81,0.94] | 0.82 CIs = [0.74,0.89] | 0.69 CIs = [0.58,0.78] | 0.76 CIs = [0.68,0.84] | 0.67 CIs = [0.58,0.75] | 0.76 CIs = [0.66,0.83] | 0.48 CIs = [0.40,0.58] | 0.71 CIs = [0.61,0.79] | 0.76 CIs = [0.68,0.83] | 0.77 CIs = [0.69,0.85] | 0.56 CIs = [0.46,0.65] | 0.56 CIs = [0.46,0.65] | 0.61 CIs = [0.52,0.70] | 0.37 CIs = [0.29,0.45] | 0.67 CIs = [0.58,0.75] | 0.37 CIs = [0.26,0.48] | 0.52 CIs = [0.43,0.62] | 0.21 CIs = [0.16,0.27] | 0.34 CIs = [0.26,0.42] |
| AIEchoDx | 0.88 CIs = [0.81,0.94] | 1.00 CIs = [1.00,1.00] | 0.78 CIs = [0.69,0.86] | 0.62 CIs = [0.51,0.73] | 0.75 CIs = [0.67,0.83] | 0.64 CIs = [0.55,0.72] | 0.72 CIs = [0.64,0.80] | 0.46 CIs = [0.38,0.54] | 0.65 CIs = [0.55,0.74] | 0.74 CIs = [0.66,0.82] | 0.71 CIs = [0.62,0.80] | 0.48 CIs = [0.37,0.58] | 0.50 CIs = [0.40,0.60] | 0.56 CIs = [0.47,0.65] | 0.38 CIs = [0.30,0.46] | 0.62 CIs = [0.53,0.71] | 0.35 CIs = [0.25,0.45] | 0.48 CIs = [0.38,0.58] | 0.20 CIs = [0.14,0.26] | 0.34 CIs = [0.26,0.41] |
| Consensus | 0.82 CIs = [0.74,0.89] | 0.78 CIs = [0.69,0.85] | 1.00 CIs = [1.00,1.00] | 0.77 CIs = [0.68,0.85] | 0.83 CIs = [0.76,0.89] | 0.75 CIs = [0.67,0.82] | 0.86 CIs = [0.80,0.92] | 0.54 CIs = [0.46,0.62] | 0.77 CIs = [0.69,0.85] | 0.82 CIs = [0.74,0.88] | 0.85 CIs = [0.78,0.91] | 0.61 CIs = [0.52,0.70] | 0.61 CIs = [0.51,0.70] | 0.61 CIs = [0.52,0.71] | 0.40 CIs = [0.32,0.48] | 0.68 CIs = [0.58,0.76] | 0.43 CIs = [0.32,0.53] | 0.49 CIs = [0.40,0.58] | 0.24 CIs = [0.18,0.30] | 0.36 CIs = [0.28,0.44] |
| cardiologist 1 | 0.68 CIs = [0.57,0.79] | 0.62 CIs = [0.50,0.72] | 0.77 CIs = [0.68,0.85] | 1.00 CIs = [1.00,1.00] | 0.67 CIs = [0.57,0.76] | 0.55 CIs = [0.45,0.64] | 0.64 CIs = [0.54,0.73] | 0.40 CIs = [0.31,0.49] | 0.68 CIs = [0.57,0.77] | 0.66 CIs = [0.56,0.76] | 0.78 CIs = [0.69,0.86] | 0.47 CIs = [0.36,0.57] | 0.46 CIs = [0.36,0.56] | 0.48 CIs = [0.38,0.58] | 0.29 CIs = [0.21,0.37] | 0.55 CIs = [0.44,0.64] | 0.34 CIs = [0.24,0.45] | 0.43 CIs = [0.33,0.53] | 0.20 CIs = [0.14,0.25] | 0.29 CIs = [0.22,0.37] |
| cardiologist 2 | 0.76 CIs = [0.67,0.84] | 0.75 CIs = [0.66,0.83] | 0.83 CIs = [0.75,0.89] | 0.67 CIs = [0.57,0.76] | 1.00 CIs = [1.00,1.00] | 0.71 CIs = [0.63,0.78] | 0.81 CIs = [0.74,0.88] | 0.55 CIs = [0.46,0.63] | 0.71 CIs = [0.62,0.78] | 0.99 CIs = [0.97,1.00] | 0.75 CIs = [0.67,0.82] | 0.54 CIs = [0.44,0.63] | 0.54 CIs = [0.44,0.63] | 0.56 CIs = [0.47,0.64] | 0.39 CIs = [0.31,0.46] | 0.69 CIs = [0.61,0.76] | 0.44 CIs = [0.34,0.54] | 0.55 CIs = [0.47,0.64] | 0.26 CIs = [0.20,0.32] | 0.36 CIs = [0.29,0.44] |
| cardiologist 3 | 0.67 CIs = [0.59,0.75] | 0.64 CIs = [0.55,0.73] | 0.75 CIs = [0.67,0.82] | 0.54 CIs = [0.45,0.64] | 0.71 CIs = [0.62,0.78] | 1.00 CIs = [1.00,1.00] | 0.65 CIs = [0.55,0.72] | 0.66 CIs = [0.59,0.73] | 0.63 CIs = [0.54,0.71] | 0.70 CIs = [0.62,0.78] | 0.68 CIs = [0.59,0.75] | 0.55 CIs = [0.46,0.64] | 0.56 CIs = [0.47,0.65] | 0.63 CIs = [0.55,0.70] | 0.45 CIs = [0.38,0.53] | 0.70 CIs = [0.62,0.77] | 0.38 CIs = [0.28,0.47] | 0.57 CIs = [0.48,0.65] | 0.31 CIs = [0.25,0.38] | 0.44 CIs = [0.36,0.52] |
| cardiologist 4 | 0.75 CIs = [0.67,0.84] | 0.72 CIs = [0.63,0.81] | 0.86 CIs = [0.80,0.92] | 0.64 CIs = [0.53,0.73] | 0.81 CIs = [0.74,0.88] | 0.65 CIs = [0.56,0.73] | 1.00 CIs = [1.00,1.00] | 0.53 CIs = [0.45,0.61] | 0.68 CIs = [0.59,0.77] | 0.80 CIs = [0.73,0.87] | 0.72 CIs = [0.63,0.80] | 0.57 CIs = [0.47,0.66] | 0.58 CIs = [0.49,0.68] | 0.56 CIs = [0.47,0.65] | 0.36 CIs = [0.27,0.44] | 0.63 CIs = [0.54,0.72] | 0.42 CIs = [0.32,0.52] | 0.48 CIs = [0.38,0.57] | 0.24 CIs = [0.18,0.30] | 0.33 CIs = [0.25,0.41] |
| cardiologist 5 | 0.49 CIs = [0.40,0.56] | 0.47 CIs = [0.38,0.54] | 0.54 CIs = [0.46,0.62] | 0.40 CIs = [0.31,0.48] | 0.55 CIs = [0.47,0.62] | 0.66 CIs = [0.59,0.73] | 0.53 CIs = [0.45,0.61] | 1.00 CIs = [1.00,1.00] | 0.49 CIs = [0.41,0.58] | 0.54 CIs = [0.46,0.62] | 0.52 CIs = [0.44,0.60] | 0.45 CIs = [0.36,0.52] | 0.44 CIs = [0.36,0.52] | 0.55 CIs = [0.47,0.63] | 0.55 CIs = [0.49,0.63] | 0.59 CIs = [0.51,0.66] | 0.32 CIs = [0.24,0.40] | 0.52 CIs = [0.44,0.59] | 0.41 CIs = [0.35,0.48] | 0.52 CIs = [0.44,0.59] |
| cardiologist 6 | 0.71 CIs = [0.61,0.79] | 0.65 CIs = [0.55,0.74] | 0.77 CIs = [0.69,0.84] | 0.68 CIs = [0.58,0.77] | 0.71 CIs = [0.62,0.79] | 0.63 CIs = [0.53,0.71] | 0.68 CIs = [0.59,0.76] | 0.50 CIs = [0.41,0.58] | 1.00 CIs = [1.00,1.00] | 0.70 CIs = [0.61,0.78] | 0.89 CIs = [0.83,0.94] | 0.50 CIs = [0.41,0.60] | 0.51 CIs = [0.41,0.60] | 0.51 CIs = [0.42,0.61] | 0.39 CIs = [0.32,0.47] | 0.63 CIs = [0.54,0.72] | 0.43 CIs = [0.33,0.52] | 0.47 CIs = [0.37,0.56] | 0.24 CIs = [0.17,0.30] | 0.38 CIs = [0.30,0.46] |
| cardiologist 7 | 0.76 CIs = [0.67,0.84] | 0.74 CIs = [0.66,0.83] | 0.82 CIs = [0.75,0.89] | 0.66 CIs = [0.56,0.76] | 0.99 CIs = [0.97,1.00] | 0.70 CIs = [0.62,0.77] | 0.80 CIs = [0.73,0.87] | 0.54 CIs = [0.46,0.61] | 0.70 CIs = [0.61,0.78] | 1.00 CIs = [1.00,1.00] | 0.74 CIs = [0.65,0.82] | 0.53 CIs = [0.43,0.63] | 0.53 CIs = [0.43,0.62] | 0.56 CIs = [0.47,0.64] | 0.39 CIs = [0.31,0.47] | 0.69 CIs = [0.60,0.76] | 0.43 CIs = [0.33,0.53] | 0.54 CIs = [0.45,0.63] | 0.26 CIs = [0.20,0.32] | 0.36 CIs = [0.28,0.44] |
| cardiologist 8 | 0.78 CIs = [0.68,0.85] | 0.71 CIs = [0.62,0.80] | 0.85 CIs = [0.77,0.91] | 0.78 CIs = [0.69,0.86] | 0.75 CIs = [0.66,0.82] | 0.67 CIs = [0.59,0.76] | 0.72 CIs = [0.63,0.80] | 0.52 CIs = [0.43,0.60] | 0.89 CIs = [0.83,0.94] | 0.74 CIs = [0.65,0.82] | 1.00 CIs = [1.00,1.00] | 0.55 CIs = [0.45,0.64] | 0.55 CIs = [0.45,0.64] | 0.56 CIs = [0.46,0.65] | 0.39 CIs = [0.31,0.46] | 0.66 CIs = [0.56,0.74] | 0.42 CIs = [0.32,0.52] | 0.49 CIs = [0.40,0.59] | 0.25 CIs = [0.19,0.30] | 0.36 CIs = [0.28,0.44] |
| cardiologist 9 | 0.56 CIs = [0.46,0.66] | 0.48 CIs = [0.37,0.59] | 0.61 CIs = [0.52,0.70] | 0.47 CIs = [0.36,0.57] | 0.54 CIs = [0.43,0.64] | 0.55 CIs = [0.45,0.63] | 0.57 CIs = [0.47,0.66] | 0.44 CIs = [0.36,0.52] | 0.51 CIs = [0.40,0.60] | 0.53 CIs = [0.43,0.62] | 0.55 CIs = [0.45,0.64] | 1.00 CIs = [1.00,1.00] | 0.83 CIs = [0.77,0.89] | 0.51 CIs = [0.41,0.61] | 0.37 CIs = [0.30,0.45] | 0.58 CIs = [0.50,0.66] | 0.38 CIs = [0.28,0.47] | 0.44 CIs = [0.35,0.53] | 0.29 CIs = [0.23,0.36] | 0.38 CIs = [0.30,0.45] |
| cardiologist 10 | 0.56 CIs = [0.46,0.66] | 0.50 CIs = [0.39,0.59] | 0.61 CIs = [0.52,0.70] | 0.46 CIs = [0.35,0.57] | 0.54 CIs = [0.44,0.63] | 0.56 CIs = [0.47,0.65] | 0.59 CIs = [0.48,0.68] | 0.44 CIs = [0.36,0.53] | 0.51 CIs = [0.41,0.60] | 0.53 CIs = [0.43,0.63] | 0.55 CIs = [0.44,0.64] | 0.83 CIs = [0.77,0.89] | 1.00 CIs = [1.00,1.00] | 0.55 CIs = [0.46,0.64] | 0.38 CIs = [0.30,0.46] | 0.56 CIs = [0.47,0.64] | 0.36 CIs = [0.25,0.44] | 0.45 CIs = [0.36,0.54] | 0.32 CIs = [0.26,0.39] | 0.39 CIs = [0.30,0.46] |
| cardiologist 11 | 0.61 CIs = [0.52,0.70] | 0.56 CIs = [0.47,0.65] | 0.61 CIs = [0.52,0.69] | 0.48 CIs = [0.38,0.57] | 0.56 CIs = [0.46,0.65] | 0.63 CIs = [0.55,0.71] | 0.56 CIs = [0.46,0.64] | 0.55 CIs = [0.47,0.63] | 0.52 CIs = [0.42,0.60] | 0.55 CIs = [0.46,0.64] | 0.56 CIs = [0.47,0.64] | 0.51 CIs = [0.42,0.60] | 0.56 CIs = [0.46,0.64] | 1.00 CIs = [1.00,1.00] | 0.46 CIs = [0.38,0.54] | 0.66 CIs = [0.57,0.73] | 0.37 CIs = [0.27,0.46] | 0.61 CIs = [0.53,0.69] | 0.34 CIs = [0.28,0.40] | 0.44 CIs = [0.37,0.52] |
| cardiologist 12 | 0.37 CIs = [0.30,0.45] | 0.38 CIs = [0.30,0.45] | 0.40 CIs = [0.33,0.47] | 0.29 CIs = [0.21,0.36] | 0.39 CIs = [0.31,0.46] | 0.45 CIs = [0.38,0.52] | 0.36 CIs = [0.28,0.44] | 0.56 CIs = [0.48,0.63] | 0.39 CIs = [0.31,0.47] | 0.39 CIs = [0.30,0.46] | 0.39 CIs = [0.31,0.47] | 0.37 CIs = [0.29,0.46] | 0.38 CIs = [0.31,0.46] | 0.46 CIs = [0.38,0.53] | 1.00 CIs = [1.00,1.00] | 0.51 CIs = [0.44,0.59] | 0.31 CIs = [0.23,0.39] | 0.47 CIs = [0.40,0.55] | 0.44 CIs = [0.38,0.51] | 0.93 CIs = [0.90,0.96] |
| cardiologist 13 | 0.67 CIs = [0.59,0.75] | 0.63 CIs = [0.54,0.71] | 0.67 CIs = [0.59,0.75] | 0.54 CIs = [0.45,0.64] | 0.69 CIs = [0.61,0.77] | 0.70 CIs = [0.62,0.77] | 0.63 CIs = [0.54,0.72] | 0.58 CIs = [0.51,0.66] | 0.63 CIs = [0.54,0.71] | 0.68 CIs = [0.60,0.77] | 0.66 CIs = [0.57,0.75] | 0.58 CIs = [0.49,0.67] | 0.56 CIs = [0.47,0.65] | 0.66 CIs = [0.57,0.74] | 0.51 CIs = [0.44,0.59] | 1.00 CIs = [1.00,1.00] | 0.49 CIs = [0.40,0.58] | 0.63 CIs = [0.55,0.71] | 0.29 CIs = [0.22,0.35] | 0.52 CIs = [0.45,0.59] |
| cardiologist 14 | 0.37 CIs = [0.26,0.47] | 0.35 CIs = [0.24,0.44] | 0.43 CIs = [0.32,0.53] | 0.34 CIs = [0.23,0.45] | 0.44 CIs = [0.34,0.54] | 0.38 CIs = [0.28,0.47] | 0.42 CIs = [0.31,0.51] | 0.32 CIs = [0.24,0.41] | 0.43 CIs = [0.33,0.52] | 0.44 CIs = [0.33,0.53] | 0.42 CIs = [0.32,0.53] | 0.38 CIs = [0.28,0.47] | 0.35 CIs = [0.26,0.46] | 0.37 CIs = [0.28,0.47] | 0.31 CIs = [0.24,0.40] | 0.49 CIs = [0.40,0.58] | 1.00 CIs = [1.00,1.00] | 0.32 CIs = [0.22,0.42] | 0.17 CIs = [0.11,0.23] | 0.31 CIs = [0.23,0.39] |
| cardiologist 15 | 0.52 CIs = [0.43,0.61] | 0.48 CIs = [0.38,0.56] | 0.50 CIs = [0.40,0.58] | 0.43 CIs = [0.34,0.53] | 0.55 CIs = [0.46,0.64] | 0.57 CIs = [0.48,0.65] | 0.48 CIs = [0.39,0.57] | 0.52 CIs = [0.44,0.60] | 0.47 CIs = [0.38,0.56] | 0.54 CIs = [0.46,0.63] | 0.50 CIs = [0.40,0.59] | 0.44 CIs = [0.35,0.54] | 0.45 CIs = [0.36,0.54] | 0.61 CIs = [0.53,0.68] | 0.48 CIs = [0.40,0.55] | 0.63 CIs = [0.55,0.71] | 0.32 CIs = [0.23,0.41] | 1.00 CIs = [1.00,1.00] | 0.36 CIs = [0.30,0.42] | 0.49 CIs = [0.42,0.57] |
| cardiologist 16 | 0.22 CIs = [0.16,0.27] | 0.20 CIs = [0.14,0.26] | 0.24 CIs = [0.19,0.30] | 0.19 CIs = [0.15,0.25] | 0.26 CIs = [0.20,0.32] | 0.32 CIs = [0.25,0.38] | 0.24 CIs = [0.18,0.30] | 0.41 CIs = [0.35,0.48] | 0.24 CIs = [0.18,0.29] | 0.26 CIs = [0.20,0.32] | 0.24 CIs = [0.19,0.30] | 0.29 CIs = [0.23,0.36] | 0.32 CIs = [0.26,0.38] | 0.34 CIs = [0.28,0.40] | 0.45 CIs = [0.38,0.51] | 0.29 CIs = [0.23,0.35] | 0.17 CIs = [0.11,0.23] | 0.36 CIs = [0.30,0.43] | 1.00 CIs = [1.00,1.00] | 0.43 CIs = [0.36,0.49] |
| cardiologist 17 | 0.34 CIs = [0.27,0.42] | 0.34 CIs = [0.26,0.42] | 0.36 CIs = [0.28,0.44] | 0.29 CIs = [0.22,0.37] | 0.37 CIs = [0.29,0.45] | 0.44 CIs = [0.36,0.51] | 0.33 CIs = [0.25,0.41] | 0.52 CIs = [0.45,0.59] | 0.38 CIs = [0.30,0.46] | 0.36 CIs = [0.28,0.44] | 0.36 CIs = [0.29,0.43] | 0.38 CIs = [0.30,0.45] | 0.38 CIs = [0.31,0.46] | 0.44 CIs = [0.37,0.52] | 0.93 CIs = [0.90,0.96] | 0.52 CIs = [0.44,0.60] | 0.31 CIs = [0.23,0.39] | 0.50 CIs = [0.42,0.56] | 0.43 CIs = [0.36,0.50] | 1.00 CIs = [1.00,1.00] |

**Table S5e. Inter-cardiologists and AIEchoDx model variability when diagnosing prior MI and non-prior MI patients estimated with the Cohen’s Kappa statistic**

|  | Ground-true | AIEchoDx | Consensus | cardiologist 1 | cardiologist 2 | cardiologist 3 | cardiologist 4 | cardiologist 5 | cardiologist 6 | cardiologist 7 | cardiologist 8 | cardiologist 9 | cardiologist 10 | cardiologist 11 | cardiologist 12 | cardiologist 13 | cardiologist 14 | cardiologist 15 | cardiologist 16 | cardiologist 17 |
| --- | --- | --- | --- | --- | --- | --- | --- | --- | --- | --- | --- | --- | --- | --- | --- | --- | --- | --- | --- | --- |
| Ground-true | 1.00 CIs = [1.00,1.00] | 0.87 CIs = [0.83,0.90] | 0.65 CIs = [0.60,0.71] | 0.57 CIs = [0.51,0.63] | 0.40 CIs = [0.34,0.47] | 0.59 CIs = [0.53,0.64] | 0.64 CIs = [0.59,0.69] | 0.45 CIs = [0.38,0.51] | 0.38 CIs = [0.31,0.45] | 0.37 CIs = [0.31,0.44] | 0.32 CIs = [0.26,0.39] | 0.39 CIs = [0.33,0.46] | 0.44 CIs = [0.37,0.50] | -0.01 CIs = [-0.08,0.05] | 0.18 CIs = [0.12,0.25] | 0.30 CIs = [0.23,0.36] | 0.36 CIs = [0.30,0.42] | 0.23 CIs = [0.16,0.30] | 0.03 CIs = [-0.03,0.11] | 0.19 CIs = [0.11,0.26] |
| AIEchoDx | 0.87 CIs = [0.83,0.90] | 1.00 CIs = [1.00,1.00] | 0.55 CIs = [0.48,0.61] | 0.45 CIs = [0.38,0.52] | 0.33 CIs = [0.26,0.40] | 0.53 CIs = [0.48,0.60] | 0.55 CIs = [0.49,0.61] | 0.34 CIs = [0.27,0.41] | 0.30 CIs = [0.23,0.37] | 0.31 CIs = [0.24,0.38] | 0.26 CIs = [0.19,0.32] | 0.36 CIs = [0.29,0.43] | 0.39 CIs = [0.32,0.45] | -0.03 CIs = [-0.09,0.03] | 0.14 CIs = [0.07,0.22] | 0.26 CIs = [0.18,0.32] | 0.30 CIs = [0.25,0.36] | 0.18 CIs = [0.11,0.25] | 0.05 CIs = [-0.02,0.11] | 0.15 CIs = [0.08,0.21] |
| Consensus | 0.65 CIs = [0.60,0.71] | 0.55 CIs = [0.48,0.61] | 1.00 CIs = [1.00,1.00] | 0.82 CIs = [0.77,0.86] | 0.56 CIs = [0.50,0.62] | 0.77 CIs = [0.72,0.82] | 0.63 CIs = [0.58,0.69] | 0.55 CIs = [0.49,0.61] | 0.58 CIs = [0.52,0.64] | 0.54 CIs = [0.47,0.60] | 0.53 CIs = [0.47,0.59] | 0.29 CIs = [0.23,0.36] | 0.30 CIs = [0.23,0.37] | 0.10 CIs = [0.03,0.17] | 0.25 CIs = [0.19,0.32] | 0.39 CIs = [0.33,0.46] | 0.30 CIs = [0.24,0.35] | 0.13 CIs = [0.06,0.20] | 0.11 CIs = [0.04,0.18] | 0.27 CIs = [0.21,0.34] |
| cardiologist 1 | 0.57 CIs = [0.51,0.63] | 0.45 CIs = [0.38,0.52] | 0.82 CIs = [0.78,0.86] | 1.00 CIs = [1.00,1.00] | 0.51 CIs = [0.45,0.58] | 0.59 CIs = [0.52,0.65] | 0.48 CIs = [0.42,0.54] | 0.53 CIs = [0.46,0.59] | 0.56 CIs = [0.50,0.62] | 0.50 CIs = [0.43,0.56] | 0.52 CIs = [0.46,0.58] | 0.21 CIs = [0.13,0.28] | 0.21 CIs = [0.14,0.29] | 0.08 CIs = [0.01,0.15] | 0.24 CIs = [0.17,0.31] | 0.37 CIs = [0.30,0.43] | 0.25 CIs = [0.19,0.31] | 0.07 CIs = [0.00,0.14] | 0.16 CIs = [0.09,0.23] | 0.27 CIs = [0.20,0.33] |
| cardiologist 2 | 0.40 CIs = [0.33,0.46] | 0.33 CIs = [0.26,0.39] | 0.56 CIs = [0.50,0.62] | 0.51 CIs = [0.45,0.57] | 1.00 CIs = [1.00,1.00] | 0.59 CIs = [0.53,0.65] | 0.40 CIs = [0.33,0.46] | 0.54 CIs = [0.47,0.60] | 0.65 CIs = [0.59,0.70] | 0.97 CIs = [0.95,0.99] | 0.63 CIs = [0.58,0.69] | 0.23 CIs = [0.17,0.30] | 0.25 CIs = [0.18,0.31] | 0.09 CIs = [0.03,0.16] | 0.33 CIs = [0.26,0.40] | 0.49 CIs = [0.43,0.55] | 0.25 CIs = [0.19,0.31] | 0.14 CIs = [0.06,0.21] | 0.27 CIs = [0.20,0.33] | 0.32 CIs = [0.25,0.39] |
| cardiologist 3 | 0.59 CIs = [0.53,0.64] | 0.54 CIs = [0.46,0.60] | 0.77 CIs = [0.72,0.82] | 0.59 CIs = [0.52,0.65] | 0.59 CIs = [0.53,0.65] | 1.00 CIs = [1.00,1.00] | 0.44 CIs = [0.38,0.49] | 0.62 CIs = [0.57,0.69] | 0.59 CIs = [0.52,0.65] | 0.57 CIs = [0.51,0.62] | 0.52 CIs = [0.46,0.58] | 0.37 CIs = [0.30,0.44] | 0.36 CIs = [0.29,0.42] | 0.06 CIs = [-0.02,0.13] | 0.29 CIs = [0.22,0.36] | 0.42 CIs = [0.36,0.47] | 0.25 CIs = [0.20,0.30] | 0.16 CIs = [0.09,0.23] | 0.14 CIs = [0.07,0.22] | 0.32 CIs = [0.25,0.39] |
| cardiologist 4 | 0.64 CIs = [0.59,0.69] | 0.55 CIs = [0.49,0.61] | 0.63 CIs = [0.58,0.69] | 0.48 CIs = [0.42,0.54] | 0.40 CIs = [0.33,0.46] | 0.44 CIs = [0.37,0.49] | 1.00 CIs = [1.00,1.00] | 0.32 CIs = [0.26,0.38] | 0.36 CIs = [0.30,0.43] | 0.40 CIs = [0.34,0.47] | 0.34 CIs = [0.27,0.40] | 0.32 CIs = [0.26,0.39] | 0.34 CIs = [0.28,0.41] | 0.07 CIs = [0.01,0.13] | 0.21 CIs = [0.14,0.28] | 0.33 CIs = [0.26,0.39] | 0.35 CIs = [0.29,0.41] | 0.20 CIs = [0.14,0.27] | 0.06 CIs = [-0.01,0.11] | 0.21 CIs = [0.13,0.27] |
| cardiologist 5 | 0.45 CIs = [0.38,0.51] | 0.34 CIs = [0.27,0.41] | 0.55 CIs = [0.49,0.61] | 0.53 CIs = [0.46,0.59] | 0.54 CIs = [0.47,0.60] | 0.63 CIs = [0.56,0.68] | 0.32 CIs = [0.26,0.39] | 1.00 CIs = [1.00,1.00] | 0.61 CIs = [0.55,0.67] | 0.52 CIs = [0.46,0.57] | 0.53 CIs = [0.47,0.59] | 0.31 CIs = [0.24,0.38] | 0.31 CIs = [0.24,0.38] | 0.06 CIs = [-0.01,0.13] | 0.28 CIs = [0.21,0.35] | 0.41 CIs = [0.35,0.47] | 0.22 CIs = [0.17,0.28] | 0.19 CIs = [0.12,0.26] | 0.19 CIs = [0.12,0.27] | 0.30 CIs = [0.23,0.36] |
| cardiologist 6 | 0.38 CIs = [0.32,0.45] | 0.30 CIs = [0.23,0.37] | 0.58 CIs = [0.52,0.63] | 0.56 CIs = [0.50,0.62] | 0.65 CIs = [0.59,0.71] | 0.58 CIs = [0.52,0.65] | 0.36 CIs = [0.29,0.43] | 0.61 CIs = [0.56,0.67] | 1.00 CIs = [1.00,1.00] | 0.65 CIs = [0.59,0.70] | 0.77 CIs = [0.71,0.81] | 0.23 CIs = [0.16,0.30] | 0.23 CIs = [0.16,0.30] | 0.12 CIs = [0.05,0.18] | 0.31 CIs = [0.25,0.38] | 0.46 CIs = [0.41,0.53] | 0.32 CIs = [0.26,0.38] | 0.15 CIs = [0.08,0.22] | 0.19 CIs = [0.12,0.26] | 0.30 CIs = [0.23,0.37] |
| cardiologist 7 | 0.38 CIs = [0.30,0.45] | 0.31 CIs = [0.24,0.38] | 0.54 CIs = [0.48,0.60] | 0.50 CIs = [0.43,0.56] | 0.97 CIs = [0.95,0.99] | 0.57 CIs = [0.51,0.62] | 0.40 CIs = [0.34,0.47] | 0.52 CIs = [0.45,0.58] | 0.65 CIs = [0.59,0.70] | 1.00 CIs = [1.00,1.00] | 0.62 CIs = [0.56,0.67] | 0.21 CIs = [0.14,0.28] | 0.22 CIs = [0.15,0.30] | 0.10 CIs = [0.04,0.17] | 0.32 CIs = [0.25,0.39] | 0.48 CIs = [0.42,0.55] | 0.25 CIs = [0.18,0.31] | 0.13 CIs = [0.06,0.20] | 0.24 CIs = [0.18,0.31] | 0.31 CIs = [0.24,0.38] |
| cardiologist 8 | 0.33 CIs = [0.26,0.39] | 0.26 CIs = [0.19,0.32] | 0.53 CIs = [0.47,0.59] | 0.52 CIs = [0.46,0.58] | 0.63 CIs = [0.58,0.68] | 0.52 CIs = [0.46,0.58] | 0.34 CIs = [0.27,0.41] | 0.53 CIs = [0.47,0.59] | 0.76 CIs = [0.72,0.81] | 0.62 CIs = [0.57,0.68] | 1.00 CIs = [1.00,1.00] | 0.21 CIs = [0.14,0.27] | 0.21 CIs = [0.15,0.28] | 0.08 CIs = [0.03,0.15] | 0.33 CIs = [0.26,0.40] | 0.47 CIs = [0.41,0.53] | 0.30 CIs = [0.24,0.36] | 0.18 CIs = [0.11,0.25] | 0.22 CIs = [0.16,0.28] | 0.30 CIs = [0.23,0.36] |
| cardiologist 9 | 0.39 CIs = [0.32,0.46] | 0.36 CIs = [0.29,0.42] | 0.29 CIs = [0.23,0.37] | 0.21 CIs = [0.14,0.28] | 0.23 CIs = [0.16,0.30] | 0.37 CIs = [0.31,0.45] | 0.32 CIs = [0.25,0.39] | 0.31 CIs = [0.23,0.38] | 0.23 CIs = [0.16,0.30] | 0.21 CIs = [0.13,0.28] | 0.21 CIs = [0.13,0.27] | 1.00 CIs = [1.00,1.00] | 0.68 CIs = [0.63,0.74] | -0.03 CIs = [-0.10,0.03] | 0.21 CIs = [0.14,0.28] | 0.20 CIs = [0.13,0.26] | 0.19 CIs = [0.13,0.24] | 0.18 CIs = [0.11,0.25] | 0.09 CIs = [0.02,0.17] | 0.19 CIs = [0.12,0.25] |
| cardiologist 10 | 0.44 CIs = [0.37,0.50] | 0.39 CIs = [0.32,0.46] | 0.30 CIs = [0.23,0.37] | 0.21 CIs = [0.14,0.29] | 0.24 CIs = [0.18,0.32] | 0.36 CIs = [0.28,0.43] | 0.35 CIs = [0.28,0.41] | 0.31 CIs = [0.24,0.38] | 0.23 CIs = [0.16,0.30] | 0.22 CIs = [0.15,0.29] | 0.22 CIs = [0.15,0.28] | 0.68 CIs = [0.63,0.74] | 1.00 CIs = [1.00,1.00] | -0.04 CIs = [-0.10,0.03] | 0.19 CIs = [0.12,0.27] | 0.23 CIs = [0.16,0.29] | 0.22 CIs = [0.16,0.28] | 0.17 CIs = [0.10,0.24] | 0.09 CIs = [0.02,0.15] | 0.17 CIs = [0.10,0.24] |
| cardiologist 11 | -0.01 CIs = [-0.08,0.04] | -0.03 CIs = [-0.10,0.04] | 0.10 CIs = [0.03,0.16] | 0.08 CIs = [0.00,0.14] | 0.09 CIs = [0.03,0.16] | 0.05 CIs = [-0.01,0.13] | 0.07 CIs = [0.01,0.13] | 0.05 CIs = [-0.01,0.13] | 0.12 CIs = [0.06,0.18] | 0.10 CIs = [0.04,0.17] | 0.09 CIs = [0.02,0.14] | -0.04 CIs = [-0.10,0.03] | -0.04 CIs = [-0.10,0.03] | 1.00 CIs = [1.00,1.00] | 0.13 CIs = [0.07,0.19] | 0.09 CIs = [0.03,0.15] | -0.04 CIs = [-0.09,0.01] | -0.01 CIs = [-0.08,0.05] | 0.05 CIs = [-0.02,0.12] | 0.11 CIs = [0.05,0.18] |
| cardiologist 12 | 0.18 CIs = [0.11,0.25] | 0.15 CIs = [0.08,0.21] | 0.26 CIs = [0.18,0.33] | 0.24 CIs = [0.16,0.30] | 0.33 CIs = [0.26,0.40] | 0.29 CIs = [0.22,0.36] | 0.21 CIs = [0.14,0.28] | 0.28 CIs = [0.21,0.35] | 0.31 CIs = [0.24,0.38] | 0.32 CIs = [0.26,0.39] | 0.33 CIs = [0.26,0.40] | 0.21 CIs = [0.14,0.28] | 0.19 CIs = [0.12,0.26] | 0.13 CIs = [0.06,0.19] | 1.00 CIs = [1.00,1.00] | 0.33 CIs = [0.26,0.40] | 0.19 CIs = [0.14,0.26] | 0.18 CIs = [0.11,0.25] | 0.22 CIs = [0.15,0.28] | 0.87 CIs = [0.83,0.90] |
| cardiologist 13 | 0.29 CIs = [0.23,0.36] | 0.25 CIs = [0.19,0.32] | 0.39 CIs = [0.33,0.46] | 0.37 CIs = [0.31,0.43] | 0.49 CIs = [0.43,0.55] | 0.41 CIs = [0.35,0.48] | 0.33 CIs = [0.26,0.40] | 0.41 CIs = [0.35,0.47] | 0.47 CIs = [0.40,0.52] | 0.48 CIs = [0.42,0.54] | 0.47 CIs = [0.40,0.53] | 0.20 CIs = [0.13,0.26] | 0.23 CIs = [0.16,0.29] | 0.09 CIs = [0.03,0.14] | 0.33 CIs = [0.27,0.40] | 1.00 CIs = [1.00,1.00] | 0.28 CIs = [0.21,0.34] | 0.21 CIs = [0.14,0.27] | 0.24 CIs = [0.18,0.30] | 0.36 CIs = [0.30,0.43] |
| cardiologist 14 | 0.36 CIs = [0.30,0.42] | 0.30 CIs = [0.25,0.36] | 0.29 CIs = [0.24,0.35] | 0.25 CIs = [0.19,0.30] | 0.25 CIs = [0.19,0.31] | 0.25 CIs = [0.20,0.31] | 0.35 CIs = [0.29,0.41] | 0.22 CIs = [0.17,0.27] | 0.32 CIs = [0.26,0.38] | 0.25 CIs = [0.18,0.31] | 0.30 CIs = [0.23,0.36] | 0.18 CIs = [0.13,0.25] | 0.22 CIs = [0.16,0.28] | -0.04 CIs = [-0.09,0.01] | 0.20 CIs = [0.13,0.26] | 0.28 CIs = [0.21,0.35] | 1.00 CIs = [1.00,1.00] | 0.15 CIs = [0.09,0.21] | 0.06 CIs = [0.01,0.12] | 0.18 CIs = [0.11,0.24] |
| cardiologist 15 | 0.23 CIs = [0.16,0.30] | 0.18 CIs = [0.11,0.26] | 0.13 CIs = [0.06,0.20] | 0.06 CIs = [-0.01,0.14] | 0.14 CIs = [0.07,0.21] | 0.16 CIs = [0.08,0.23] | 0.21 CIs = [0.14,0.27] | 0.19 CIs = [0.12,0.26] | 0.15 CIs = [0.08,0.22] | 0.13 CIs = [0.06,0.20] | 0.18 CIs = [0.11,0.25] | 0.18 CIs = [0.12,0.25] | 0.17 CIs = [0.10,0.24] | -0.01 CIs = [-0.08,0.05] | 0.18 CIs = [0.11,0.24] | 0.21 CIs = [0.14,0.27] | 0.15 CIs = [0.09,0.21] | 1.00 CIs = [1.00,1.00] | 0.11 CIs = [0.04,0.17] | 0.17 CIs = [0.10,0.24] |
| cardiologist 16 | 0.03 CIs = [-0.03,0.11] | 0.05 CIs = [-0.02,0.12] | 0.11 CIs = [0.04,0.19] | 0.16 CIs = [0.08,0.23] | 0.27 CIs = [0.20,0.33] | 0.14 CIs = [0.07,0.22] | 0.05 CIs = [0.00,0.12] | 0.19 CIs = [0.11,0.26] | 0.19 CIs = [0.12,0.26] | 0.24 CIs = [0.18,0.31] | 0.22 CIs = [0.16,0.29] | 0.09 CIs = [0.02,0.16] | 0.09 CIs = [0.02,0.16] | 0.05 CIs = [-0.02,0.12] | 0.21 CIs = [0.15,0.28] | 0.24 CIs = [0.18,0.30] | 0.06 CIs = [0.01,0.12] | 0.11 CIs = [0.03,0.18] | 1.00 CIs = [1.00,1.00] | 0.21 CIs = [0.15,0.28] |
| cardiologist 17 | 0.19 CIs = [0.12,0.27] | 0.15 CIs = [0.08,0.22] | 0.27 CIs = [0.21,0.34] | 0.27 CIs = [0.20,0.34] | 0.32 CIs = [0.25,0.39] | 0.32 CIs = [0.25,0.38] | 0.21 CIs = [0.14,0.27] | 0.29 CIs = [0.23,0.36] | 0.30 CIs = [0.24,0.37] | 0.31 CIs = [0.24,0.38] | 0.30 CIs = [0.23,0.36] | 0.19 CIs = [0.11,0.25] | 0.17 CIs = [0.10,0.24] | 0.11 CIs = [0.05,0.16] | 0.87 CIs = [0.84,0.90] | 0.37 CIs = [0.30,0.43] | 0.18 CIs = [0.11,0.24] | 0.17 CIs = [0.10,0.24] | 0.21 CIs = [0.15,0.27] | 1.00 CIs = [1.00,1.00] |

**Table S5f. Inter-cardiologists and AIEchoDx model variability when diagnosing Normal and non-Normal patients estimated with the Cohen’s Kappa statistic**

|  | Ground-true | AIEchoDx | Consensus | cardiologist 1 | cardiologist 2 | cardiologist 3 | cardiologist 4 | cardiologist 5 | cardiologist 6 | cardiologist 7 | cardiologist 8 | cardiologist 9 | cardiologist 10 | cardiologist 11 | cardiologist 12 | cardiologist 13 | cardiologist 14 | cardiologist 15 | cardiologist 16 | cardiologist 17 |
| --- | --- | --- | --- | --- | --- | --- | --- | --- | --- | --- | --- | --- | --- | --- | --- | --- | --- | --- | --- | --- |
| Ground-true | 1.00 CIs = [1.00,1.00] | 0.87 CIs = [0.82,0.91] | 0.71 CIs = [0.65,0.76] | 0.56 CIs = [0.50,0.62] | 0.52 CIs = [0.45,0.58] | 0.58 CIs = [0.52,0.64] | 0.73 CIs = [0.66,0.79] | 0.58 CIs = [0.52,0.65] | 0.55 CIs = [0.48,0.61] | 0.51 CIs = [0.44,0.58] | 0.53 CIs = [0.47,0.60] | 0.53 CIs = [0.46,0.60] | 0.59 CIs = [0.52,0.67] | 0.33 CIs = [0.27,0.38] | 0.37 CIs = [0.29,0.45] | 0.38 CIs = [0.30,0.46] | 0.53 CIs = [0.44,0.60] | 0.60 CIs = [0.52,0.68] | 0.24 CIs = [0.15,0.32] | 0.33 CIs = [0.25,0.41] |
| AIEchoDx | 0.87 CIs = [0.82,0.92] | 1.00 CIs = [1.00,1.00] | 0.64 CIs = [0.58,0.70] | 0.52 CIs = [0.46,0.59] | 0.48 CIs = [0.41,0.55] | 0.54 CIs = [0.47,0.61] | 0.62 CIs = [0.54,0.68] | 0.51 CIs = [0.43,0.57] | 0.53 CIs = [0.46,0.60] | 0.49 CIs = [0.42,0.55] | 0.51 CIs = [0.44,0.57] | 0.49 CIs = [0.41,0.56] | 0.53 CIs = [0.46,0.61] | 0.32 CIs = [0.27,0.38] | 0.32 CIs = [0.24,0.40] | 0.30 CIs = [0.22,0.38] | 0.48 CIs = [0.40,0.55] | 0.48 CIs = [0.40,0.56] | 0.18 CIs = [0.11,0.27] | 0.29 CIs = [0.22,0.37] |
| Consensus | 0.71 CIs = [0.65,0.77] | 0.64 CIs = [0.58,0.70] | 1.00 CIs = [1.00,1.00] | 0.77 CIs = [0.71,0.81] | 0.70 CIs = [0.64,0.75] | 0.85 CIs = [0.81,0.89] | 0.64 CIs = [0.57,0.70] | 0.73 CIs = [0.67,0.78] | 0.70 CIs = [0.65,0.76] | 0.69 CIs = [0.63,0.75] | 0.71 CIs = [0.65,0.76] | 0.52 CIs = [0.45,0.59] | 0.51 CIs = [0.44,0.58] | 0.42 CIs = [0.36,0.48] | 0.44 CIs = [0.36,0.51] | 0.48 CIs = [0.40,0.55] | 0.38 CIs = [0.31,0.45] | 0.44 CIs = [0.37,0.52] | 0.23 CIs = [0.15,0.30] | 0.40 CIs = [0.32,0.48] |
| cardiologist 1 | 0.56 CIs = [0.50,0.62] | 0.53 CIs = [0.46,0.59] | 0.77 CIs = [0.72,0.82] | 1.00 CIs = [1.00,1.00] | 0.66 CIs = [0.60,0.71] | 0.63 CIs = [0.57,0.69] | 0.43 CIs = [0.37,0.50] | 0.67 CIs = [0.60,0.72] | 0.76 CIs = [0.70,0.80] | 0.66 CIs = [0.60,0.71] | 0.76 CIs = [0.71,0.80] | 0.41 CIs = [0.34,0.48] | 0.41 CIs = [0.35,0.48] | 0.40 CIs = [0.35,0.47] | 0.42 CIs = [0.35,0.49] | 0.42 CIs = [0.36,0.49] | 0.30 CIs = [0.23,0.37] | 0.34 CIs = [0.28,0.41] | 0.25 CIs = [0.18,0.31] | 0.38 CIs = [0.31,0.45] |
| cardiologist 2 | 0.51 CIs = [0.45,0.58] | 0.48 CIs = [0.41,0.55] | 0.70 CIs = [0.64,0.75] | 0.66 CIs = [0.60,0.71] | 1.00 CIs = [1.00,1.00] | 0.65 CIs = [0.59,0.71] | 0.49 CIs = [0.43,0.56] | 0.58 CIs = [0.51,0.64] | 0.70 CIs = [0.65,0.75] | 0.99 CIs = [0.98,1.00] | 0.68 CIs = [0.63,0.74] | 0.39 CIs = [0.32,0.46] | 0.42 CIs = [0.35,0.49] | 0.38 CIs = [0.32,0.44] | 0.38 CIs = [0.31,0.45] | 0.41 CIs = [0.35,0.48] | 0.30 CIs = [0.23,0.37] | 0.31 CIs = [0.24,0.39] | 0.26 CIs = [0.19,0.33] | 0.37 CIs = [0.30,0.44] |
| cardiologist 3 | 0.59 CIs = [0.52,0.65] | 0.54 CIs = [0.47,0.61] | 0.85 CIs = [0.81,0.89] | 0.63 CIs = [0.57,0.69] | 0.65 CIs = [0.60,0.71] | 1.00 CIs = [1.00,1.00] | 0.48 CIs = [0.41,0.55] | 0.67 CIs = [0.61,0.73] | 0.63 CIs = [0.58,0.70] | 0.65 CIs = [0.60,0.71] | 0.64 CIs = [0.57,0.69] | 0.51 CIs = [0.44,0.58] | 0.47 CIs = [0.39,0.53] | 0.46 CIs = [0.41,0.52] | 0.42 CIs = [0.34,0.48] | 0.45 CIs = [0.38,0.52] | 0.28 CIs = [0.20,0.35] | 0.37 CIs = [0.30,0.44] | 0.22 CIs = [0.14,0.29] | 0.39 CIs = [0.33,0.47] |
| cardiologist 4 | 0.73 CIs = [0.67,0.80] | 0.62 CIs = [0.55,0.69] | 0.64 CIs = [0.57,0.70] | 0.44 CIs = [0.37,0.50] | 0.49 CIs = [0.43,0.56] | 0.48 CIs = [0.41,0.55] | 1.00 CIs = [1.00,1.00] | 0.48 CIs = [0.41,0.56] | 0.42 CIs = [0.36,0.49] | 0.50 CIs = [0.43,0.57] | 0.43 CIs = [0.36,0.50] | 0.46 CIs = [0.39,0.54] | 0.52 CIs = [0.44,0.60] | 0.25 CIs = [0.20,0.30] | 0.32 CIs = [0.24,0.40] | 0.33 CIs = [0.25,0.42] | 0.45 CIs = [0.37,0.54] | 0.49 CIs = [0.40,0.58] | 0.25 CIs = [0.16,0.34] | 0.29 CIs = [0.21,0.36] |
| cardiologist 5 | 0.58 CIs = [0.51,0.65] | 0.50 CIs = [0.43,0.58] | 0.73 CIs = [0.67,0.79] | 0.66 CIs = [0.61,0.72] | 0.58 CIs = [0.51,0.64] | 0.67 CIs = [0.60,0.72] | 0.48 CIs = [0.40,0.55] | 1.00 CIs = [1.00,1.00] | 0.66 CIs = [0.60,0.72] | 0.58 CIs = [0.51,0.64] | 0.61 CIs = [0.54,0.67] | 0.46 CIs = [0.39,0.53] | 0.54 CIs = [0.47,0.61] | 0.39 CIs = [0.34,0.45] | 0.47 CIs = [0.40,0.53] | 0.46 CIs = [0.39,0.53] | 0.35 CIs = [0.28,0.43] | 0.38 CIs = [0.30,0.45] | 0.27 CIs = [0.20,0.35] | 0.42 CIs = [0.35,0.50] |
| cardiologist 6 | 0.55 CIs = [0.49,0.61] | 0.53 CIs = [0.47,0.60] | 0.71 CIs = [0.65,0.75] | 0.76 CIs = [0.71,0.80] | 0.70 CIs = [0.64,0.75] | 0.64 CIs = [0.58,0.69] | 0.43 CIs = [0.36,0.49] | 0.66 CIs = [0.60,0.71] | 1.00 CIs = [1.00,1.00] | 0.69 CIs = [0.64,0.75] | 0.83 CIs = [0.78,0.87] | 0.42 CIs = [0.35,0.49] | 0.42 CIs = [0.35,0.48] | 0.43 CIs = [0.37,0.49] | 0.42 CIs = [0.35,0.49] | 0.42 CIs = [0.35,0.49] | 0.32 CIs = [0.25,0.39] | 0.35 CIs = [0.29,0.42] | 0.24 CIs = [0.17,0.31] | 0.41 CIs = [0.33,0.48] |
| cardiologist 7 | 0.51 CIs = [0.44,0.59] | 0.49 CIs = [0.41,0.56] | 0.69 CIs = [0.63,0.75] | 0.66 CIs = [0.60,0.72] | 0.99 CIs = [0.98,1.00] | 0.65 CIs = [0.59,0.71] | 0.50 CIs = [0.43,0.57] | 0.58 CIs = [0.51,0.64] | 0.69 CIs = [0.64,0.75] | 1.00 CIs = [1.00,1.00] | 0.67 CIs = [0.61,0.73] | 0.39 CIs = [0.31,0.46] | 0.42 CIs = [0.34,0.48] | 0.37 CIs = [0.31,0.43] | 0.37 CIs = [0.30,0.44] | 0.40 CIs = [0.33,0.48] | 0.29 CIs = [0.21,0.35] | 0.31 CIs = [0.24,0.38] | 0.26 CIs = [0.19,0.33] | 0.36 CIs = [0.29,0.43] |
| cardiologist 8 | 0.54 CIs = [0.47,0.59] | 0.51 CIs = [0.44,0.57] | 0.70 CIs = [0.65,0.76] | 0.75 CIs = [0.70,0.81] | 0.68 CIs = [0.63,0.73] | 0.64 CIs = [0.58,0.69] | 0.43 CIs = [0.36,0.50] | 0.61 CIs = [0.55,0.67] | 0.83 CIs = [0.78,0.87] | 0.67 CIs = [0.62,0.73] | 1.00 CIs = [1.00,1.00] | 0.40 CIs = [0.33,0.47] | 0.38 CIs = [0.32,0.45] | 0.43 CIs = [0.37,0.50] | 0.41 CIs = [0.34,0.49] | 0.42 CIs = [0.35,0.49] | 0.30 CIs = [0.24,0.37] | 0.33 CIs = [0.26,0.39] | 0.24 CIs = [0.17,0.31] | 0.40 CIs = [0.33,0.47] |
| cardiologist 9 | 0.53 CIs = [0.46,0.60] | 0.48 CIs = [0.42,0.56] | 0.52 CIs = [0.45,0.59] | 0.41 CIs = [0.35,0.48] | 0.39 CIs = [0.32,0.46] | 0.51 CIs = [0.45,0.58] | 0.46 CIs = [0.39,0.54] | 0.46 CIs = [0.39,0.53] | 0.42 CIs = [0.34,0.49] | 0.39 CIs = [0.31,0.46] | 0.40 CIs = [0.33,0.47] | 1.00 CIs = [1.00,1.00] | 0.70 CIs = [0.64,0.76] | 0.32 CIs = [0.26,0.38] | 0.46 CIs = [0.39,0.54] | 0.34 CIs = [0.26,0.42] | 0.27 CIs = [0.18,0.35] | 0.36 CIs = [0.28,0.44] | 0.24 CIs = [0.17,0.33] | 0.46 CIs = [0.38,0.53] |
| cardiologist 10 | 0.60 CIs = [0.52,0.67] | 0.54 CIs = [0.46,0.61] | 0.51 CIs = [0.44,0.58] | 0.41 CIs = [0.35,0.48] | 0.42 CIs = [0.35,0.49] | 0.47 CIs = [0.40,0.54] | 0.52 CIs = [0.44,0.60] | 0.54 CIs = [0.47,0.61] | 0.41 CIs = [0.35,0.48] | 0.42 CIs = [0.35,0.49] | 0.39 CIs = [0.32,0.46] | 0.70 CIs = [0.64,0.76] | 1.00 CIs = [1.00,1.00] | 0.30 CIs = [0.25,0.35] | 0.41 CIs = [0.33,0.49] | 0.32 CIs = [0.23,0.40] | 0.39 CIs = [0.30,0.48] | 0.44 CIs = [0.36,0.53] | 0.26 CIs = [0.17,0.34] | 0.38 CIs = [0.30,0.46] |
| cardiologist 11 | 0.33 CIs = [0.27,0.39] | 0.32 CIs = [0.27,0.38] | 0.42 CIs = [0.36,0.48] | 0.41 CIs = [0.34,0.47] | 0.38 CIs = [0.32,0.44] | 0.46 CIs = [0.41,0.52] | 0.25 CIs = [0.20,0.30] | 0.39 CIs = [0.34,0.45] | 0.43 CIs = [0.37,0.49] | 0.37 CIs = [0.31,0.44] | 0.43 CIs = [0.37,0.49] | 0.32 CIs = [0.26,0.38] | 0.30 CIs = [0.25,0.35] | 1.00 CIs = [1.00,1.00] | 0.36 CIs = [0.31,0.42] | 0.26 CIs = [0.21,0.31] | 0.15 CIs = [0.11,0.20] | 0.23 CIs = [0.18,0.28] | 0.20 CIs = [0.16,0.26] | 0.31 CIs = [0.25,0.37] |
| cardiologist 12 | 0.37 CIs = [0.29,0.45] | 0.32 CIs = [0.24,0.40] | 0.44 CIs = [0.36,0.51] | 0.42 CIs = [0.35,0.49] | 0.38 CIs = [0.30,0.45] | 0.41 CIs = [0.34,0.49] | 0.33 CIs = [0.24,0.41] | 0.47 CIs = [0.40,0.54] | 0.42 CIs = [0.35,0.49] | 0.37 CIs = [0.30,0.44] | 0.41 CIs = [0.34,0.48] | 0.46 CIs = [0.39,0.54] | 0.41 CIs = [0.33,0.49] | 0.36 CIs = [0.31,0.42] | 1.00 CIs = [1.00,1.00] | 0.37 CIs = [0.29,0.45] | 0.21 CIs = [0.13,0.29] | 0.30 CIs = [0.22,0.38] | 0.30 CIs = [0.23,0.39] | 0.87 CIs = [0.83,0.91] |
| cardiologist 13 | 0.38 CIs = [0.30,0.46] | 0.30 CIs = [0.22,0.38] | 0.48 CIs = [0.41,0.55] | 0.42 CIs = [0.36,0.49] | 0.42 CIs = [0.34,0.49] | 0.45 CIs = [0.38,0.52] | 0.33 CIs = [0.25,0.42] | 0.46 CIs = [0.39,0.54] | 0.42 CIs = [0.35,0.49] | 0.40 CIs = [0.33,0.47] | 0.42 CIs = [0.36,0.49] | 0.34 CIs = [0.26,0.41] | 0.32 CIs = [0.23,0.40] | 0.26 CIs = [0.21,0.32] | 0.37 CIs = [0.29,0.45] | 1.00 CIs = [1.00,1.00] | 0.24 CIs = [0.16,0.32] | 0.29 CIs = [0.19,0.37] | 0.24 CIs = [0.16,0.33] | 0.38 CIs = [0.30,0.46] |
| cardiologist 14 | 0.53 CIs = [0.44,0.60] | 0.48 CIs = [0.40,0.55] | 0.38 CIs = [0.31,0.46] | 0.30 CIs = [0.23,0.36] | 0.30 CIs = [0.23,0.37] | 0.28 CIs = [0.20,0.35] | 0.46 CIs = [0.37,0.54] | 0.35 CIs = [0.27,0.42] | 0.32 CIs = [0.25,0.39] | 0.29 CIs = [0.21,0.35] | 0.30 CIs = [0.24,0.36] | 0.27 CIs = [0.20,0.35] | 0.39 CIs = [0.30,0.48] | 0.15 CIs = [0.10,0.20] | 0.21 CIs = [0.13,0.29] | 0.24 CIs = [0.16,0.33] | 1.00 CIs = [1.00,1.00] | 0.32 CIs = [0.23,0.41] | 0.18 CIs = [0.10,0.27] | 0.16 CIs = [0.08,0.24] |
| cardiologist 15 | 0.60 CIs = [0.52,0.67] | 0.48 CIs = [0.41,0.56] | 0.44 CIs = [0.37,0.51] | 0.34 CIs = [0.28,0.40] | 0.31 CIs = [0.24,0.38] | 0.37 CIs = [0.30,0.44] | 0.49 CIs = [0.40,0.57] | 0.38 CIs = [0.30,0.46] | 0.35 CIs = [0.28,0.42] | 0.31 CIs = [0.23,0.38] | 0.33 CIs = [0.26,0.39] | 0.36 CIs = [0.28,0.44] | 0.44 CIs = [0.35,0.53] | 0.23 CIs = [0.18,0.28] | 0.30 CIs = [0.22,0.38] | 0.29 CIs = [0.20,0.37] | 0.32 CIs = [0.22,0.41] | 1.00 CIs = [1.00,1.00] | 0.24 CIs = [0.16,0.34] | 0.29 CIs = [0.20,0.36] |
| cardiologist 16 | 0.23 CIs = [0.15,0.32] | 0.19 CIs = [0.11,0.26] | 0.23 CIs = [0.15,0.30] | 0.25 CIs = [0.18,0.31] | 0.26 CIs = [0.19,0.33] | 0.22 CIs = [0.15,0.29] | 0.25 CIs = [0.17,0.34] | 0.27 CIs = [0.20,0.36] | 0.24 CIs = [0.17,0.31] | 0.26 CIs = [0.19,0.33] | 0.24 CIs = [0.17,0.31] | 0.25 CIs = [0.17,0.33] | 0.26 CIs = [0.17,0.35] | 0.21 CIs = [0.15,0.26] | 0.31 CIs = [0.22,0.39] | 0.24 CIs = [0.16,0.33] | 0.18 CIs = [0.10,0.27] | 0.25 CIs = [0.16,0.33] | 1.00 CIs = [1.00,1.00] | 0.29 CIs = [0.21,0.37] |
| cardiologist 17 | 0.33 CIs = [0.25,0.40] | 0.29 CIs = [0.22,0.37] | 0.40 CIs = [0.33,0.47] | 0.38 CIs = [0.31,0.45] | 0.36 CIs = [0.30,0.44] | 0.40 CIs = [0.32,0.47] | 0.29 CIs = [0.21,0.37] | 0.42 CIs = [0.35,0.50] | 0.40 CIs = [0.33,0.47] | 0.36 CIs = [0.29,0.43] | 0.40 CIs = [0.33,0.47] | 0.46 CIs = [0.38,0.53] | 0.38 CIs = [0.30,0.45] | 0.31 CIs = [0.26,0.37] | 0.87 CIs = [0.83,0.91] | 0.39 CIs = [0.31,0.47] | 0.16 CIs = [0.08,0.24] | 0.29 CIs = [0.20,0.36] | 0.29 CIs = [0.21,0.38] | 1.00 CIs = [1.00,1.00] |

**Table S6. Clinical characteristics of phenogroups of DCM auto-categorized by AIEchoDx, Related to Figure 5.**

|  | DCM-low (n=32) | DCM-high (n=33) | P-value |
| --- | --- | --- | --- |
| **Demographic and Vital signs** | |  |  |
| Male (%) | 30 (90.9%) | 28 (87.5%) | 0.669 |
| Age (years) | 50±4 | 50±2 | 0.293 |
| In hospital days | 12±1 | 11±1 | 0.206 |
| SBP (mmHg) | 134±3 | 117±2 | <0.001 |
| DBP (mmHg) | 78±3 | 79±3 | 0.536 |
| Pulse (b.p.m) | 80±2 | 91±4 | 0.128 |
| Breathe (b.p.m) | 18±0 | 18±0 | 0.228 |
| Temprature (℃) | 36.3±0.1 | 36.3±0.0 | 0.867 |
| Height (cm) | 173±1 | 171±1 | 0.756 |
| Weight (kg) | 82±3 | 76±2 | 0.481 |
| BMI (kg/m2) | 27±1 | 26±1 | 0.844 |
| BSA (m2) | 2.06±0.04 | 1.98±0.03 | 0.482 |
| Alcohol (%) | 10 (30.3%) | 14 (43.8%) | 0.262 |
| Smoke (%) | 8 (24.2%) | 11 (34.4%) | 0.368 |
| **Echocardiographic** | |  |  |
| LVEDV (ml) | 174±12 | 234±9 | <0.001 |
| LVESV (ml) | 114±9 | 163±8 | <0.001 |
| LVEF (%) | 35±1 | 29±1 | 0.001 |
| IVS (mm) | 10.9±0.2 | 9.6±0.3 | <0.001 |
| LVPW (mm) | 10.5±0.2 | 9.6±0.3 | 0.006 |
| LV (mm) | 57±2 | 62±1 | 0.021 |
| RV (mm) | 39±1 | 41±1 | 0.581 |
| LV/RV ratio | 1.45±0.03 | 1.55±0.05 | 0.038 |
| LA (mm) | 41±1 | 46±1 | <0.001 |
| RA (mm) | 40±1 | 44±1 | 0.009 |
| LA/RA ratio | 1.09±0.04 | 1.09±0.03 | 0.660 |
| E wave (m/s) | 0.74±0.06 | 0.88±0.05 | 0.110 |
| A wave (m/s) | 0.59±0.04 | 0.57±0.07 | 0.562 |
| E/A ratio | 1.34±0.16 | 2.30±0.35 | 0.043 |
| MR grade |  |  | <0.001 |
| none | 12 (36.4%) | 4 (12.5%) |  |
| mild | 15 (45.5%) | 5 (15.6%) |  |
| moderate | 5 (15.2%) | 7 (21.9%) |  |
| severe | 1 (3.0%) | 16 (50.0% |  |
| TR grade |  |  | 0.003 |
| none | 7 (21.2%) | 1 (3.1%) |  |
| mild | 18 (54.5%) | 15 (46.9%) |  |
| moderate | 8 (24.2%) | 10 (31.3%) |  |
| severe | 0 (0.0%) | 6 (18.8%) |  |
| PR grade |  |  | 0.007 |
| none | 27 (81.8%) | 10 (31.3%) |  |
| mild | 6 (18.2%) | 19 (59.4%) |  |
| moderate | 0 (0.0%) | 2 (6.3%) |  |
| TR peak velocity (m/s) | 2.65±0.19 | 2.84±0.10 | 0.621 |
| PASP (mmHg) | 40±4 | 43±3 | 0.853 |
| PR peak velocity (m/s) | 1.66±0.30 | 2.11±0.13 | 0.076 |
| PAmP (mmHg) | 20±4 | 30±2 | 0.036 |
| **Previous history** |  |  |  |
| Hypertension | 18 (54.5%) | 12 (37.5%) | 0.174 |
| Diabetes | 5 (15.2%) | 8 (25.0%) | 0.320 |
| CAD | 12 (36.4%) | 3 (9.4%) | 0.010 |
| MI | 4 (12.1%) | 1 (3.1%) | 0.181 |
| Hyperlipemia | 3 (9.1%) | 2 (6.3%) | 0.680 |
| Stroke | 1 (3.0%) | 2 (6.3%) | 0.850 |
| NYHA class |  |  | 0.666 |
| I | 3 (9.1%) | 0 (0.0%) |  |
| II | 14 (42.4%) | 13 (40.6%) |  |
| III | 11 (33.3%) | 19 (59.4%) |  |
| IV | 5 (15.2%) | 0 (0.0%) |  |
| **Medication** |  |  |  |
| Diuretics | 7 (21.2%) | 26 (81.3%) | <0.001 |
| ACEI/ARB | 5 (15.2%) | 24 (75.0%) | <0.001 |
| ARNi | 9 (27.3%) | 2 (6.3%) | 0.001 |
| Beta-blocker | 11 (33.3%) | 29 (90.6%) | 0.001 |
| Spironolactone | 16 (48.5%) | 29 (90.6%) | 0.151 |
| Digoxin | 3 (9.1%) | 21 (65.6%) | <0.001 |
| ICD/CRT | 0 (0.0%) | 5 (15.6%) | 0.064 |
| **Heart rhythm** |  |  |  |
| Sinus | 13 (39.4%) | 25 (78.1%) | 0.220 |
| LBBB | 5 (15.2%) | 8 (25.0%) | 0.959 |
| AF | 8 (24.2%) | 9 (28.1%) | 0.717 |
| **Laboratory tests** | |  |  |
| Hemoglobin (g/L) | 128±7 | 148±3 | 0.032 |
| NT-proBNP (pg/ml) | 2712.9±1088.6 | 5079.8±749.5 | <0.001 |
| cTnT (ng/ml) | 0.028±0.006 | 0.024±0.003 | 0.778 |
| CK (U/L) | 75.5±10.3 | 87.6±8.9 | 0.090 |
| CK-MB (U/L) | 2.64±0.72 | 2.59±0.40 | 0.037 |
| Cr (umol/L) | 123.1±28.3 | 104.4±4.9 | 0.009 |
| Glu (mmol/L) | 5.81±0.42 | 6.01±0.28 | 0.158 |
| TG (mmol/L) | 1.17±0.10 | 1.33±0.20 | 0.993 |
| TC (mmol/L) | 3.47±0.22 | 3.66±0.12 | 0.216 |
| HDL (mmol/L) | 0.97±0.05 | 0.92±0.04 | 0.295 |
| LDL (mmol/L) | 2.17±0.21 | 2.37±0.09 | 0.105 |

Abbreviation: DCM, dilated cardiomyopathy; SBP, systolic blood pressure; DBP, diastolic blood pressure; b.p.m, beats per minute; BMI, body mass index; BSA, body surface area; LVEDV, left ventricular end diastolic volume; LVESV, left ventricular end systolic volume; LVEF, left ventricular ejection fraction; IVS, interventricular septum; LVPW, left ventricular posterior wall; LV, left ventricular; RV, right ventricular; LA, left atrium; RA, right atrium; MR, mitral regurgitation; TR, tricuspid regurgitation; PR, pulmonary regurgitation; PASP, pulmonary artery systolic pressure; PAmP, pulmonary artery mean pressure; CAD, coronary arterial disease; MI, myocardial infarction; NYHA, New York Heart Association; ACEI, angiotensin-converting enzyme inhibitor; ARB, angiotensin receptor blocker; ANRi, angiotensin receptor-neprilysin inhibitor; ICD, implantable cardioverter defibrillator; CRT, cardiac resynchronization therapy; LBBB, left bundle branch block; AF, atrial fibrillation; NT-proBNP, N-terminal of the prohormone brain natriuretic peptide; cTnT, cardiac troponin T; CK, creatine kinase; CK-MB, creatine kinase isoenzyme MB; Cr, creatine; TG, triglyceride; TC, total cholesterol; HDL, high density lipoprotein cholesterol; LDL, low density lipoprotein cholesterol.

**Supplementary Movie 1. Echocardiogram videos of one typical ASD patient and its CAM.**

**Supplementary Movie 2. Echocardiogram videos of one typical DCM patient and its CAM.**

**Supplementary Movie 3. Echocardiogram videos of one typical HCM patient and its CAM.**

**Supplementary Movie 4. Echocardiogram videos of one typical prior MI patient and its CAM.**

**Supplementary Movie 5. Echocardiogram videos of one typical DCM-high and DCM-low patient and their CAMs.**
